# A refined model of how *Yersinia pestis* produces a transmissible infection in its flea vector

**DOI:** 10.1371/journal.ppat.1008440

**Published:** 2020-04-15

**Authors:** Amélie Dewitte, Typhanie Bouvenot, François Pierre, Isabelle Ricard, Elizabeth Pradel, Nicolas Barois, Anaïs Hujeux, Sébastien Bontemps-Gallo, Florent Sebbane

**Affiliations:** Univ. Lille, Inserm, CNRS, CHU Lille, Institut Pasteur de Lille, U1019 - UMR9017- CIIL - Center for Infection and Immunity of Lille, Lille, France; Tufts University, UNITED STATES

## Abstract

In flea-borne plague, blockage of the flea's foregut by *Yersinia pestis* hastens transmission to the mammalian host. Based on microscopy observations, we first suggest that flea blockage results from primary infection of the foregut and not from midgut colonization. In this model, flea infection is characterized by the recurrent production of a mass that fills the lumen of the proventriculus and encompasses a large number of *Y*. *pestis*. This recurrence phase ends when the proventricular cast is hard enough to block blood ingestion. We further showed that *ymt* (known to be essential for flea infection) is crucial for cast production, whereas the *hmsHFRS* operon (known to be essential for the formation of the biofilm that blocks the gut) is needed for cast consolidation. By screening a library of mutants (each lacking a locus previously known to be upregulated in the flea gut) for biofilm formation, we found that *rpiA* is important for flea blockage but not for colonization of the midgut. This locus may initially be required to resist toxic compounds within the proventricular cast. However, once the bacterium has adapted to the flea, *rpiA* helps to form the biofilm that consolidates the proventricular cast. Lastly, we used genetic techniques to demonstrate that ribose-5-phosphate isomerase activity (due to the recent gain of a second copy of *rpiA* (*y2892*)) accentuated blockage but not midgut colonization. It is noteworthy that *rpiA* is an ancestral gene, *hmsHFRS* and *rpiA2* were acquired by the recent ancestor of *Y*. *pestis*, and *ymt* was acquired by *Y*. *pestis* itself. Our present results (i) highlight the physiopathological and molecular mechanisms leading to flea blockage, (ii) show that the role of a gene like *rpiA* changes in space and in time during an infection, and (iii) emphasize that evolution is a gradual process punctuated by sudden jumps.

## Author summary

Plague is a deadly flea-borne disease caused by the bacillus *Yersinia pestis*. This bacterium blocks the flea's foregut (proventriculus) to increase its likelihood of being regurgitated by fleas trying to take a blood meal. Understanding the mechanisms leading to flea blockage may help to find ways of controlling plague. Here, we propose a model in which blockage results from primary infection of the foregut and not from midgut colonization. In the proventriculus, *Y*. *pestis* induces, resists and then consolidates the formation of a bactericidal matrix that can be recurrently dislodged by a blood meal before it becomes firmly anchored to the proventriculus and thus definitively obstructs it. Induction, resistance, and then consolidation of the mass in the proventriculus were made possible by the sequential acquisition of genetic material by the ancestors of *Y*. *pestis* and by *Y*. *pestis* itself–emphasizing that evolution is a gradual process punctuated by sudden jumps.

## Introduction

In many parts of the world, the plague bacillus *Yersinia pestis* circulates among wild rodents and their associated fleas [1]. After being ingested by a flea during a blood meal, the bacterium proliferates after having adapted to the physiologic context and defenses of the hematophagous insect’s gut [2–5]. In some flea species, the bacterium also forms a biofilm that clogs the proventriculus—a valve covered with inward-facing spines and that connects the esophagus to the midgut where the blood is digested (S1 Fig) [6–8]. As a result, the flea becomes unable to pump blood beyond the proventriculus. The “blocked” flea starts to starve to death and so frantically bites the mammalian host in an attempt to satisfy its appetite. These fruitless feeding attempts erode the bacterial biofilm, so that some bacteria are eventually dislodged and regurgitated at the feeding site—thus transmitting plague. However, it must be borne in mind that complete blockage is not essential for transmission. Bacot (who discovered the phenomenon of flea blockage) considered that partially blocked fleas are more effective transmitters than completely blocked fleas [9].

The current model of transmissible infection by *Y*. *pestis* in the flea suggests that the bacterium initiates the colonization of the midgut prior to forming the biofilm and thus obstructing the proventriculus [2, 3, 7, 10]. In this model, *Y*. *pestis* factors required for flea-borne transmission of plague can be classified as colonization factors or as transmission factors [2, 3, 7, 10]. The former category includes factors required for bacterial adaptation, growth, and resistance to toxic elements found in the midgut. The transmission factors encompass genes thought to be dedicated to the formation of the flea-blocking bacterial biofilm. Hence, mutants lacking colonization factors are highly deficient in insect colonization, while mutants lacking transmission factors are deficient in blockage but are considered to fully competent (or only slightly deficient) in the colonization of the digestive tract [2, 7].

To date, only 12 loci have been mechanistically implicated in the production of a successful infection in fleas [2, 7, 11–19]. One (*ymt*) is considered to protect the bacterium from a cytotoxic digestion product of blood plasma. Five genes (*oxyR*, *yfbA*, *rovM*, *phoPQ* and *hfq*) encode regulators that activate an as-yet unidentified molecular program *in vivo*. Three other loci (*hmsT*, *hmsP* and *hmsCDE*) regulate the intracellular concentration of cyclic diguanosine monophosphate—a second messenger that controls the formation of the foregut-blocking biofilm by stimulating the production of a β-1,6-N-acetyl-D-glucosamine polymer by the HmsHFRS complex. The OmpR-EnvZ regulatory system (that may be triggered by nutrient depletion in the flea gut) participates also in biofilm formation through the activation of the porin gene *ompF*. The twelfth gene (*gmhA*) encodes the phosphoheptose isomerase required for synthesis of the lipopolysaccharide inner core, which suggests that this factor has an indirect role. Lastly, characterization of the transcriptome of *Y*. *pestis* isolated from fleas has provided clues on the biological roles of the genes upregulated *in vivo* [4]. Among the hundreds of loci activated in the flea gut, only seven (*yidE*, *cpxP*, *tibA*, *cupA*, *pspABC*, *gabTpotDBC*, and *rovM*) have been studied in the flea [4, 15]. Even though it is known that these loci are involved in biofilm formation in other bacteria, only the LysR-type regulator gene *rovM* is needed for flea infection [15]. However, a Δ*rovM* mutant exhibited a lack of competitive fitness relative to the wild type strain (i.e. when it co-infected fleas with the wild type (WT) strain). In summary, our knowledge of *Y*. *pestis*' ability to produce a transmissible infection in fleas remains limited. Hence, with a view to better understanding the mechanisms leading to flea-borne plague, we decided to further investigate the role in fleas of the genes previously found to be upregulated *in vivo*. However, we first characterized the physiopathology of the infection in fleas.

## Results

### Flea infection is characterized by waves of production of a brownish proventricular mass full of *Y*. *pestis* and that can be displaced during feeding

**Fig 1:**
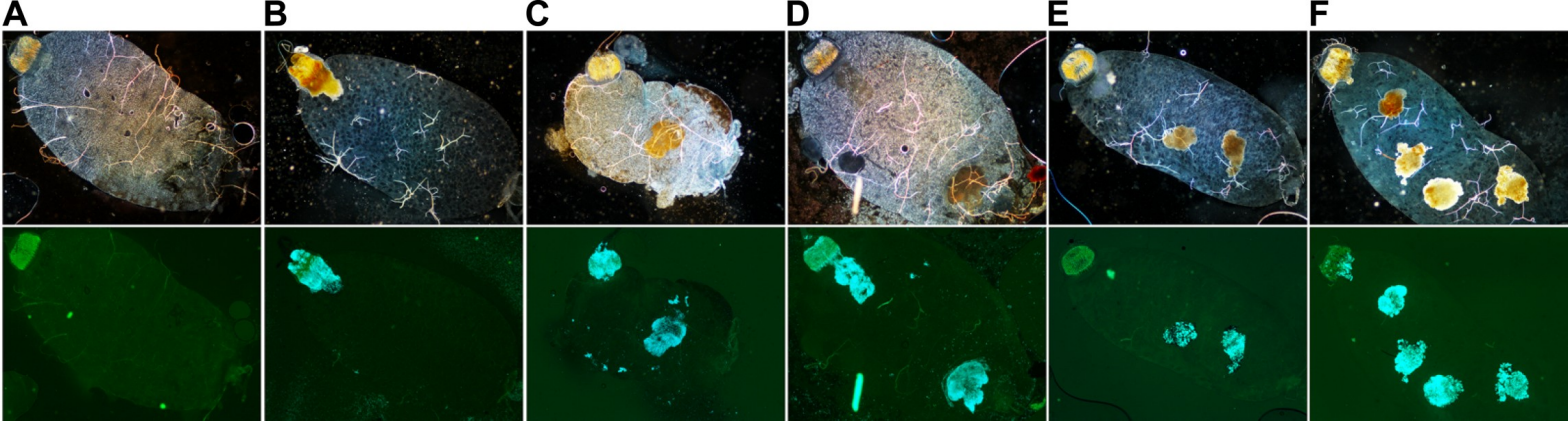
The flea gut infected with *Y*. *pestis* contains zero, one or several brownish masses. Images were acquired using a bright-field microscope (upper images) and a fluorescence microscope (lower images). Using Adobe Photoshop, the fluorescence microscopy images were modified to highlight bacteria (in blue) that were attached (or not) to the proventriculus (in green). After an infection, the flea gut may (B and D) or may not (A) feature a brownish, bacteria-containing mass anchored within the proventriculus. In some cases, one or more free-floating masses (C to F) are observed in the midgut. The images shown here are representative of experiments on WT and mutant strains. The photos shown in panels A to E are representative of what can be seen in the gut of fleas collected 2 and 6 days post-infection, whereas all the panels are representative of what can be seen in the gut of fleas collected 13 days after infection.

**Fig 2:**
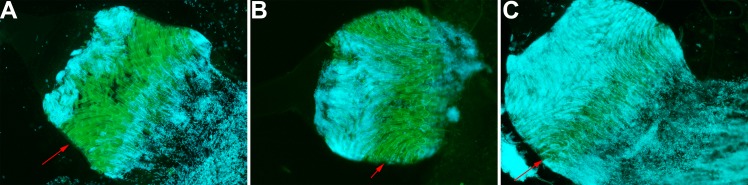
The three-zone colonization of the proventriculus. Fluorescence microscopy images of infected proventriculi taken one day post-infection. The images were modified using Adobe Photoshop to highlight bacteria (in blue) and the proventriculus (in green). The proventriculus contained *Y*. *pestis* in the anterior and posterior spine-bearing regions, and the posterior spineless region of the proventriculus—the so-called stomodaeum valve that telescopes with the midgut (A and C). The red arrowheads indicate a “green buffer zone”; i.e. a central spine-bearing region with no (A), few (B) or some (C) bacteria.

**Fig 3:**
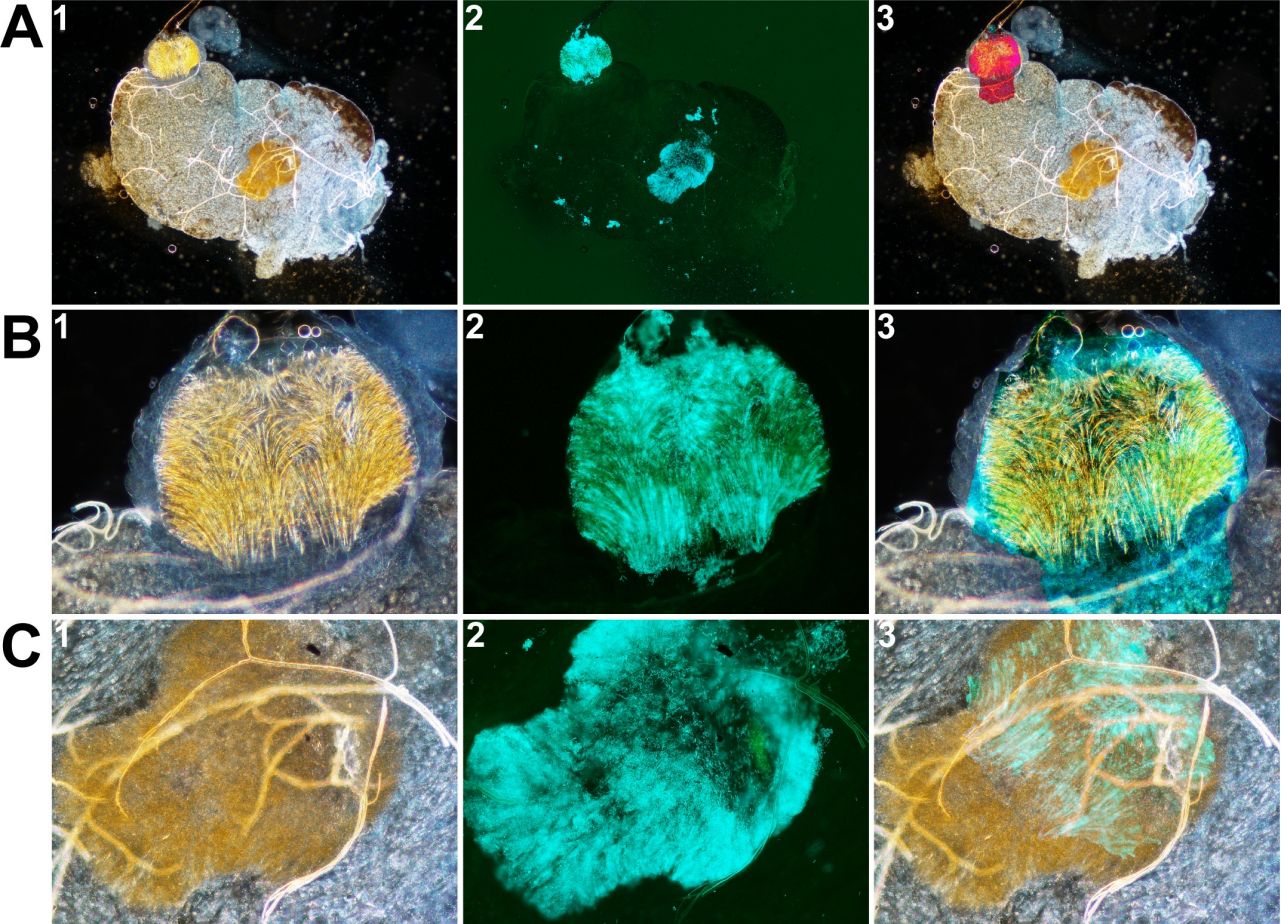
The free-floating mass observed in the midgut is a proventricular cast containing *Y*. *pestis*. Images of the whole flea gut (A), the proventriculus (B) and the free-floating mass (C) contained within the midgut were taken using bright-field (left) and fluorescence (middle) microscopes. The images on the right are merged images generated after appropriate rotation and blending mode (using Photoshop) to show that the free-floating mass is a proventricular cast. Notably, image A3 was produced by merging image A1 with the free-floating mass extracted from A1, and the “difference” blending mode. Images B3 and C3 were produced by merging a bright-field image with the corresponding fluorescence image (i.e. B3 = B1 merged with C2; C3 = C1 merged with B2) and the “lighten” blending mode. Fluorescence images were also modified (using Photoshop) to highlight the bacteria in blue.

To better understand how *Y*. *pestis* produces a transmissible infection in fleas, we first performed a preliminary bright-field and fluorescence microscopy study of the insects’ gut contents at different times (in days) after a meal containing fluorescent *Y*. *pestis*. We observed that on the day after infection, 14 of 20 infected fleas collected at random (70%) contained a brownish mass anchored in the proventriculus (Fig 1A and 1B). The mass always contained a large number of *Y*. *pestis* (Fig 1B). Strikingly, the anterior and posterior halves of the proventriculus were colonized but were separated by a “green buffer zone” that contained no or few bacteria (Fig 2). Interestingly, a free-floating brownish mass full of bacteria and surrounding by planktonic bacteria was often observed in the midgut of fleas that had an uninfected meal after infection (Fig 1C). Regardless of whether or not the proventriculus contains a mass, we often observed several free-floating, brownish masses in the midgut. Furthermore, the number of free-floating masses appeared to increase over time (Fig 1C–1F). Hence, we concluded that the free-floating masses were fresh or eroded bacteria-containing proventricular casts. This idea was supported by bright-field and fluorescence microscopy images showing that the brownish mass sometimes looked like a proventriculus. Indeed, we found that the brownish midgut mass had almost exactly the same shape as the proventriculus (Fig 3). However, bright field microscopy is not always of use in determining whether a free-floating mass located in the midgut has been molded into the shape of the proventriculus because the mass is sometimes shapeless or because the proventricular cast has been dislocated. In such a case, it is essential to use fluorescent microscopy to demonstrate the presence of fluorescent *Y*. *pestis* in a proventricular cast and thus reveal some key anatomic and colonization-related aspects of the proventriculus (see S1 Text and S2 Fig, which describe the method and give several practical examples of cast identification). Hence, the data prompted us to propose a model in which a mass containing *Y*. *pestis* is molded into the shape of the proventriculus very soon after infection. Next, the proventricular cast is washed back into the midgut during feeding. The evacuated mass thus remains in midgut, where the digestion process may erode or even dislocate it. In the meantime, a new mass is molded into the shape of the proventriculus, and the proventriculus is recolonized by *Y*. *pestis*. The repeated production of a proventricular mass containing *Y*. *pestis* might continue until the cast is robust enough to resist incoming blood flow, and thus blocks the flea’s gut.

The above-mentioned “cast” model was further confirmed by our study of proventricular clearance, we determined the percentage of fleas containing one, two or more than two proventricular casts before and immediately after post-infection sterile meals (Fig 9). Indeed, we found that the proportion of infected fleas with a mass anchored within the proventriculus decreased after each feed (Fig 9A, red bars, before *vs*. after). Conversely, the proportion of fleas displaying a “clear” proventriculus plus a proventricular cast in the midgut increased after feeding (Fig 9A, black bars, before *vs*. after). Consistently, the proventricular clearance of fluorescent bacteria was similar to the clearance of the proventricular mass (Fig 9C). Lastly, we found that the percentage of fleas containing at least one proventricular cast in the midgut increased progressively after infection and sharply after a blood meal. In agreement with our “cast model, the number of casts contained in the midgut (black and red bars) increased with the number of meals (Fig 9B).

### A small proportion of fleas might dislodge the mass molded into the shape of the proventriculus without ingesting blood

To further confirm that the masses observed in the midgut are primarily produced in the proventriculus, we scored the masses’ presence and localization (regardless of the size) in the flea gut 1, 3, 6, 12 and 24 hours post-infection (Table 1). One hour after infection, 60% of fleas had a mass in their digestive tract, and this percentage peaked at 12 hours post-infection. One hour post-infection, the mass was only attached to the proventriculus. The mass appeared to be stored in the proventriculus for the next 23 hours. Indeed, 70–75% of fleas collected between 6 and 24 hours post-infection displayed a mass only attached to the proventriculus (Table 1). However, from 3 hours post-infection onwards, 3–10% of the guts displayed a mass anchored to the proventriculus and a mass in the midgut (Table 1). Furthermore, from 6 hours after infection onwards, 13 to 15% of fleas had a mass (of variable shape and size) in their midgut only. With the exception of a mass produced at 6 hours post-infection and whose shape was almost exactly that of a proventriculus (S3 Fig), the mass observed in the midgut of fleas collected between 6 and 12 h post-infection was too small or too amorphous to be identified as a proventricular cast and did not show any fluorescence (presumably due to some quenching). At 24 h post-infection, the mass in the midgut was also shapeless but was identified as a proventricular cast by the presence of fluorescent bacteria. The presence of different types of mass (including a proventricular cast in the midgut prior an uninfected feed) is questionable. The method used to analyze the gut might be at cause. For instance, observation of the dissected gut involves the placement of a cover slip on the sample, which might sometimes dislodge the mass from the proventriculus and push it into the midgut. However, it is also conceivable that a small proportion of fleas could dislodge the growing cast themselves. Taken as a whole, the data suggest that (i) the mass is molded into the shape of the proventriculus within an hour of infection, and (ii) the mass does not originate from the midgut. Furthermore, in a small proportion of the cases soon after infection, the flea might dislodge the proventricular cast from the proventriculus without a blood meal.

**Table 1:** Presence and location of a mass in the infected flea's gut, prior to a sterile meal.

Hours post-infection ^a^	Proportion of infected fleas with mass in the gut	Mass present in the:		
		PV (only)	MG (only)	MG + PV
1	60%	60%	0%	0%
3	70%	60%	0%	10%
6	90%	73%	13%	3%
12	95%	75%	15%	5%
24	90%	70%	15%	5%

^a^, Percentages were determined using 10 fleas collected 1 and 3 hours post-infection, 10 and 20 fleas (from two independent experiments) collected 6 hours post-infection, and 20 fleas collected 12 and 24 hours after infection. MG, midgut; PV, proventriculus.

### The *ymt* and *hmsHFRS* loci are respectively required for the production and then consolidation of a blocking mass (the cast) in the proventriculus

Given that the *hmsHFRS* operon (found in several *Yersinia* species) is considered to produce the biofilm obstructing the proventriculus [7, 10, 20], we suspected that the brownish mass in the proventriculus depended on *hmsHFRS*. However, we found that 70% of the fleas infected with a Δ*hmsHFRS Y*. *pestis* mutant showed a proventricular mass containing many bacilli one day after infection. This result is consistent with a recent report whereby a mass produced independently of the *hmsHFRS* locus is present in the proventriculus within 24 h of infection [21]. Surprisingly, the mass containing bacteria was also observed 13 days post-infection, and the proventriculus was infected in 35% of the fleas. Thus, in the absence of *hmsHFRS*, colonization of the proventriculus remains possible for a prolonged period of time, and so one can reason that a gene other that the *hmsHFRS* operon is required for the production of the brownish proventricular mass. Alternatively, the flea might produce a mass independently of the presence of bacteria in the blood. However, we did not find a mass or bacteria within the proventriculus when fleas fed on blood alone or blood containing *Escherichia coli* (which is unable to infect fleas). It is noteworthy that an earlier study did not report the presence of a mass following flea infection with the recent ancestor of *Y*. *pestis* (*Yersinia pseudotuberculosis*, which is unable to block fleas) [10]. Hence, we hypothesized that the production of a proventricular mass soon after infection requires on *Y*. *pestis* specific genes. We further hypothesized that *ymt* is needed for the production of the proventricular mass because it is the only gene acquired by *Y*. *pestis* (during its emergence from *Y*. *pseudotuberculosis*) that is essential for flea infection [2, 22–24]. Consistently, 24 hours post-infection, we did not observe a mass in the proventriculus and/or the midgut when fleas (n = 20) were fed on blood containing a Δ*ymt* mutant. In contrast, 65% of the fleas (n = 20) infected with the complemented mutant showed a mass anchored to the proventriculus; this is consistent with the above-mentioned data obtained with the WT strain (Table 1). This finding corroborates a previous study in which fleas infected with *Y*. *pseudotuberculosis* expressing *ymt* contained large bacteria-containing masses in their midgut [10]. Taken as a whole, the data suggest that the recent acquisition of *ymt* allows *Y*. *pestis* to (i) induce the production of a bacteria-entrapping mass within the proventriculus, and then (ii) consolidate this mass via the production of a polymer of β-1,6-N-acetyl-D-glucosamine, which is dependent on the ancestral *hmsHFRS* operon. Alternatively, the flea may produce the mass in response to the colonization of its proventriculus by *ymt*-expressing bacteria; in other words, the mass may be part of an immune response.

### Although *rep*, *glpD*, *rpiA*, *hdfR*, *ail* and *rsx* are needed for biofilm formation *in vitro*, only *rpiA* is involved in flea blockage

**Fig 4:**
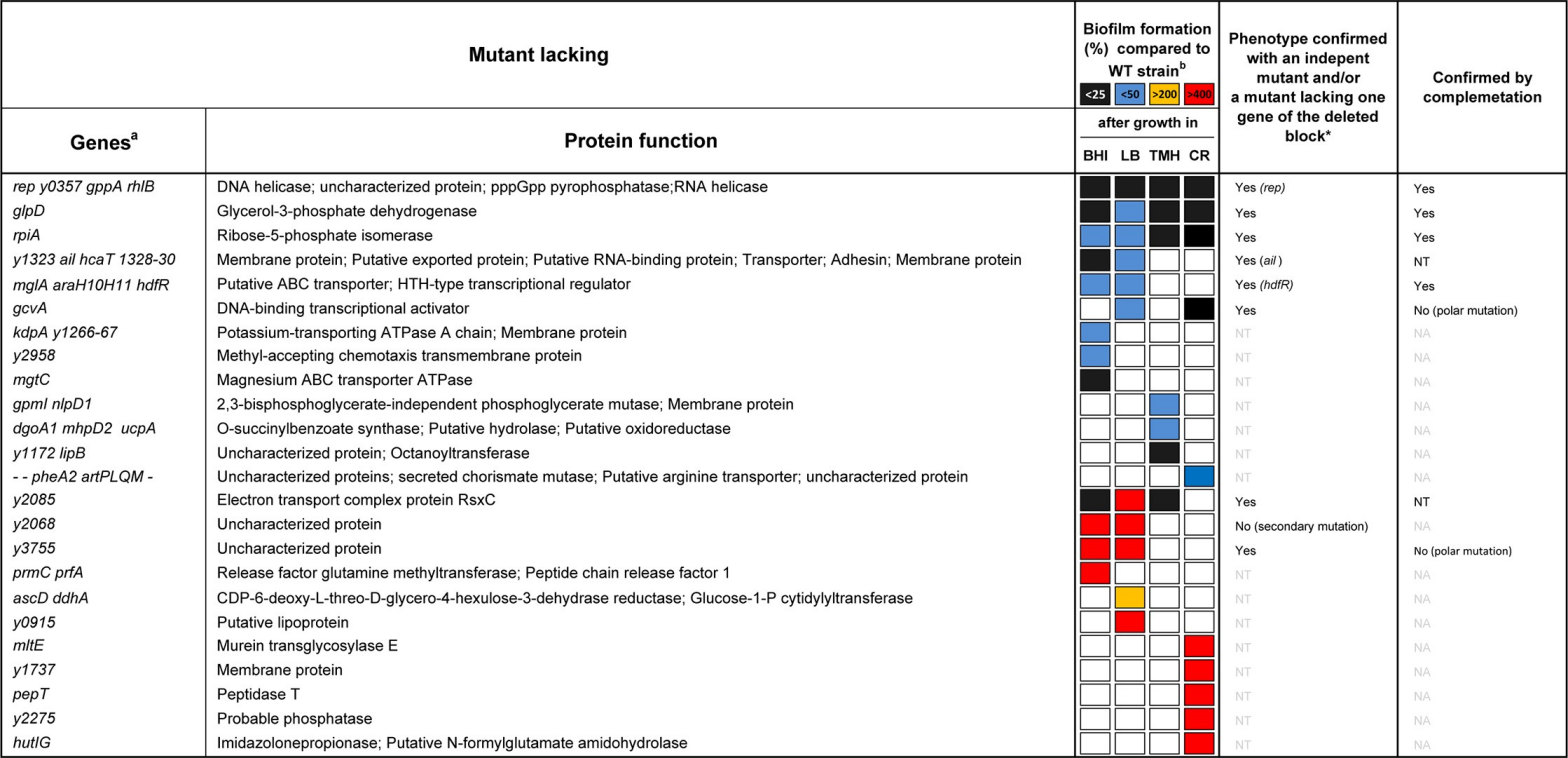
List of *Y*. *pestis* mutants affected in biofilm formation. In the figure, ^a^, "-" means to; ^b^, shown is the biofilm production relative to the parental strain. White squares mean that there was no difference between the mutant and the parental strain. For Congo red plate assay, black, blue and red squares mean that the bacterial colony is white, less red, and redder compared to the parental strain respectively.; *, the gene between the bracket is that responsible for the phenotype. NT, not tested; NA, not applicable.

**Fig 5:**
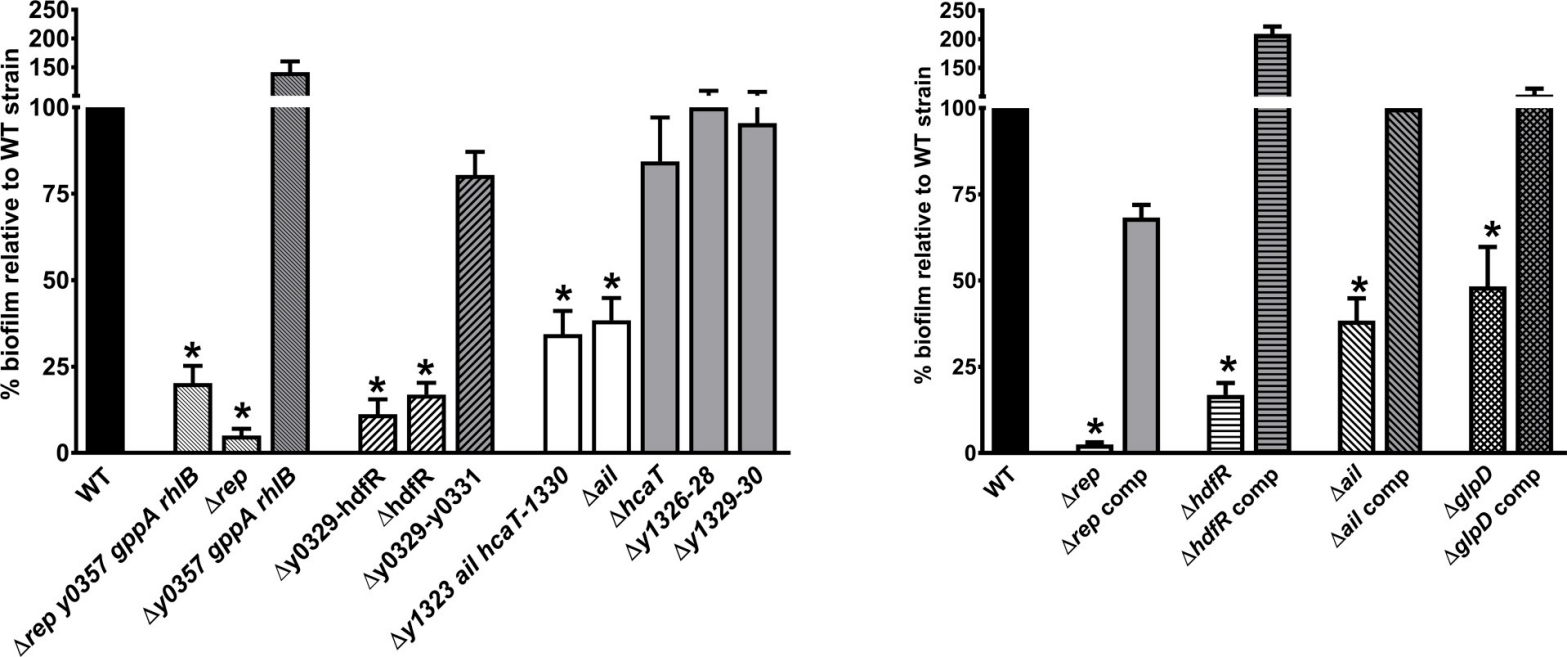
*rep*, *hdfR*, *glpD* and *ail* are required for biofilm formation *in vitro*. Biofilm formation of selected mutants (relative to the WT strain) was measured after a 24-hour incubation at 21°C with shaking in LB supplemented with Ca^2+^ and Mg^2+^ (see Fig 4). The mean ± SEM values from at least three independent experiments are shown. *: mutants producing significantly less biofilm than the WT strain (p<0.01 in a one-way analysis of variance).

**Fig 6:**
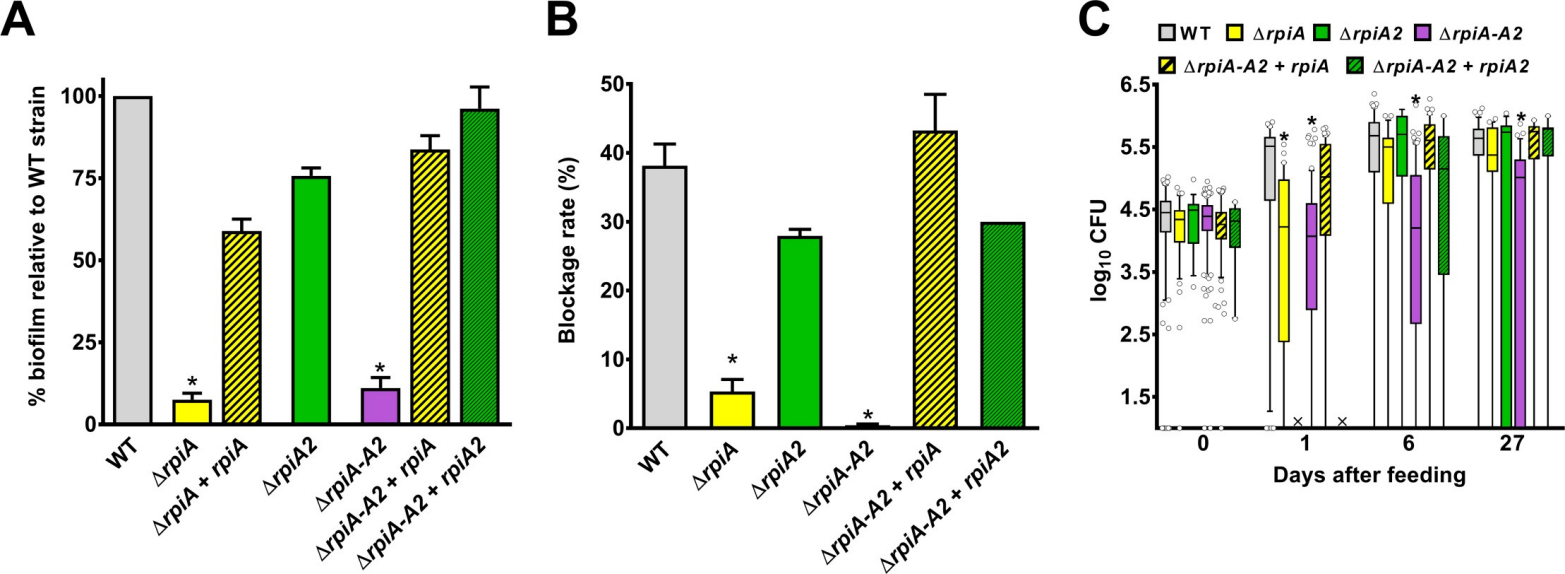
RpiA and RpiA2 are redundant in the production of a transmissible infection in fleas. We measured the ability of Δ*rpiA* (in yellow), Δ*rpiA*2 (in green) and Δ*rpiA* Δ*rpiA*2 (Δ*rpiA-A2; in* violet) mutants complemented or not with a high-copy-number plasmid harboring *rpiA* (yellow hatches) or *rpiA*2 (green hatches) to (A) form biofilms *in vitro*, (B) block fleas and (C) colonize fleas, relative to the WT strain (in grey). (A) The bars represent the mean ± SEM value from at least three independent experiments, except for the Δ*rpiA* Δ*rpiA*2 mutant complemented with *rpiA*2 (two experiments only). The Δ*rpiA* and the Δ*rpiA* Δ*rpiA*2 mutants produced significantly less biofilm than the WT and the complemented strains (*: p<0.01 in a one-way analysis of variance). (B) The bars represent the mean ± SEM from three independent experiments (Δ*rpiA* and Δ*rpiA* Δ*rpiA*2 mutants), two independent experiments (Δ*rpiA*2 and Δ*rpiA* Δ*rpiA*2 + *rpiA*) or one experiment (Δ*rpiA* Δ*rpiA*2 + *rpiA*2 mutant). The Δ*rpiA* and Δ*rpiA* Δ*rpiA*2 mutants blocked significantly fewer fleas than the WT strain (*: p<0.01 in a one-way analysis of variance). (C) Box-and-whisker plots (5–95% percentiles) show the bacterial loads determined from up to 20 fleas in each experiment (i.e. a total of 50 to 259 fleas). The result of one experiment (Δ*rpiA*2 D6 and Δ*rpiA* Δ*rpiA*2 + *rpiA*2 at all time points) and the cumulative results of two experiments (Δ*rpiA* D27) and ≥ three experiments (all other strains and time points) are shown. Symbols indicate outliers. X: not determined. The bacterial loads for the Δ*rpiA* strain were significantly lower than for the WT strain at D1, whereas those for the Δ*rpiA* Δ*rpiA*2 strain were significantly lower throughout the experiment (*: p<0.01 in a one-way analysis of variance). The Δ*rpiA*2 mutant and the complemented mutants behaved like the WT strain.

To gain a better idea of how the disease progresses in fleas, we next sought to identify new genes involved in the mechanisms leading to flea blockage. To this end, we took advantage of a comparative transcriptomic analysis that had highlighted 463 *Y*. *pestis* genes as being transcriptionally activated in the flea gut and thus perhaps of importance in transmission [4]. We therefore generated a library of 175 *Y*. *pestis* mutants lacking one or more of these activated genes; in order to reduce the number of mutants to be evaluated and therefore the number of animals to be used to feed the fleas, we deleted blocks of neighboring genes (regardless of their genetic organization and relationships) whenever possible. Next, we screened it for *in vitro* biofilm formation in four different media; this identified mutants for high-priority testing in the flea, since biofilm formation is needed for blockage. Twenty-four of the mutants had an abnormal phenotype, and 9 of these had an abnormal phenotype under at least two different growth conditions (Fig 4). Subsequent studies (using new, independent and/or complemented mutants) of these 9 mutants indicated that the genes coding for DNA helicase Rep, glycerol-3-phosphate oxidase (GlpD), ribose-5 phosphate isomerase (RpiA), the transcription regulator HdfR, an adhesin (Ail), and (presumably) the Rsx reducing complex are required for biofilm formation *in vitro* and thus (perhaps) flea blockage *in vivo* (Figs 5 and 6A). However, only the Δ*rpiA* mutant blocked a significantly smaller number of fleas than the WT strain (albeit in a stochastic way) over a 4-week period (Figs 6B, S4 and S5). Consistently, the flea mortality rate (which is correlated with flea blockage rate [7, 22]) over a 4-week period was lower in a cohort of 80 female fleas infected with the Δ*rpiA* mutant (6.3% ± 2.5%; n = 3) than in a cohort of female fleas infected with the wild-type strain (25.6% ± 5.3%; n = 4). The mutant’s impairment in blockage did not result from poor ability to establishing an infection, since the percentage of fleas infected with the mutant and with the WT were similar at 1 and 4 weeks post-infection (S1 Table). A bacteriological analysis showed that the mutant was present in lower amounts than the parental strain the day after infection (Fig 6C). However, this anomaly was no longer observed on day 6 post-infection (Fig 6C). Thus, the inability of the Δ*rpiA* mutant to efficiently block the flea might result from poor colonization of the proventriculus.

### The *rpiA* gene is required for resistance to the bactericidal proventricular mass

**Fig 7:**
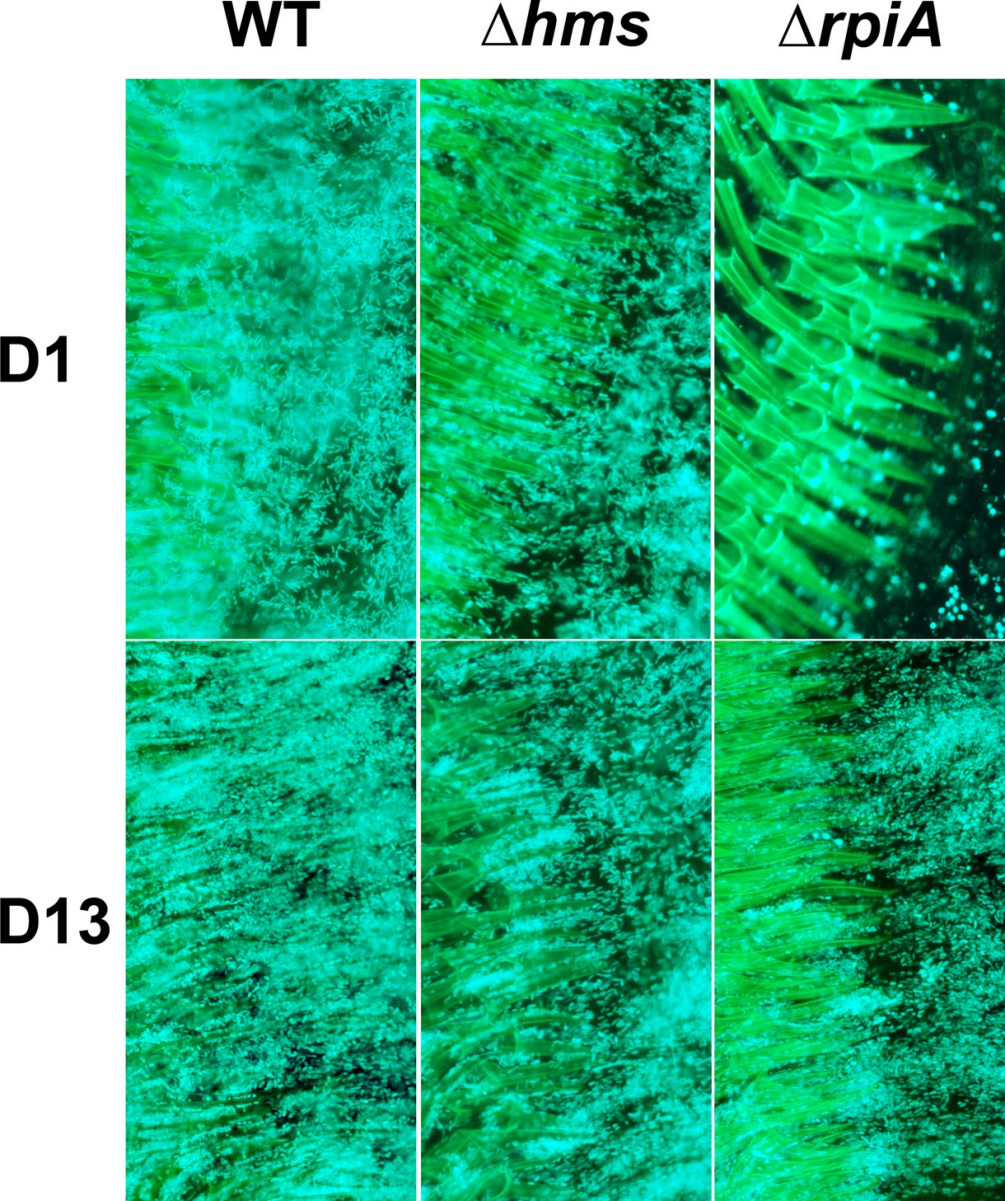
*rpiA* (but not *hms*) is needed to maintain a normal bacillary shape during the early (but not late) colonization of the mass anchored within the proventriculus. Fluorescence microscopy images show the presence of WT, Δ*hms* or Δ*rpiA* bacteria (in blue) in the brownish mass anchored within the proventriculus (PV, green spines) on day (D)1 and D13 post-infection. Images were post-processed using the curve adjustment tool in Adobe Photoshop, in order to highlight the bacteria (in blue) and the PV’s spines (in green).

To investigate the early post-infection role of *rpiA* in fleas, we used bright-field and fluorescence microscopy techniques to compare the gut contents of insects one day after a meal containing fluorescent WT or Δ*rpiA* bacteria. For both strains, ~70% of the fleas had a brownish mass anchored within their proventriculus (as for the Δ*hmsHFRS* mutant and in our above-mentioned preliminary study) (Fig 1). These masses contained a large number of WT and Δ*hmsHFRS* bacilli but very few Δ*rpiA* mutants, which are mostly coccoids (Fig 7 [D1]). The phenotype of the Δ*rpiA* mutant (which has not been described for any mutants evaluated in fleas so far) suggests that the mass initially produced in the foregut (i) is not a bacterial biofilm produced by *Y*. *pestis*, (ii) is bactericidal, and (iii) might be part of a flea’s immune response to the foregut infection by *Y*. *pestis*. As mentioned above, it should be borne in mind that *ymt* is needed for production of the proventricular mass. Taken as a whole, the data suggests that the flea’s immune response to *Y*. *pestis* ingested in blood results in the formation of a bacteria-entrapping mass in the foregut and/or the mass contains a toxic compound resulting from blood digestion. The data also suggest that the recent acquisition of *ymt* by *Y*. *pestis* is essential for the induction of a proventricular mass, whereas the most ancestral bacterial gene *rpiA* (found in the ancestral species, and in contrast to *ymt*, which was acquired during the emergence of *Y*. *pestis*) is required for resistance to the mass produced as part of the flea’s immune response.

### *rpiA* is needed for resistance to antimicrobial peptides under conditions that mimic early steps in flea infection

In contrast to the midgut, the proventriculus is cuticularized; it is noteworthy that cuticularized insect epithelium secretes antimicrobial peptides upon exposure to bacteria, which could explain the Δ*rpiA* mutant’s phenotype described above [25]. Interestingly, we found that the Δ*rpiA Y*. *pestis* mutant was more sensitive than the WT to polymyxin B under conditions mimicking the passage from the mammal to the flea vector (i.e. the early steps in flea infection). When bacteria cultured at 37°C were brought into contact with polymyxin B at 21°C, the mean ± standard deviation (SD) minimum inhibitory concentration (MIC) was 11.25 ± 0, 4.7 ± 1.6, and 11.25 ± 0 μg/mL for the WT, the Δ*rpiA* mutant and the complemented Δ*rpiA* mutant, respectively (n = 3 independent experiments). This high sensitivity to polymyxin B was not due to a truncated lipo-oligosaccharide, as one might expect (S6 Fig) given that RpiA synthesizes ribulose-5P, which is converted into the lipo-oligosaccharide precursor sedoheptulose 7-phosphate [26]. Regardless of the exact role of RpiA in antimicrobial peptide resistance, our data suggest that optimal colonization of the amorphous bactericidal mass produced in the proventriculus requires this enzyme—presumably because it would confer resistance to harmful compounds such as antimicrobial peptides.

### *rpiA* is likely to consolidate the amorphous soft mass produced by the flea in the foregut

**Fig 8:**
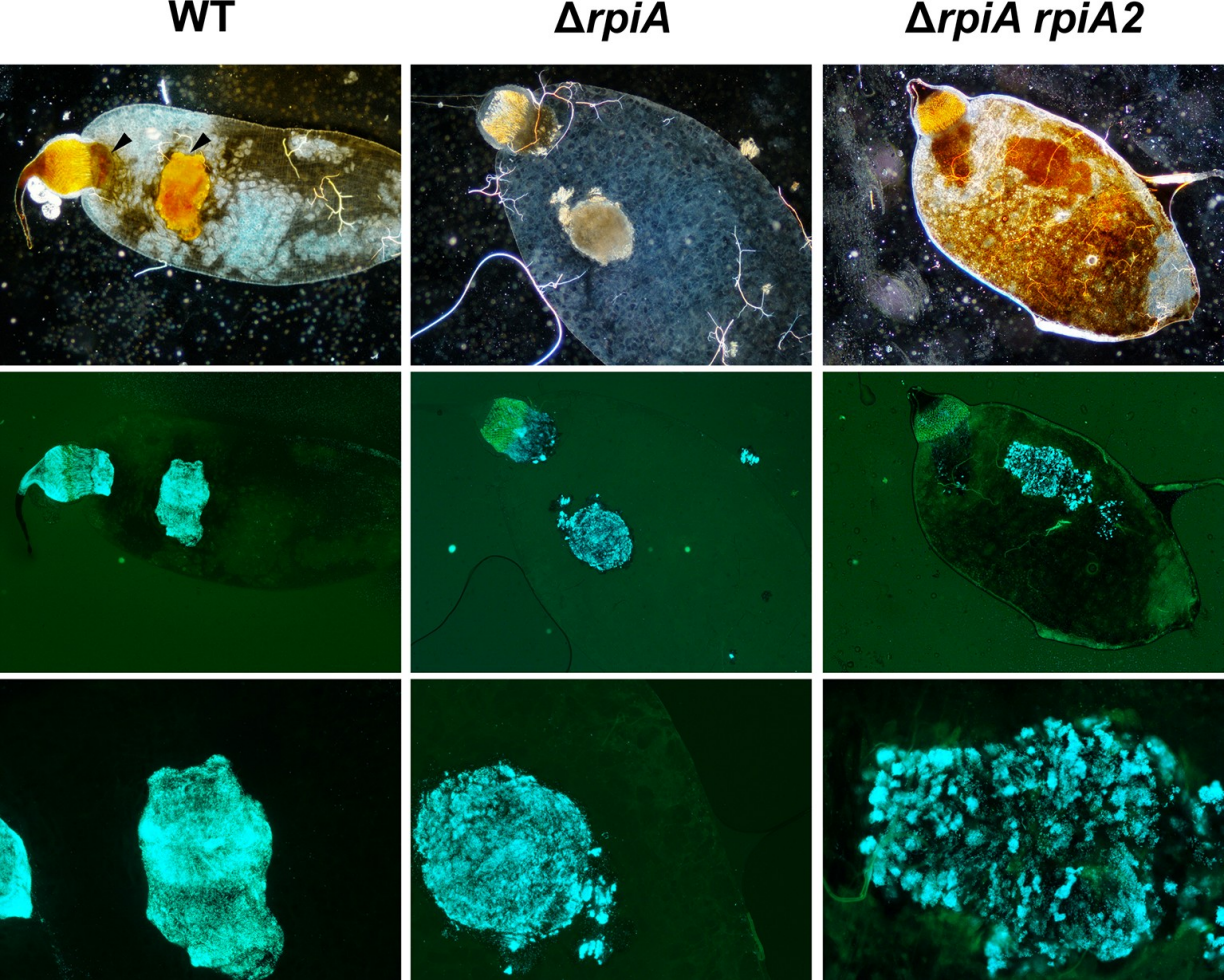
The proventricular casts contain WT, Δ*rpiA* and Δ*rpiA* Δ*rpiA2 Y*. *pestis*. Images of the flea guts and proventricular casts were acquired using a bright-field microscope (top) and a fluorescence microscope (center and bottom). The fluorescence images were modified (using Photoshop) to highlight the bacteria in blue. The proventriculus autofluoresces in green.

**Fig 9:**
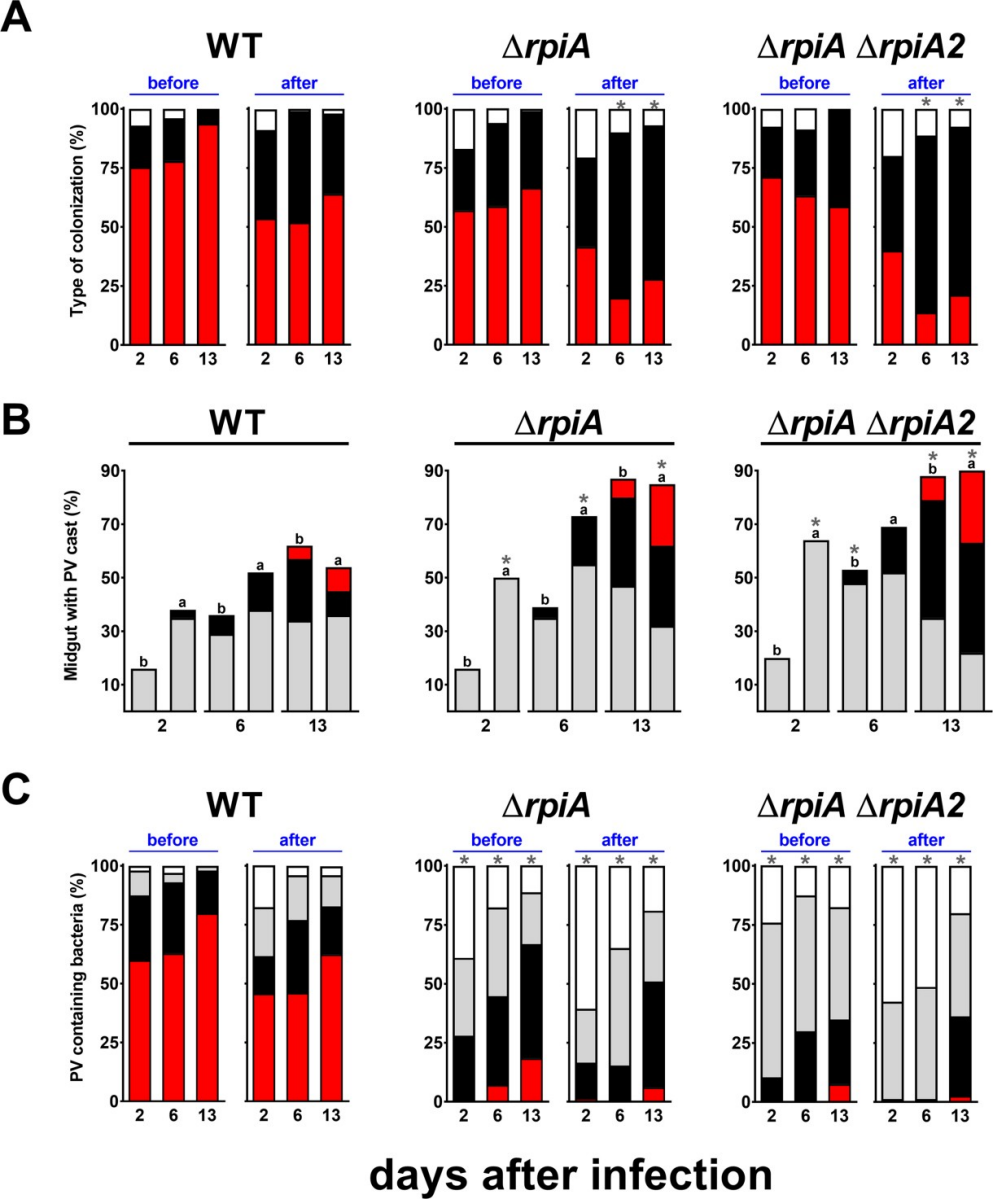
RpiA activity is needed for the production of a thick *Y*. *pestis* biofilm that blocks the flea proventriculus. (A) the percentage of fleas showing no masses (white), a brownish mass anchored within the proventriculus (red), or only a free-floating mass within the midgut (black) [see Fig 1], (B) the percentage of fleas containing one (grey), two (black) or more than 2 (red) proventricular casts in the midgut (regardless of the presence of a mass associated with the proventriculus), and (C) proventriculi containing no (white), very few (grey), few (black) or many (red) bacteria were determined at 2, 6 and 13 days post-infection, before (b) and immediately after (a) feeding. The stacked data from 4 independent experiments (the Δ*rpiA* Δ*rpiA2* mutant and WT, D13, after feeding) and 5 independent experiments (WT and Δ*rpiA* mutant) using >15 to 20 fleas are presented (see S7 Fig). *, p <0.05 using 2-way analysis of variance with Tukey's multiple comparisons test.

Three observations suggested that resistance to antibacterial compounds might partly account for the Δ*rpia* mutant’s impaired ability to block the gut. Firstly, the Δ*rpiA* and WT strains were equally resistant to polymyxin B under conditions mimicking an established infection, i.e. when bacteria were grown at 21°C and then brought into contact with polymyxin B at 21°C (mean ± SD MIC = 11.25±0 μg/mL for all strains; n = 3 independent experiments). Secondly, the mutant had recovered its bacillary shape on days 6 and 13 post-infection (Fig 7). Thirdly, the midgut of fleas infected with the mutant contained free floating proventricular cast containing a large number of bacteria (Fig 8). Fourthly, the mutant did not heavily colonize the proventriculus in most fleas before 13 days post-infection but did colonize the midgut (to the same extent as the WT) during the period when blockage is normally observed (Figs 6C and S7 [images acquired before feeding]). Hence, it is possible that once *Y*. *pestis* has adapted to the flea vector, it might use RpiA to consolidate the mass produced in the proventriculus (i.e. by forming a biofilm), so that the mass resists the shear forces associated with the blood flow and rhythmic contractions of the proventriculus during feeding. This is why we next compared proventricular clearance after feeding in fleas infected with the WT or the mutant strain. Notably, we looked at (i) the whole gut content of fleas infected with the WT or the mutant strain (to determine the presence of a proventricular cast attached to the proventriculus and/or within the midgut), and (ii) the two strains’ ability to maintain themselves in the proventriculus before and immediately after fleas had fed at different time intervals post-infection. Our bright-field microscopy results showed that 70% of the fleas infected with WT or Δ*rpiA* strains had a brownish mass (a cast) anchored to their proventriculus. Furthermore, regardless of the presence of a mass within the proventriculus, we observed the presence of one, two or more free-floating brownish casts in the midgut (as shown in Fig 1). For both strains, the proportion of infected fleas with a mass anchored within the proventriculus decreased after each feed (Fig 9A, red bars, before *vs*. after). Conversely, for both strains, the proportion of fleas displaying a “clear” proventriculus plus a proventricular cast in the midgut increased after feeding (Fig 9A, black bars, before *vs*. after). Likewise, for both strains, we observed that the proportion of fleas with a midgut containing one or several free-floating casts increased over time (Fig 9B). Thus, feeding displaced the mass located in the proventriculus into the midgut for both strains. We also observed that before feeding, the proportion of fleas with an obstructed proventriculus increased over time for the WT strain only (Fig 9A, red bars before feeding); for the mutant strain, the proportion of fleas with a "clear" proventriculus plus a proventricular cast in the midgut increased (Fig 9A, black bars before feeding). Thus, feeding seemed to displace the mass located in the proventriculus more easily when the flea was infected with the Δ*rpiA* mutant. This idea is consistent with the fact that the proportion of fleas accumulating more than one cast in the midgut was higher with the mutant strain (Fig 9B).

**Fig 10:**
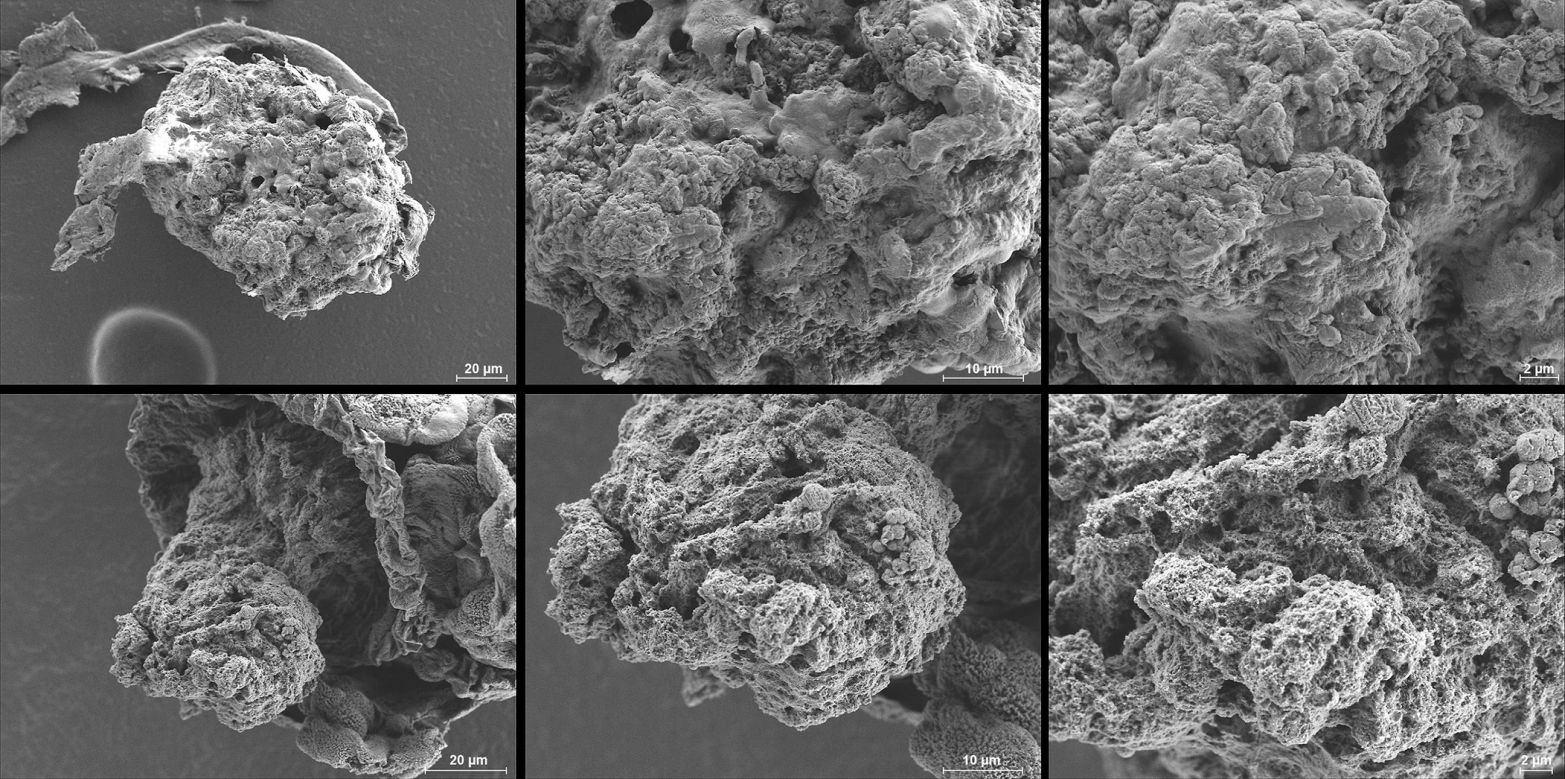
RpiA activity is needed for the production of a thick mass in the flea gut. A scanning electron micrograph of the biofilms produced by the WT (upper photos) and the Δ*rpiA* mutant (lower photos) in the flea at days 13 post-infection and taken at different magnifications.

The proventricular clearance of fluorescent bacteria was similar to the clearance of the proventricular mass (Fig 9C). However, marked clearance of fluorescent bacteria was observed only with the mutant—even 13 days post-infection, when the proventriculus contained substantial numbers of bacteria (Figs 9C and S7). Taken as a whole, the results of our bright-field and fluorescence microscopy experiments confirmed the bacteriologic analysis and suggested that the biofilm generated by the *ΔrpiA* mutant is more likely to be flushed out by blood entering the flea’s gut during feeding—presumably because the biofilm’s structure is too loose. Similarly, our comparative analysis of the mass attached to the proventriculus (based on electron microscopy) showed that (i) WT bacteria were buried in a structure with the appearance of "smooth concrete", and (ii) mutant bacteria were enclosed in a rough and/or spongy, open structure that might conceivably be more easily engulfed and destroyed by blood flow (i.e. suction pressure) during a meal (Figs 10 and S8). Hence, RpiA is needed to consolidate the mass that is initially anchored within the proventriculus–presumably because this enzyme is involved in the formation of the thick biofilm that occludes the flea's gut.

### *Y*. *pestis* encodes a second RpiA enzyme (RpiA2) that maximizes the blockage rate

Although RpiA is important for *in vitro* biofilm formation and *in vivo* gut blockage, it is not essential. This suggests the presence of a redundant enzyme involved in the same processes. Indeed, the Kyoto Encyclopedia of Genes and Genomes database suggests that *Y*. *pestis*’ *y2892* gene encodes a second putative ribose-5 phosphate isomerase (hereafter referred to as RpiA2) that shares 33.7% identity with RpiA. We found that RpiA2 can indeed convert ribose-5-phosphate (R5P) to ribulose-5-phosphate (Ru5P). However, RpiA2’s role was only obvious in fleas and in a Δ*rpiA* genetic background (i.e. with a Δ*rpiA* Δ*rpiA2* mutant) (Fig 6). In contrast to the single Δ*rpiA* mutant, the double Δ*rpiA* Δ*rpiA2* mutant was unable to block fleas (Fig 6B), and it colonized the flea gut less heavily at all time points (Fig 6C). However, more than 70% of the fleas were still infected 1 and 27 days post-infection. Furthermore, more than 50% of the fleas were still heavily colonized (>10^4^ CFUs) one week (96 out of 178 fleas) and four weeks (51 out of 78 fleas) after the infected meal. This phenotype is similar to that of an Δ*hms* mutant, which is unable to block fleas [7]. Thus, the failure of the double Δ*rpiA* Δ*rpiA2* mutant to block fleas might be due to its inability to generate a biofilm, rather than its inability to colonize the flea’s gut. Bright-field and fluorescence microscopy experiments (performed in the same way as for the Δ*rpiA* mutant) indicated that *rpiA2* deletion exacerbates the *rpiA* mutant’s phenotype (Figs 9 and S7). Notably, there were fewer bacteria in the proventriculus (Figs 9C and S7). Thus, both RipA2 and RpiA are required to colonize the proventriculus (but not the midgut), and are involved in the formation of the mature biofilm needed to block the flea.

### The quantity of RpiA enzymes correlates with the blockage rate

A previous transcriptional analysis had indicated that *rpiA* is more highly expressed in fleas than *rpiA2* [4]. Accordingly, we found that *rpiA* and *rpiA2* accounted for respectively ~85% and ~15% of the blockages. Our data suggest that high levels of *rpiA* expression and (presumably) R5P or Ru5P production (i.e. a high metabolic flux through an RpiA-dependent reaction) increase the blockage rate and thus the likelihood of plague transmission. This idea was supported by the observation that over-expression of *rpiA2* (under the control of the *P*_lac_ promoter in a high-copy-number plasmid) restored the flea blockage and colonization associated with the Δ*rpiA* Δ*rpiA2* mutant to WT values (Fig 6B). To further establish whether the flea blockage rate increased with the metabolic flux through the RpiA-catalyzed reaction, we generated and tested *Y*. *pestis* mutants expressing only a single *rpiA* gene: either *rpiA* under the control of the *rpiA2* promoter (i.e. *rpiA* had been moved to *rpiA2*’s location) or *rpiA2* under the control of the *rpiA* promoter (i.e. *rpiA2* had been moved to *rpiA*’s location). We found that the strain expressing only *rpiA2* under the control of *rpiA promoter* blocked fleas to the same extent as the WT (43% *vs*. 49%, respectively) whereas the strain expressing only *rpiA* under the control of the *rpiA2* promoter blocked only 2% of the fleas. Overall, the data suggest that (i) the *rpiA* expression level and (presumably) a high metabolic flux for Ru5P or R5P conversion are correlated with the blockage rate, and (ii) increased metabolic flux (via the acquisition of *rpiA2* by *Y*. *pestis* from *Y*. *pseudotuberculosis*) increased the potential for the epidemic spread of plague.

### The metabolic connection between glycolysis and the pentose phosphate pathway (but not the xylulose, arabinose or gluconate pathway) is needed to block fleas

**Fig 11:**
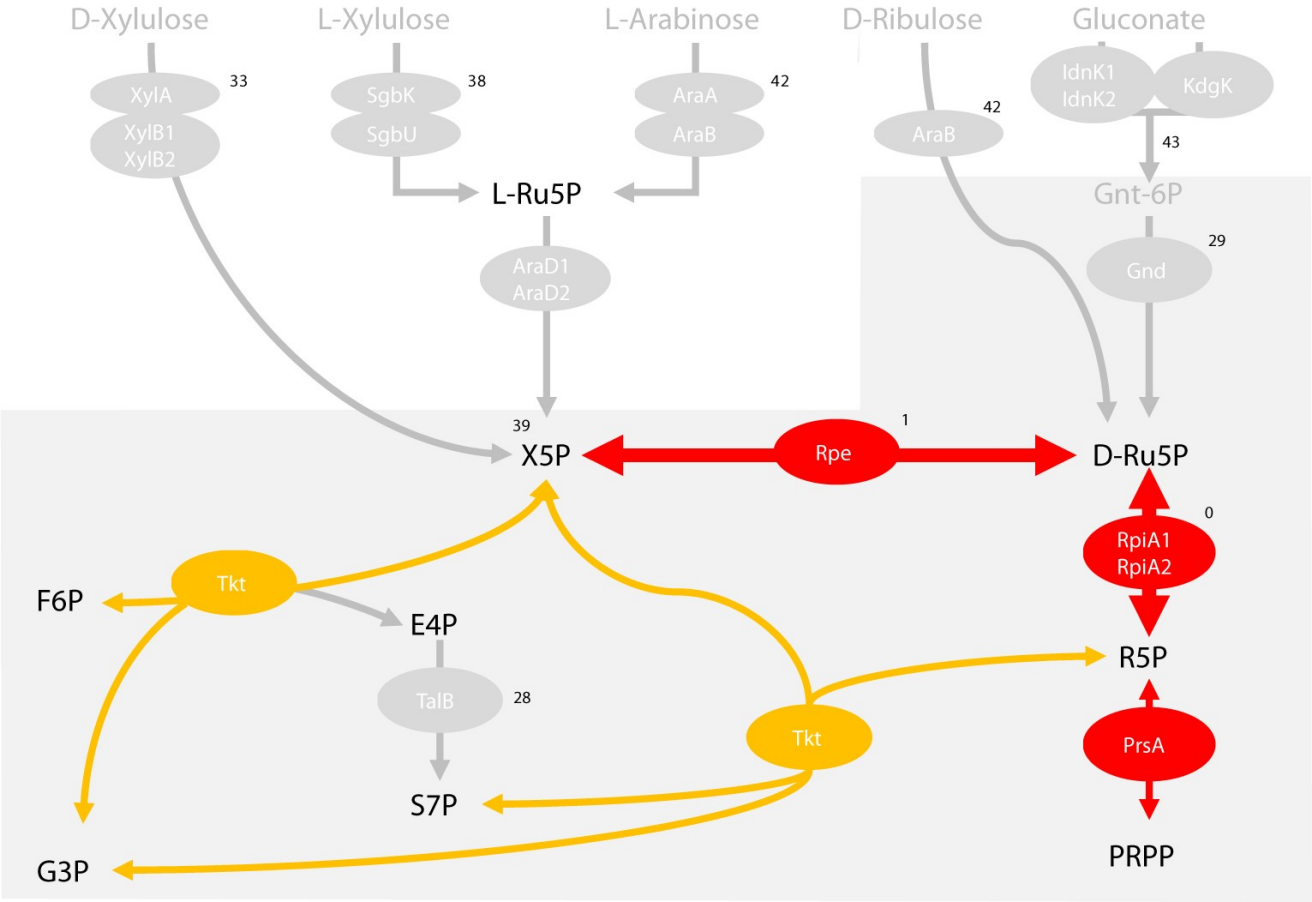
Pentose-related metabolic pathways that might have a role in flea blockage. The figure shows the flea blockage rate for mutants lacking D-xylulose (Δ*xylAB1*), L-xylulose (Δ*sgbK-sgbU*), arabinose/D-ribulose (Δ*araAB*), D- and L-xylulose and arabinose/D-ribulose (Δ*araD1* Δ*araD2* Δ*xylB1* Δ*xylB2*) pathways with or without Rpe (Δ*araD1* Δ*araD2* Δ*xylB1* Δ*xylB2* Δ*rpe*), gluconate import (Δ*idnK1* Δ*indk2* Δ*kdgK*) or use via the upper part of the pentose phosphate pathway (Δ*gnd*), Δ*talB*, Δ*rpe* or Δ*rpiA* Δ*rpiA2*). Grey arrows indicate pathways that are not important for flea blockage. The other reactions are considered to be important for flea blockage because the mutant either (i) does not block fleas (Δ*rpe* and Δ*rpiA* Δ*rpiA2* mutants), (ii) was highly deficient for growth *in vitro* (Δ*tktA)* (see supplementary text) or (iii) could not be deleted (Δ*prsA*), presumably because it is essential (as reported for *E*. *coli*). The grey area encloses the enzyme reactions of the pentose phosphate pathway.

Lastly, we sought to determine the source and potential fates of RpiA’s substrates (Ru5P and R5P). We reasoned that these potentially harmful metabolites might be actively consumed so as to avoid accumulation. Indeed, the metabolites D- and L-xylulose, L-arabinose, D-ribulose, gluconate, and sedoheptulose-7-phosphate (S7P) can be converted into Ru5P (Fig 11). Like phospho-alpha-D-ribosyl-1-pyrophosphate (PRPP), S7P is also a source of R5P. However, all the other enzymatic reactions–with the exception of the conversion of gluconate into Ru5P –are reversible. It is noteworthy that according to the KEGG database, the production of xylulose-5-phosphate from D- and L-xylulose or arabinose involves redundant enzymes. The phosphorylation of D-xylulose into xylulose-5-phosphate may involve the xylulose kinases XylB1 and XylB2, whereas L-Ru5P (produced from the L-xylulose or the L-arabinose) could be converted by the L-ribulose-5-phosphate 4-epimerases AraD1 and AraD2. Gluconate could be converted into gluconate-6-phosphate by the gluconokinases IdnK1 and -K2 or produced by the 2-dehydro-3-deoxygluconokinase KdgK then into Ru5P following decarboxylation of the gluconate-6-phosphate by the 6-phosphogluconate dehydrogenase (Gnd). Hence, we generated a battery of mutants lacking one or several metabolic pathways upstream or downstream of these compounds, and evaluated their respective roles in flea blockage to determine the sources of Ru5P and R5P and establish whether the absence of RpiA leads to the accumulation of harmful metabolites. However, the role of PRPP was not evaluated because the ribose phosphate pyrophosphokinase that reversibly converts PRPP into R5P is essential for bacterial growth–at least *in vitro*. Otherwise, we found that D/L-xylulose, L-arabinose, D-ribulose and gluconate are not major sources of Ru5P, and that accumulation of the corresponding substrates is not toxic *in vivo*. Indeed, the deletion of pathways converting D/L-xylulose and L-arabinose into xylulose-5P and pathways converting D-ribulose and gluconate intro R5P had no impact on *Y*. *pestis*’ ability to block the flea (Figs 11 and S9). In contrast, the reversible conversions of S7P into Ru5P and R5P and of xylulose-5-phosphate into Ru5P (catalyzed respectively by the transketolase Tkt and the ribulose-5-phosphate 3-epimerase Rpe) are required for flea blockage. The Δ*tktA* mutant grew extremely slowly *in vitro*, and the Δ*rpe* strain blocked only 1% of fleas (in two independent experiments)–even though it was able to colonize the latter to WT levels (median (range): 5.51 log_10_ CFUs (5.01 to 5.78)). Lastly, microscopy experiments showed that the *rpe* mutant was coccoid soon after infection but not later on—suggesting that Rpe is involved in the same functions as RpiA in fleas. Surprising, our data also indicate that *Y*. *pestis* does not use several pentose and gluconate metabolic pathways–despite the fact that the bacterium has many redundant enzymes.

## Discussion

Around 15 years after Simond had proved that fleas are plague vectors, Bacot reported that total or partial blockage of the flea’s foregut is an important biological process in the transmission of plague by fleas [6, 9, 27]. In particular, Bacot proposed that the development of a *Y*. *pestis*–containing mass in the proventriculus leads to the reflux of blood contaminated by bacilli at the flea-bite site. It was subsequently suggested that the colonization of the midgut preceded that of the proventriculus [28]. Consistently, it was recently reported that *Y*. *pestis* forms aggregates with blood-derived components in the midgut within a few hours of ingestion, and is then drawn into the proventriculus by antiperistalsis and proventricular pulsation [21]. The initial mass of the proventriculus was thought to be poorly adherent and thus rapidly discharged into the midgut by incoming blood (at least when *X*. *cheopis* was fed on mouse blood). Hence, the dislodged bacterial aggregates would recolonize the proventriculus, where *Y*. *pestis* forms a compact mass that subsequently blocks the proventriculus [21].

**Fig 12:**
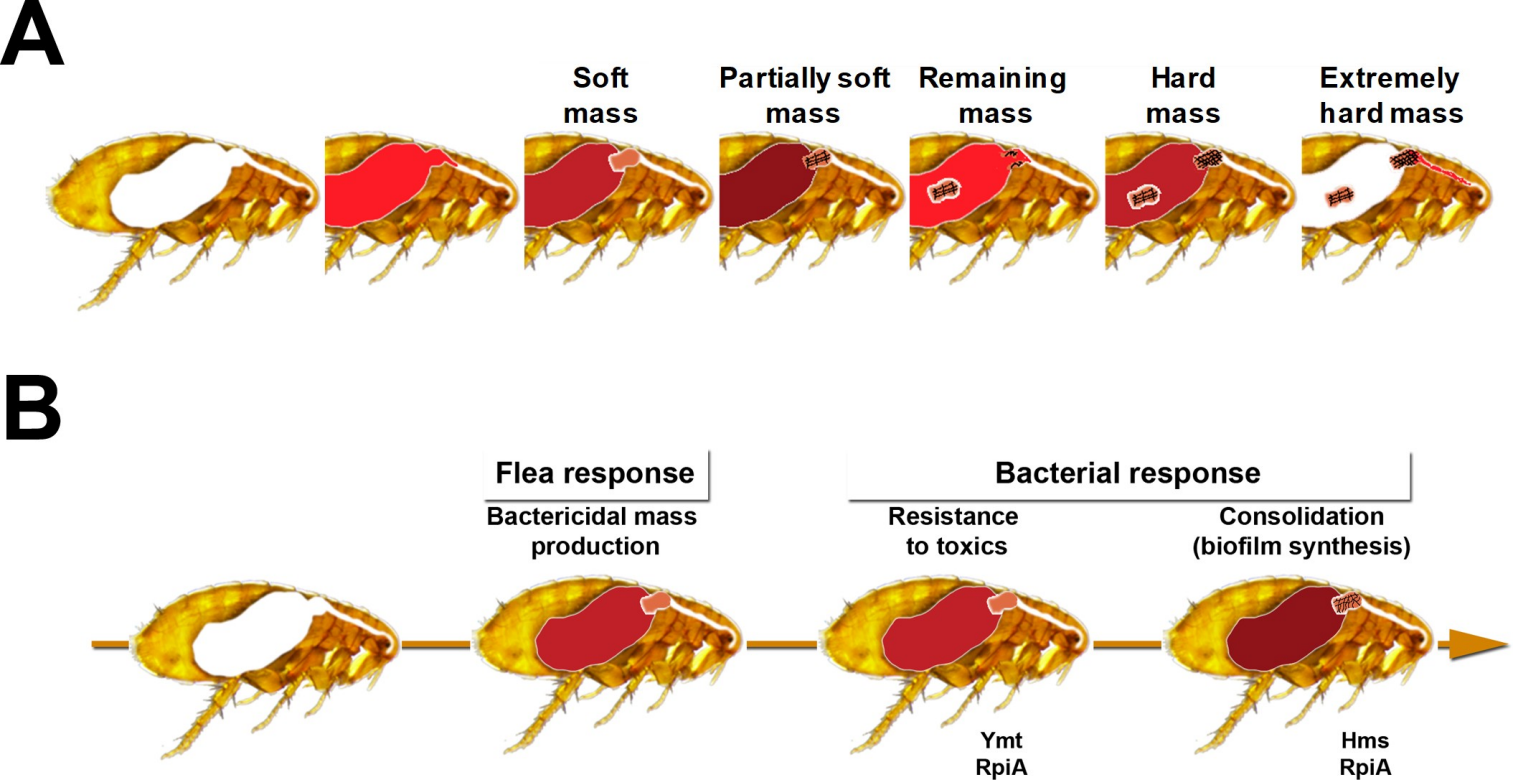
A physical and molecular model of the processes leading to flea blockage by *Y*. *pestis*. (A) Blockage of the flea’s foregut results from recurrent production of a soft bactericidal proventricular mass (a cast), and then the repeated colonization and [partial] decolonization of the proventriculus and the gradual consolidation of the cast until it is strong enough to withstand the incoming blood flow. The occasional eviction of the mass by the flea itself (i.e. without the need to ingest blood) is not shown. (B) The induction of, resistance against and consolidation of the soft bactericidal proventricular cast (the flea’s response to infection) respectively involve the phospholipase D Ymt, the ribose phosphate isomerases A and A2, and the biofilm synthesis complex HmsHFRS. It should be noted that following *Y*. *pestis*' entry into the flea, RpiA is initially required to resist toxic compounds within the proventricular cast. Once the bacterium has adapted to its new host, the enzyme is no longer required for toxicity resistance but is then needed to produce the biofilm that consolidates the proventricular cast.

Based on our microscopy results, we developed a refined physical model of flea-borne plague in *X*. *cheopis* fed on mouse blood (Fig 12). In this model, a gelatinous soft bactericidal mass (a cast) containing *Y*. *pestis* and filling the whole lumen of the proventriculus is produced within one hour of infection. Next, the soft cast and the bacteria entrapped therein are almost completely flushed into the midgut, and a new cast in which *Y*. *pestis* grows develops in the proventriculus (Figs 1, 3, 8, 9 and S7). Although the cast is usually dislodged during a blood meal, it is possible that the growing cast is occasionally dislodged by the flea itself—probably due to pulsations of the proventriculus (Table 1). This process of proventricular cast production, colonization and partial decolonization continues until a cast is enough strong to withstand an incoming blood flow, and so blocks the flea. Hence, our model suggests that midgut colonization may not be essential for colonization and blockage of the proventriculus. It is noteworthy that soon after infection (but also at later time points), the anterior and posterior halves of the proventriculus are both colonized but are separated by a “green buffer zone” that is free of bacteria or contains only a few bacteria (Fig 2). This striking, three-zone colonization of the proventriculus may correspond to the differing putative roles of the anterior and posterior spine-bearing regions of the proventricular valve: the former (which contains blood after the blood meal) serves as an effective barrier to blood regurgitation, whereas the latter is involved in blood cell disruption [29]. Hence, as well as proposing that the proventriculus is the primary and sole site of infection leading to flea blockage, we further suggest that the anterior half spine-bearing region of the proventriculus is the initial site of infection leading to the recurrent colonization and, ultimately, blockage of the proventriculus.

Our model may challenge the long-held idea whereby the colonization of the midgut precedes that of the proventriculus [28, 21]. Furthermore, our model does not support the recent assumption whereby peristalsis and proventricular pulsations draw *Y*. *pestis* aggregates back into the proventriculus from the midgut (i) very soon after infection or (ii) after the poorly adherent bacterial aggregate formed by the initial backflush is dislodged by incoming blood [21]. If the production of an early proventricular soft mass indeed resulted from a backflush of bacteria via on antiperistaltic and proventricular contractions, it would not be possible to observe a cast containing a small number of individual bacteria anchored in the proventriculus, as is the case soon after infection with *Y*. *pestis* lacking *rpiA* or *rpe* (Fig 7). We cannot rule out the possibility that antiperistalsis and proventricular pulsations forcibly thrust *Y*. *pestis* from the midgut into the proventriculus. However, this event may only be possible during a very short period of time, since the proventriculus was usually obstructed with an hour of infection (Table 1). In fact, the three-zone colonization of the proventriculus (Fig 2) suggests that antiperistaltic movements could push some bacteria back from the midgut into the lower part (at least) of the posterior region of the proventriculus. However, some bacteria might also occasionally reach the anterior region as a result of antiperistaltic movements. However, this possibility could be stochastic despite the presence of a central circular canal between the spines. Indeed, it has been suggested that the anterior spine-bearing region prevents regurgitation and the diameter of the central circular canal is not very large (63 μm and 85 μm in male and female fleas, respectively), relative to the length of *Y*. *pestis* (2 μm) to allow the backflow of the large number of bacteria observed in the anterior half of the proventriculus [29, 30]. Lastly, one can imagine that the last drops of blood ingested into the anterior region of the proventriculus may be trapped until the flea starts to defecate; the central circular canal may have a role in this process. Hence, one can reasonably hypothesize that bacteria trapped in the proventriculus colonizes the anterior spine-bearing part of the proventriculus at the end of the blood ingestion, whereas the colonization of the posterior part of the proventriculus results from antiperistaltic movements that push bacteria out of the midgut. In this model, both events are required to produce the full proventricular cast and thus impairment of the proventriculus’ function.

Regardless where the bacteria located in the anterior and posterior halves of the proventriculus come from, it is clear that the infection of this structure is associated with the formation of a soft, bactericidal, brownish proventricular cast as soon as one hour after infection. This cast is not a conventional *hmsHRS*-dependent bacterial biofilm. A previous attempt to characterize the flea-blocking, *Y*. *pestis*-containing matrix highlighted the presence of as-yet uncharacterized lipids, other products derived from the blood meal, and hemin from red blood cells—explaining the cast’s brownish color [31]. Just like the composition of the matrix produced soon after infection, the triggering organism (*Y*. *pestis* and/or the flea) is also subject to debate. *Y*. *pestis* (rather than the flea) might be involved because the intracellular phospholipase D Ymt produced by the bacterium is a key intracellular enzyme in the production of the mass. However, it is not clear what Ymt’s direct role would be, the enzyme would have to induce the formation of a mass in a region that lacks bacteria or contains few bacteria (the green buffer zone, for instance). Hence, Ymt is more likely to have an indirect role in the production of the mass–perhaps by protecting *Y*. *pestis* against a cytotoxic product present in the flea gut [2]. In other words, the colonization of the flea foregut (thanks to Ymt) might induce a strong immune response by the proventriculus so as contain and destroy the infectious agent–perhaps through the production of a peritrophic-like membrane. This idea is compatible with the proventriculus’ reported role in the tsetse fly. Indeed, the gut’s “gatekeeper” has a dual role in host protection by synthesizing peritrophic matrix constituents and by expressing immune effector molecules (including antimicrobial peptides, and reactive oxygen and nitrogen species) [32]. If a “Ymt-induced excessive immune response” indeed occurs, it would mean that *Y*. *pestis* induces and then exploits an immune response to produce an effective, transmissible infection in fleas. We intend to investigate this putative mechanism in the near future.

From a molecular point of view, our data indicate that the induction, resistance and consolidation of the soft bactericidal proventricular cast respectively involve the phospholipase D Ymt, the ancestral ribose phosphate isomerase A, and the biofilm synthesis complex HmsHFRS. As mentioned above, Ymt may have a direct or indirect role in the induction of the mass, since this enzyme is essential for resisting a cytotoxic blood plasma digestion product in the flea gut [2]. It appears that RpiA is also required for resistance to various toxic compounds encountered in the flea gut. However, our data suggest that once the bacterium has adapted to its new host, RpiA is no longer required for toxicity resistance but is needed to produce the biofilm that consolidates the proventricular mass. Hence, RpiA’s role in flea blockage relies on two distinct functions—making it the first gene of this kind among all the genes known to be involved in flea infection. RpiA’s exact roles will now have to be investigated.

**Fig 13:**
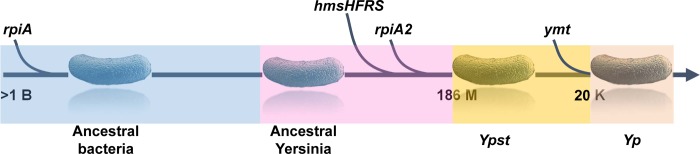
Slow, gradual genetic accretion punctuated by sudden jumps led to the emergence of plague transmission via the flea vector.

Although RpiA is important for flea blockage, a *Y*. *pestis* mutant lacking *rpiA* can still block a fair proportion of fleas (up to ~30%, relative to the WT). This is because *Y*. *pestis* has a second *rpiA* gene (*rpiA2* or *y2892*). Interestingly, *rpiA2* is present in only one of the 18 *Yersinia* species (i.e. the *Y*. *pseudotuberculosis* complex from which *Y*. *pestis* recently emerged) -suggesting that *rpiA2* was acquired horizontally by *Y*. *pseudotuberculosis*. More intriguingly, *rpiA2* is also present in *Erwinia typographi*; the latter was recently isolated from the gut of a phytophagous beetle [33]. We cannot speculate as to the exact origin of *rpiA2*. Regardless of its origin and function(s) in *Y*. *pseudotuberculosis*, our data reveal that *rpiA2* is the second locus (after *hms* [7]) likely to have been acquired horizontally in *Y*. *pseudotuberculosis* and that has a role in flea blockage by *Y*. *pestis*. Given that *rpiA* is an ancestral bacterial gene and that *ymt* was acquired by *Y*. *pseudotuberculosis* during the emergence of *Y*. *pestis* [2], our data indicate that the emergence of flea blockage was made possible when (i) the ancestor of *Y*. *pseudotuberculosis* harboring the ancestral *rpiA* gene acquired *hmsHFRS* and *rpiA2* by horizontal transfer and (ii) a clone of *Y*. *pseudotuberculosis* harboring *rpiA*, *rpiA2 and hmsHFRS* acquired *ymt* by horizontal transfer. Hence, our data emphasize that the accretion of genetic material by vertical and horizontal transfer in an ancestral strain produced a species with “pre-loaded” virulence factors, and thus had a key role in the emergence of a new species (Fig 13).

In addition to identifying the role of new genes in the infection of fleas, we reported that several genes needed for biofilm formation *in vitro* were not required *in vivo*. The fact that the DNA helicase Rep does not have a role in flea infection is surprising. This helicase helps to optimize chromosomal replication because it rescues transcription‐blocked replication forks by dislodging RNA polymerases from inverted *rrn* operons [34, 35]. Our unexpected finding suggests that either optimal chromosomal replication is not essential for flea blockage fleas or *Y*. *pestis* uses functionally redundant proteins (such as UvrD [36]) to rescue the absence of Rep. Functional redundancy would also explain the absence of any obvious role in flea infection for the other genes listed here. Regardless of the exact reason why a gene identified as being important in biofilm formation *in vitro* is not needed *in vivo*, our data show that there is little correlation between biofilms *in vitro* and in fleas, and thus highlight the specificity of the flea gut microenvironment. Indeed, our model suggests that the first step leading to the formation of a biofilm in flea is the induction of a mass trapping bacteria, rather than a simple attachment to a surface as is usually described for other biofilm-producing bacteria. In contrast to *rep*, the absence of a role for *ail* and *glpD* was expected. Indeed, our data on *ail* confirmed previous results [37]. The *glpD* gene is disrupted in all the most recent *Y*. *pestis* strains (Orientalis biovars) [38]. We cannot rule out a possible need for GlpD under specific conditions and in the older *Y*. *pestis* strains (the Antiqua and Medievalis biovars). In view of our present data and the knowledge that GlpD is not required for virulence in the rodent [39], it is nonetheless reasonable to presume that *glpD* is more likely in the process to be lost by the bacterium because unnecessary anymore. If this is true, it follows that the loss of a functional *glpD* does not confer a selective advantage—as it has been reported for several gene losses [40] but not for others [10, 41]. In other words, our data further confirm that the genome of *Y*. *pestis* is decaying.

Although our study focused on the mechanisms leading to flea blockage, it may provide clues to the mechanisms of early-phase transmission, defined as a biofilm (*hmsHFRS*-) -independent transmission of *Y*. *pestis* by unblocked fleas following a short extrinsic incubation period (≤4 days) [8, 42, 43]. Indeed, one can reasonably hypothesize that the mass molded into the shape of the proventriculus early after infection leads to early regurgitation of bacteria—notably because this mass is independent of *hmsHFRS*. In line with this idea and in agreement with our finding that *ymt* is essential for the production of the proventricular cast in *X*. *cheopis* infected with *Y*. *pestis*, the heterologous expression of *ymt* in *Y*. *pseudotuberculosis* is associated with the production of proventricular casts and early-phase transmission of *Y*. *pseudotuberculosis* by *X*. *cheopis* (the present study and [10]). However, *ymt* was reported to be dispensable for early-phase transmission in *X*. *cheopis* [44]. This apparent contradiction might be explained by the nature of the infected blood used in the different experiments. In our study and in the study of flea-borne transmission of *ymt*-expressing *Y*. *pseudotuberculosis*, the fleas were fed on mouse blood. The fleas used to investigate the role of *ymt* in early-phase transmission of *Y*. *pestis* were fed on rat blood. In contrast to mouse blood, rat blood promotes the regurgitation of bacteria located in the proventriculus into the esophagus after the infected blood meal (the so-called post-infection esophageal reflux (PIER)) because it contains oxyhemoglobin crystals that accumulate in the proventriculus [21]. Hence, if PIER is dependent on *ymt*, one can hypothesize that early-phase transmission after a rat blood meal results necessarily from the regurgitation of bacteria entrapped in the esophagus after the meal. If PIER is *ymt*-independent, however, this would indicate that early-phase transmission is linked to proventricular obstruction–as seems to occur when fleas feed on blood that does not promote PIER (i.e. mouse blood). In other words, the role of *ymt* might depend on the host blood source. If this model is accurate, it would mean that the acquisition of *ymt* extended *Y*. *pestis*’ ability to produce a transmissible infection after the flea feeds on a different rodent host. This idea will have to be investigated further.

Lastly, the production of a transmissible infection in fleas depends on the flea species, the blood source, and the environmental conditions [8, 21, 22, 43, 45]. In other words, the physical and genetic aspects of our model will need to be tested with regard to these potential sources of variation.

## Methods

### Ethics statement

The animal experiments (number CEEA 222012 and 2015102609372221) were approved by the local animal care and use committee (Committee 75) and were notified to the French authorities, in accordance with the current national legislation (government decree 2017–1411; Articles R, 214–87 to R, 214–126).

### Strains, plasmids, mutant production and complementation

*Y*. *pestis* KIM6+ was used, and *E. coli* DH5α was used to clone sequences of interest. The plasmids used in the present study are given in the S2 Table. *Y*. *pestis* mutants were generated using the lambda Red recombinase system, with the primer sets and plasmids described in S2 and S3 Tables [46, 47]. For single mutants, the coding sequence of interest (apart from the first and last 50 base pairs) was replaced by a kanamycin, trimethoprim, erythromycin, streptomycin or zeocin resistance cassette flanked by flippase recognition target sites that had been amplified from the vectors listed in S2 Table. To generate mutants with more than one deletion/insertion, resistance cassettes lacking flippase recognition target sites were used (S3 Table). Each mutation was checked in a PCR assay, using the primer sets shown in S3 Table.

For complementation, the gene of interest was amplified by PCR using the primer sets given in S3 Table, and then cloned into *E*. *coli* DH5α using the TA cloning kit with pCRII or pCR2.1 (ThermoFisher Scientific), according to the manufacturer’s instructions. The cloned sequences were checked by sequencing. *Y*. *pestis* mutant strains were transformed with the recombinant plasmid containing a WT copy of the gene of interest under the control of its own promoter or the P*lac* promoter.

To replace *rpiA* by *rpiA2* (or *vice versa*) on the chromosome, the lambda Red recombinase system was combined with the I-*Sce*I selection method [48]. To this end, we used the pEP1436 and pEP1446 plasmids (S2 Table) and the primer sets 5’-TGTTCTATAATAAGCTTTTGTTTTCATATCATAGGCAGTGAACTTACCGTGTAGGC TGGAGCTGCTTC-3’ / 5’-GCAAAAAATTATCAGGGTGAAGCCAAATAAACGACACCCT GAAAAATTCATATGAATATCCTCCTTAG-3’ to delete *rpiA*, and 5’-CAGAACTTGGCGA CCCCACCTTTATGTAGTCATGATCAGGAGTCACGAGTGTAGGCTGGAGCTGCTTC-3’ and 5’-TTGGTGCCAAGGTGACGCGACTCGCGACAGCGTCGAAAAAG GCCGTGAC ATATGAATATCCTCCTTAG-3’ to delete *rpiA2*. We then used the primer sets 5’-CAGAACTTGGCGACCCCACCTTTATGTAGTCATGATCAGGAGTCACGAATGACTCAGGATGAACTTAA-3’ / 5’-TTGGTGCCAAGGTGACGCGACTCGCGACAGCGTCGAAA AAGGCCGTGATTAGCCAATCACTTTAACGC-3’ to produce the *rpiA*-encompassing DNA regions used for the exchange, and 5’-TGTTCTATAATAAGCTTTTG TTTTCATATCATAGGCAGTGAACTTACCATGAGCAATCAACAAAATGA-3’ / 5’-GCAAA AAATTATCAGGGTGAAGCCAAATAAACGACACCCTGAAAAATTTTATCGCAGTTGAACGTAGG-3’ to produce the *rpiA2*-encompassing DNA regions. The sequence replacements were checked by sequencing and PCR assays.

### The biofilm assay

Biofilms were assayed as previously described [30]. Bacteria grown in lysogeny broth (LB) at 21°C overnight were washed and resuspended in LB supplemented with 4 mM MgCl_2_ and 4 mM CaCl_2_, brain heart infusion (BHI) or TMH medium, and then incubated at 21°C with shaking in a 24-well plate (2x10^6^ bacteria in 1 mL). Twenty-four hours later, the medium was removed, and a crystal violet dye solution (0.01%) was added to stain the attached bacterial biofilm. After a 15 min incubation at room temperature, the wells were washed three times with 2 mL of water. Next, 1.5 mL of an ethanol-acetone (80:20) solution was added to release the dye attached to the biofilm. Lastly, the solution was transferred to a 96-well plate, and the absorbance of each well was measured at 540 nm. The absorbance ratio for each strain of interest (relative to the parental strain) was calculated. Bacteria were also patched on Congo red agar media; after a 48-hour incubation at 21°C, the red pigmentation of the colonies was compared by eye with that of the WT control strain.

### Flea infection

Flea infection was assayed as previously described [23, 30]. *Xenopsylla cheopis* rat fleas were allowed to feed on heparinized mouse blood containing 5.10^8^ bacteria/mL (grown at 37°C overnight in BHI), using an artificial apparatus. One hour later, cohorts of fleas were collected for different purposes. Regardless of the purpose and whenever necessary, fleas were allowed to fed on neonate mice at 2, 6, 9, 13, 16, 20, 23 and 27-days post-infection. To monitor the gut blockage rate (i.e. the presence of fresh red blood in the flea’s foregut) twice a week for a 4-week period, cohorts composed of equal numbers of female and male fleas were collected immediately after feeding on neonate mouse. To monitor the time course of gut colonization, groups of females were collected at different times post-infection to individually plate them (after trituration) on BHI agar plates containing 1 μg/ml Irgasan and 10 μg/ml hemin. Forty-eight hours later, the CFUs were counted.

### Bright-field and fluorescence microscopy, and image processing

Cohorts of female fleas were infected with *Y*. *pestis* strains expressing the green fluorescent protein (GFP) on a pAcGFP plasmid (Addgene). When required, the flea’s gut was dissected under dissecting binoculars, immediately mounted in distilled water between a glass slide and a glass cover slip, and then observed under an Eclipse CiS microscope (Nikon). Bright-field and fluorescence images of the gut were acquired with a Sight DS-F1c camera (Nikon). Fluorescence images were processed using the curve adjustment tool in Adobe Photoshop CS4. This method highlights the GFP-expressing bacteria in blue and facilitates their identification within the proventriculus, which autofluoresces in green. The raw data can be provided on reasonable request. We also used Adobe Photoshop CS4 to generate merged images. After a portion of an image of interest had been selected with the cropping or lasso tool, it was cut and pasted to generate a new layer. The layer was colored (if required) using the hue and saturation tool, flipped with an angle of interest, and then merged with another image of interest by using the blending mode (“difference”, “lighten”, or “overlay”) than gave the best rendering.

### Lipo-oligosaccharide analysis

This analysis has been described previously [49]. Briefly, bacteria grown in LB at 21°C were centrifuged, resuspended in deoxycholate lysis buffer (2% sodium deoxycholate, 4% 2-mercaptoethanol, 10% glycerol, and 0.002% bromophenol blue in 1 M Tris-HCl buffer, pH 6.8), and then boiled at 100°C. After a 10 min incubation, the tubes were cooled and the suspension was treated with proteinase K (final concentration: 80 μg/mL) at 55°C overnight. Samples were analyzed using deoxycholate-PAGE, as described previously [50].

### Antimicrobial peptide resistance assay

One hundred microliters of LB containing 5x10^4^ bacteria grown at 21°C or 37°C in LB were added to 96-well plates containing 20 μL per well of LB supplemented with different concentrations of polymyxin B (range: 90 to 2.3 μg/mL). Plates were incubated for 48-hours at 21°C. The MIC was defined as the lowest concentration of drug that prevented visible growth after incubation.

### Ribose phosphate isomerase activity

PCR fragments encompassing the open reading frame of *rpiA* and *rpiA2* flanked with *NdeI* / *XbaI* restriction sites were amplified (using the primer sets 5’-CGCCATATGACTCAGGATGAACTT-3’ / 5’-GGCTCTAGATTAGCCAATCACTTTAACG C-3’ and 5’-CGCCATATGAGCAATCAACAAAATGACGC-3’ / 5‘-CGCTCTAGATTATCGC AGTTGAACGTAGG-3’) and then cloned into the pCRII plasmid using a TA cloning kit (Thermofisher Scientific). After the sequence had been checked, the *Nde*I / *Xba*I fragments containing the gene of interest were subcloned into the pETMCN-EATNH expression vector [51]. The recombinant plasmids were used to produce RpiA and RpiA2 *in vitro* using the PURExpress In Vitro Protein Synthesis Kit (New England Biolabs). A sample of each preparation was collected and tested for RpiA activity, as described previously [52].

### Transmission electron microscopy

Samples were fixed at 4°C overnight with 4% paraformaldehyde and 1% glutaraldehyde in 0.1 M sodium cacodylate pH 6.8 buffer, and post-fixed with 1% osmium tetroxide and 1.5% potassium ferricyanide and then with 1% uranyl acetate (all in distilled water at room temperature in the dark, for 1 hour). After washing, the samples were dehydrated using increasingly concentrated ethanol solutions. Lastly, samples were infiltrated with epoxy resin and cured at 60°C for 48 hours. Sections (thickness: 70–80 nm) on formvar-coated grids were observed at 80 kV with a Hitachi H7500 transmission electron microscope (AMT, France), and images were acquired with a 1 Mpixel digital camera (AMT).

### Statistical analysis

All statistical analyses were performed using the GraphPad Prism software (GraphPad Software Inc., La Jolla, CA). The tests and *p* value thresholds used are indicated in the figure legends. All tests were two-sided.

## Supporting information

S1 Fig The flea gut. The bright-field microscopy image shows part of the insect's digestive tract. The esophagus (E), the proventriculus (PV; brown) and the midgut (MG) are clearly visible. The proventriculus is a valve covered with inward-facing spines, and autofluoresces in green.
(PDF)

S2 Fig Practical examples highlighting the presence of a free-floating proventricular cast in the midgut. (A) shows a simplified representation of the anatomy of the proventriculus and of the different types of mass (cast) attached to it. The cast has a “head” and a long tail (i.e. a comet-/mushroom-like shape), which can easily be recognized in some of the images in panel (B). The red arrowheads indicate the boundaries between the three regions of the proventriculus. (B) and (C) describe the steps leading to the identification of a relatively intact (B) or partly dislocated (C) free-floating proventricular cast in the midgut. (B) The first and second columns respectively show bright-field and fluorescent microscopy images of guts infected with a fluorescent *Y*. *pestis*. The proventriculus autofluoresces in green, and fluorescent *Y*. *pestis* appears in blue. The third column shows the proventriculus and the free-floating mass(es) located in the midgut, after extraction from the photo in the second column and rotation when needed. The red lines represent the boundaries between the anterior (Ant.) and spined (Sp.) regions, the spined and stomodaeum (St.) regions, or the stomodaeum region and the midgut. The pink arrowheads highlight the presence of bacteria (when present) used to show the complementary colonization profiles for the proventriculus and the mass located in the midgut (depicted in the fourth column). The latter column shows merged images of the proventriculus and the mass(es) shown in third column. (C) The same situation as in (B), except that the circles show an intact or dislocated proventricular cast. In the third column, the dislocated cast’s various parts were colored, rotated and matched (using Photoshop CS4) to show their complementarity. In C, the extension of the mass in the esophagus (E) is presumably due to an artifact associated with the methodology used to collect and observe the gut under the microscope. All the merged images shown in the far right-hand column were generated as described in the Method section, using the “overlay” blending mode in Adobe Phothoshop CS4.
(PDF)

S3 Fig A putative proventricular cast in the midgut of a flea collected 6 hours post-infection. The image in the bottom inset is a merged image generated using the insets showing the proventriculus and the free-floating mass located into the midgut.
(PDF)

S4 Fig The Δ*rpiA* mutant blocks fleas stochastically. The number of fleas (out of a total of 300) displaying blockage at different time intervals after feeding on blood contaminated by the WT (full bars) or the *ΔrpiA* mutant (hatched bars) is shown. Data from three independent experiments are stacked. Each color corresponds to a different experiment.
(PDF)

S5 Fig The Δ*rep*, Δ*hdfR*, Δ*rsx*, Δ*glpD* and Δ*ail Y*. *pestis* mutants effectively block fleas, even though their ability to produce biofilm *in vitro* is impaired. The mean ± SEM values of two experiments are shown. However, data from only one experiment using the Δ*ail* mutant are shown because *ail* is known not to be required for flea blockage [37]. The blockage rates in the mutants did not differ significantly from those observed for the WT strain (p>0.1 in a one-way analysis of variance for all comparisons).
(PDF)

S6 Fig The core oligosaccharide of *Y*. *pestis* lacking *rpiA*, *rpe* or *talB* is not truncated. A silver-stained deoxycholate-PAGE gel is shown.
(PDF)

S7 Fig *rpiA* and *rpiA*2 are needed to maintain *Y*. *pestis* in the proventriculus after feeding. Fluorescence images of the proventriculus (in green) infected with the WT, Δ*rpiA* or Δ*rpiA* Δ*rpiA*2 strains (in blue) before and immediately after feeding, acquired 2, 6 and 13 days post-infection.
(PDF)

S8 Fig RpiA activity is needed for the production of a thick mass in the flea gut. A scanning electron micrograph of the biofilms produced by the WT and the Δ*rpiA* mutant in the flea at days 6 and 13 post-infection. Each photo was taken using the mass collected from a different flea (i.e. two individuals per day/strain).
(PDF)

S9 Fig Role of pentose related metabolic pathways in flea blockage. The figure has been constructed from 8 independent experiments in which the blocking rate was measured for a strain lacking a gene of interest and for the parental strain. The bars represent the mean ± SEM from 8 independent experiments (wild-type control strain), two independent experiments (Δ*rpe* and Δ*rpe* Δ*xylAB1* Δ*xylB2* Δ*araD1* Δ*araD2*) and one experiment for all the other mutants. The symbols represents the value obtained in the different experiments. The blockage rate given for the Δ*rpe* Δ*xylAB1* Δ*xylB2* Δ*araD1* Δ*araD2* mutant was obtained using two independent mutants; one was generated by deleting successively *rpe*, *araD1*, *araD2*, *xylAB1* then *xylB2* whereas the other was generated by deleting successively *araD1*, *araD2*, *xylAB1*, *xylB2* then *rpe*.
(PDF)

S1 Table Percentage of infected fleas. (PDF)

S2 Table Plasmids used in the study. (PDF)

S3 Table Primer sets used in the study. (PDF)

S1 Text (PDF)
