# Supplementary material for: A refined model of how Yersinia pestis produces a transmissible infection in its flea vector
Source: PLoS Pathog. 2020 Apr 15;16(4):e1008440. doi: 10.1371/journal.ppat.1008440 (PMC7185726; doi:10.1371/journal.ppat.1008440)
Supplement: S1 Fig — The bright-field microscopy image shows part of the insect's digestive tract. The esophagus (E), the proventriculus (PV; brown) and the midgut (MG) are clearly visible. The proventriculus is a valve covered with inward-facing spines, and autofluoresces in green. (PDF) [file ppat.1008440.s001.pdf]

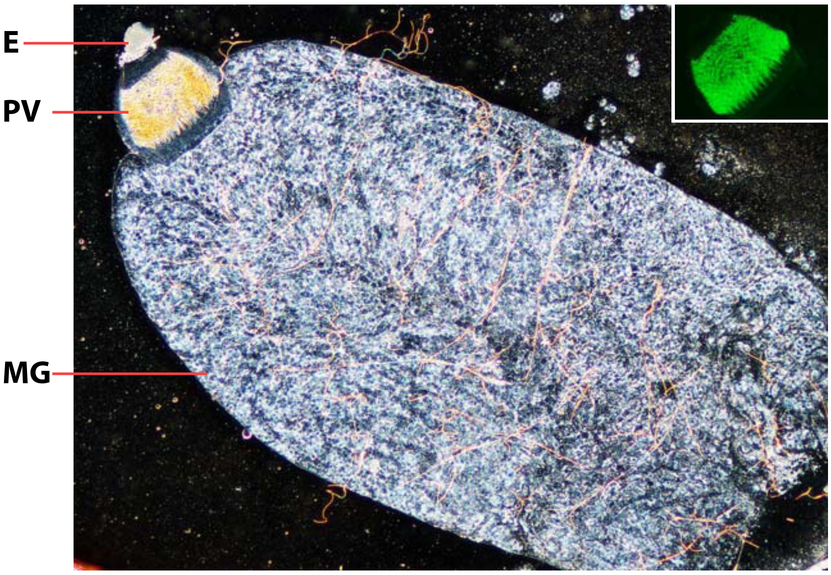

Figure S1. The flea gut. The bright-field microscopy image shows part of the insect's digestive tract. The esophagus (E), the proventriculus (PV; brown) and the midgut (MG) are clearly visible. The proventriculus is a valve covered with inward-facing spines, and autofluoresces in green.
