# Supplementary material for: A refined model of how Yersinia pestis produces a transmissible infection in its flea vector
Source: PLoS Pathog. 2020 Apr 15;16(4):e1008440. doi: 10.1371/journal.ppat.1008440 (PMC7185726; doi:10.1371/journal.ppat.1008440)
Supplement: S2 Fig — (A) shows a simplified representation of the anatomy of the proventriculus and of the different types of mass (cast) attached to it. The cast has a “head” and a long tail (i.e. a comet-/mushroom-like shape), which can easily be recognized in some of the images in panel (B). The red arrowheads indicate the boundaries between the three regions of the proventriculus. (B) and (C) describe the steps leading to the identification of a relatively intact (B) or partly dislocated (C) free-floating proventricular cast in the midgut. (B) The first and second columns respectively show bright-field and fluorescent microscopy images of guts infected with a fluorescent Y. pestis. The proventriculus autofluoresces in green, and fluorescent Y. pestis appears in blue. The third column shows the proventriculus and the free-floating mass(es) located in the midgut, after extraction from the photo in the second column and rotation when needed. The red lines represent the boundaries between the anterior (Ant.) and spined (Sp.) regions, the spined and stomodaeum (St.) regions, or the stomodaeum region and the midgut. The pink arrowheads highlight the presence of bacteria (when present) used to show the complementary colonization profiles for the proventriculus and the mass located in the midgut (depicted in the fourth column). The latter column shows merged images of the proventriculus and the mass(es) shown in third column. (C) The same situation as in (B), except that the circles show an intact or dislocated proventricular cast. In the third column, the dislocated cast’s various parts were colored, rotated and matched (using Photoshop CS4) to show their complementarity. In C, the extension of the mass in the esophagus (E) is presumably due to an artifact associated with the methodology used to collect and observe the gut under the microscope. All the merged images shown in the far right-hand column were generated as described in the Method section, using the “overlay” blending mode in Ad [file ppat.1008440.s002.pdf]

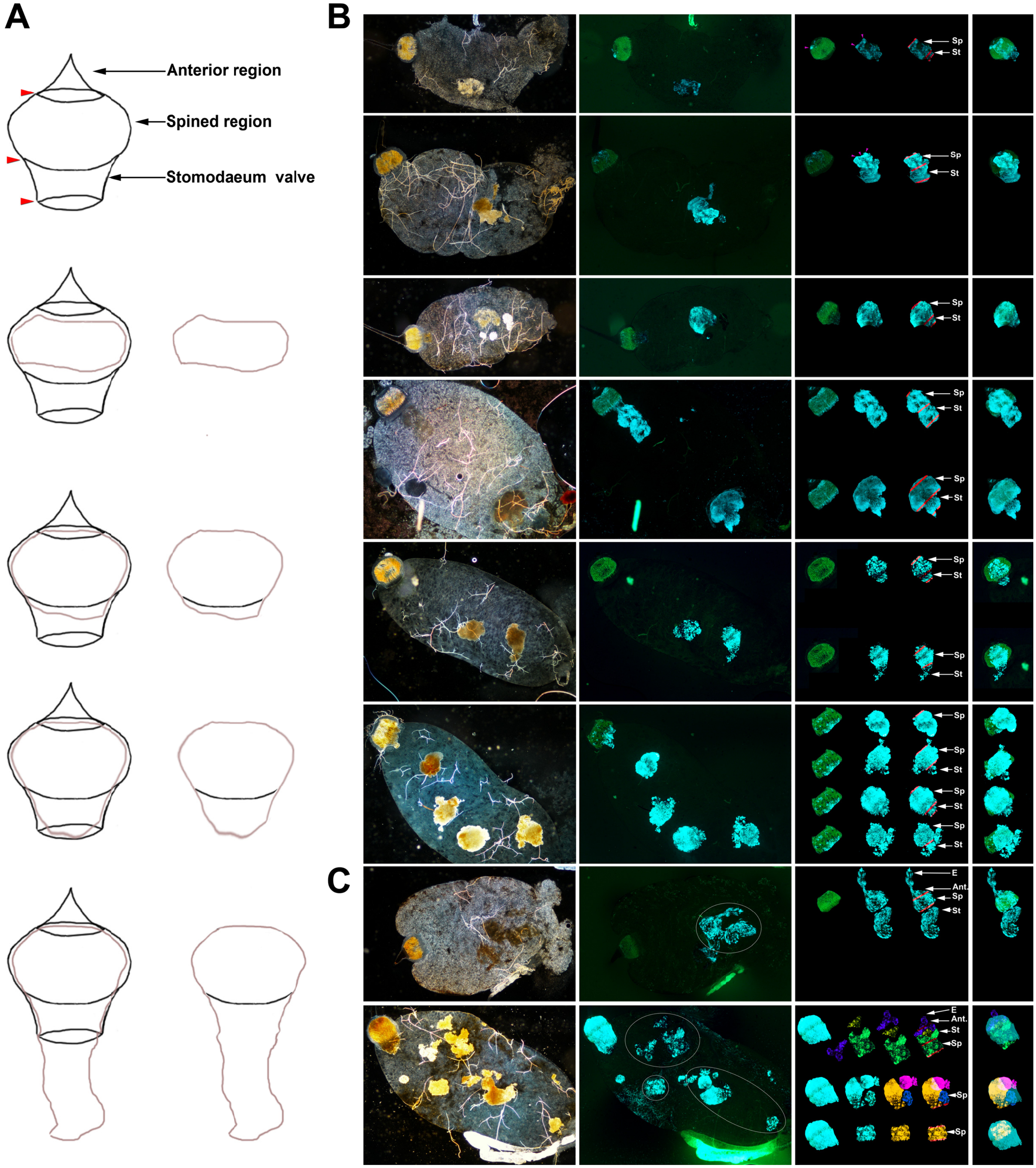

Figure S2. Practical examples highlighting the presence of a free-floating proventricular cast in the midgut. (A) shows a simplified representation of the anatomy of the proventriculus and of the different types of mass (cast) attached to it. The cast has a "head" and a long tail (i.e. a comet-/mushroom-like shape), which can easily be recognized in some of the images in panel (B). The red arrowheads indicate the boundaries between the three regions of the proventriculus. (B) and (C) describe the steps leading to the identification of a relatively intact (B) or partly dislocated (C) free-floating proventricular cast in the midgut. (B) The first and second columns respectively show bright-field and fluorescent microscopy images of guts infected with a fluorescent Y. pestis. The proventriculus autofluoresces in green, and fluorescent Y. pestis appears in blue. The third column shows the proventriculus and the free-floating mass(es) located in the midgut, after extraction from the photo in the second column and rotation when needed. The red lines represent the boundaries between the anterior (Ant.) and spined (Sp.) regions, the spined and stomodaeum (St.) regions, or the stomodaeum region and the midgut. The pink arrowheads highlight the presence of bacteria (when present) used to show the complementary colonization profiles for the proventriculus and the mass located in the midgut (depicted in the fourth column). The latter column shows merged images of the proventriculus and the mass(es) shown in third column. (C) The same situation as in (B), except that the circles show an intact or dislocated proventricular cast. In the third column, the dislocated cast's various parts were colored, rotated and matched (using Photoshop CS4) to show their complementarity. In C, the extension of the mass in the esophagus (E) is presumably due an artifact associated with the methodology used to collect and observe the gut under the microscope. All the merged images shown in the far right-hand column were generated as described in the Method section, using the "overlay" blending mode in Adobe Photoshop CS4.
