# Supplementary material for: A refined model of how Yersinia pestis produces a transmissible infection in its flea vector
Source: PLoS Pathog. 2020 Apr 15;16(4):e1008440. doi: 10.1371/journal.ppat.1008440 (PMC7185726; doi:10.1371/journal.ppat.1008440)
Supplement: S3 Fig — The image in the bottom inset is a merged image generated using the insets showing the proventriculus and the free-floating mass located into the midgut. (PDF) [file ppat.1008440.s003.pdf]

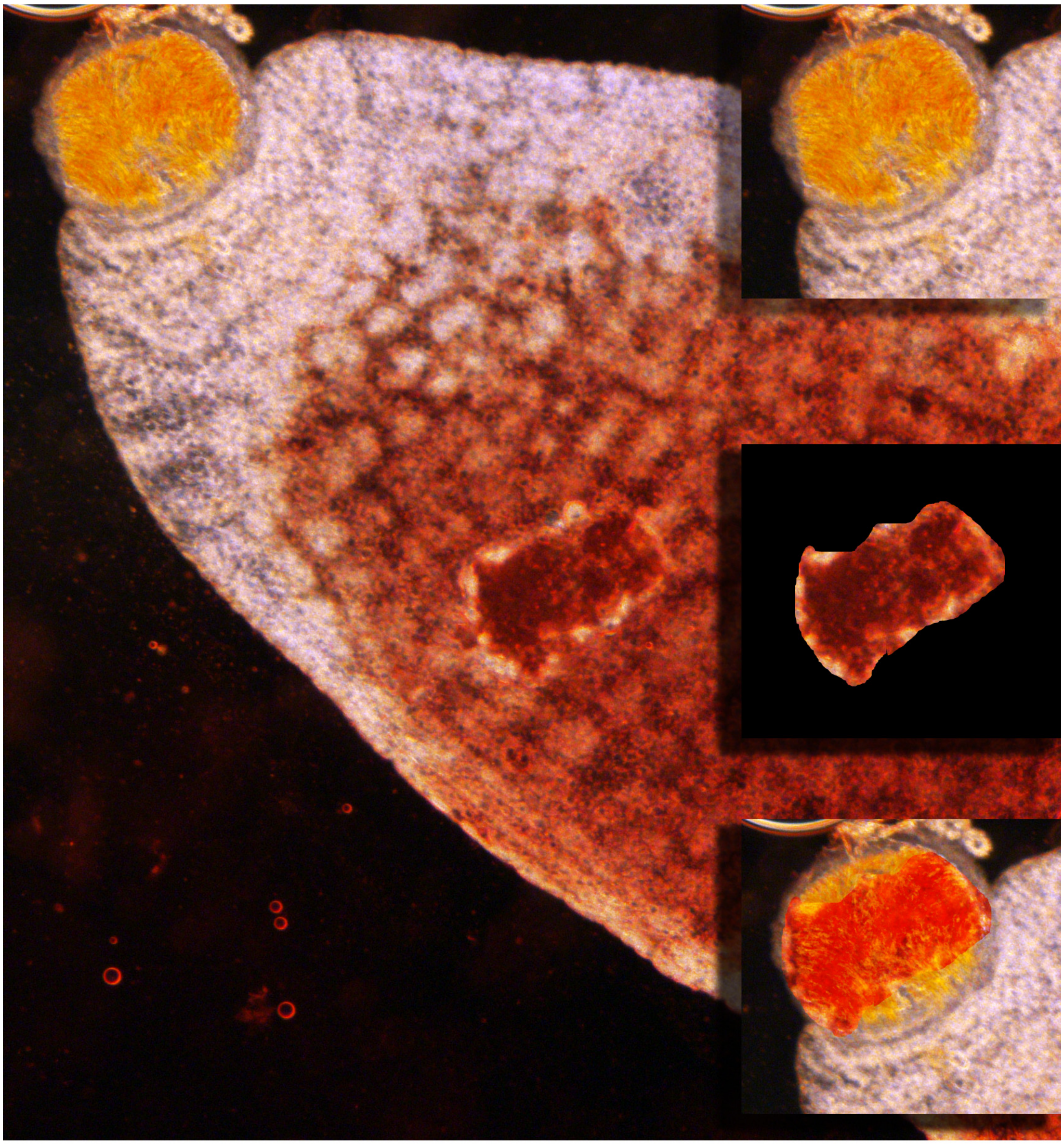

Figure S3. Presence of a putative proventricular cast in the midgut of flea collected 6-h post-infection. The image in the bottom inset is a merged image generated using the insets showing the proventriculus and the free-floating mass located int the midgut.
