# Supplementary material for: A refined model of how Yersinia pestis produces a transmissible infection in its flea vector
Source: PLoS Pathog. 2020 Apr 15;16(4):e1008440. doi: 10.1371/journal.ppat.1008440 (PMC7185726; doi:10.1371/journal.ppat.1008440)
Supplement: S4 Fig — The number of fleas (out of a total of 300) displaying blockage at different time intervals after feeding on blood contaminated by the WT (full bars) or the ΔrpiA mutant (hatched bars) is shown. Data from three independent experiments are stacked. Each color corresponds to a different experiment. (PDF) [file ppat.1008440.s004.pdf]

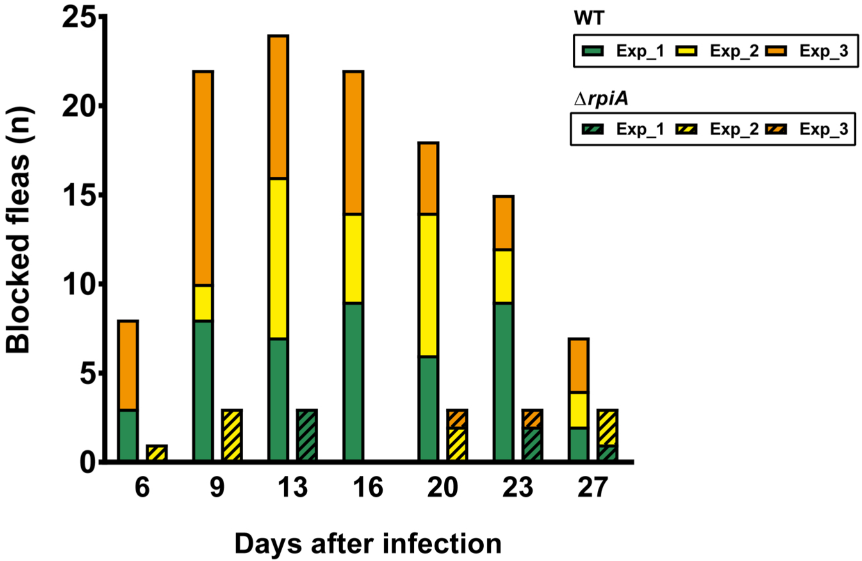

Figure S4. The Δ*rpiA* mutant blocks fleas stochastically. The number of fleas (out of a total of 300) displaying blockage at different time intervals after feeding on blood contaminated by the WT (full bars) or the Δ*rpiA* mutant (hatched bars) is shown. Data from three independent experiments are stacked. Each color represents a different experiment.
