# Supplementary material for: A refined model of how Yersinia pestis produces a transmissible infection in its flea vector
Source: PLoS Pathog. 2020 Apr 15;16(4):e1008440. doi: 10.1371/journal.ppat.1008440 (PMC7185726; doi:10.1371/journal.ppat.1008440)
Supplement: S5 Fig — The mean ± SEM values of two experiments are shown. However, data from only one experiment using the Δail mutant are shown because ail is known not to be required for flea blockage [37]. The blockage rates in the mutants did not differ significantly from those observed for the WT strain (p>0.1 in a one-way analysis of variance for all comparisons). (PDF) [file ppat.1008440.s005.pdf]

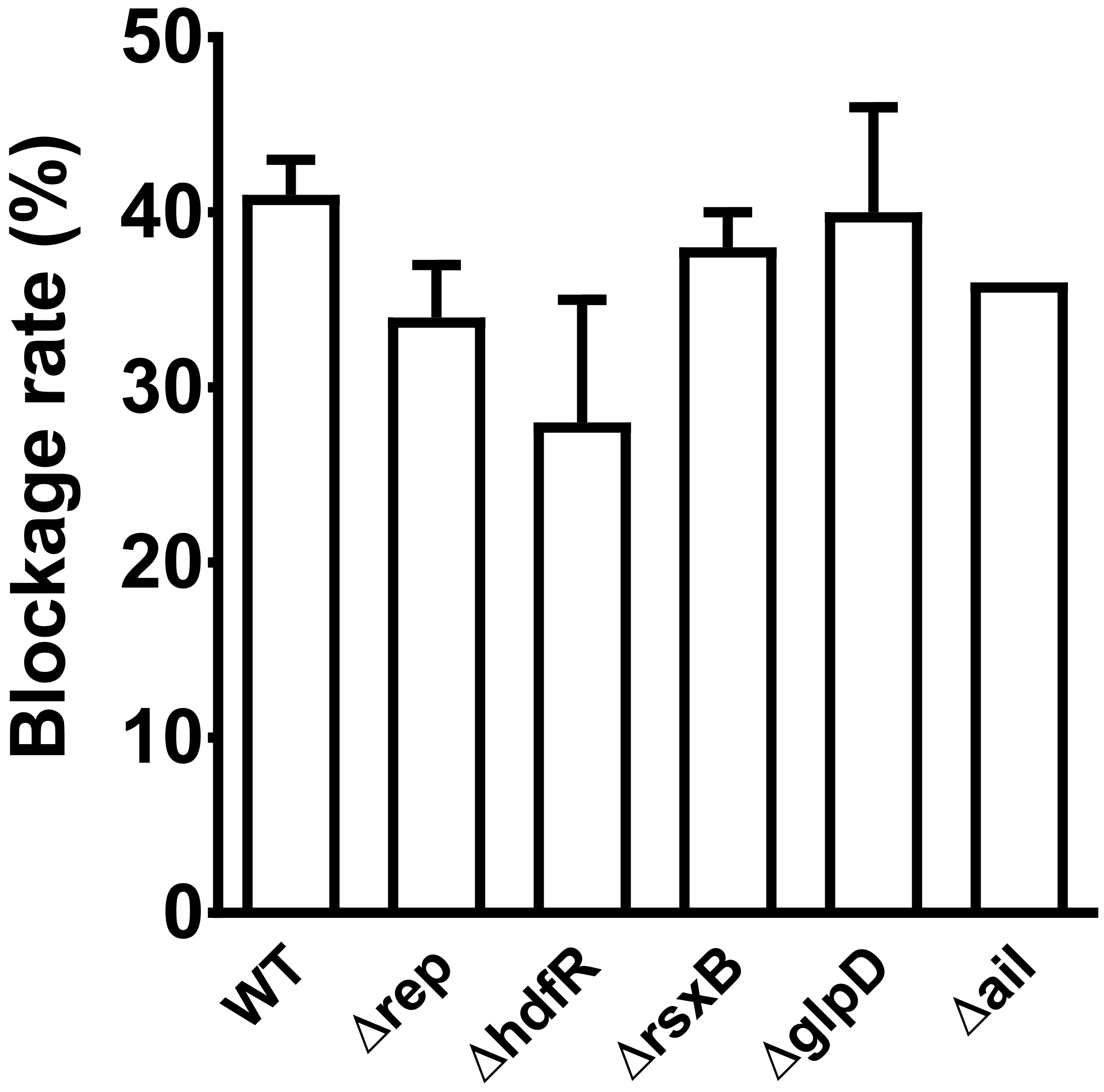

Figure S5. The Δ*rep*, Δ*hdfR*, Δ*rsx*, Δ*glpD* and Δ*ail Y.pestis* mutants effectively block fleas, even though their ability to produce biofilm in vitro is impaired. The mean ± SEM values of two experiments are shown. However, data from only one experiment using the Δ*ail* mutant are shown because *ail* is known not to be required for flea blockage. The blockage rates in the mutants did not differ significantly from those observed for the WT strain ($p>0.1$ in a one-way analysis of variance for all comparisons).
