# Supplementary material for: A refined model of how Yersinia pestis produces a transmissible infection in its flea vector
Source: PLoS Pathog. 2020 Apr 15;16(4):e1008440. doi: 10.1371/journal.ppat.1008440 (PMC7185726; doi:10.1371/journal.ppat.1008440)
Supplement: S6 Fig — A silver-stained deoxycholate-PAGE gel is shown. (PDF) [file ppat.1008440.s006.pdf]

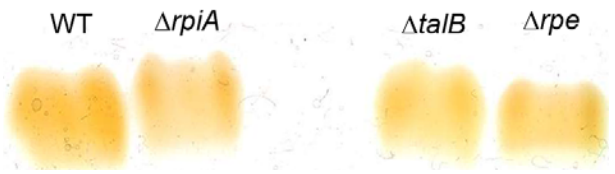

Figure S6. The core oligosaccharide of *Y. pestis* lacking *rpiA*, *rpe* or *talB* is not truncated. A silver-stained deoxycholate-PAGE gel is shown.
