# Supplementary material for: A refined model of how Yersinia pestis produces a transmissible infection in its flea vector
Source: PLoS Pathog. 2020 Apr 15;16(4):e1008440. doi: 10.1371/journal.ppat.1008440 (PMC7185726; doi:10.1371/journal.ppat.1008440)
Supplement: S7 Fig — Fluorescence images of the proventriculus (in green) infected with the WT, ΔrpiA or ΔrpiA ΔrpiA2 strains (in blue) before and immediately after feeding, acquired 2, 6 and 13 days post-infection. (PDF) [file ppat.1008440.s007.pdf]

Part 1/2

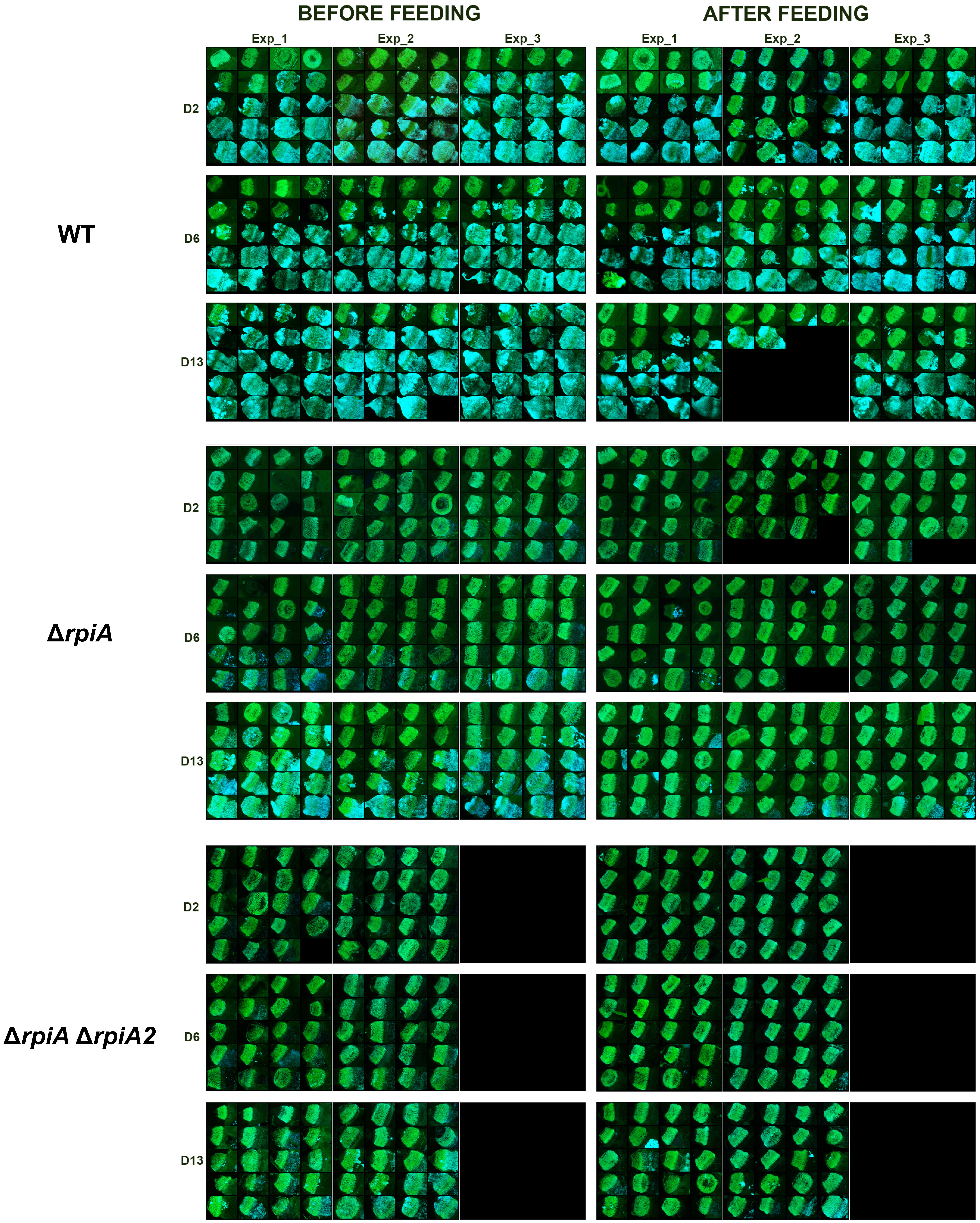

Figure S7. *rpiA* and *rpiA2* are needed to maintain *Y. pestis* in the proventriculus after feeding. Fluorescence images of the proventriculus (in green) infected with the WT, Δ*rpiA* or Δ*rpiA* Δ*rpiA2* strains (in blue) before and immediately after feeding, acquired 2, 6 and 13 days post-infection.

Part_2/2

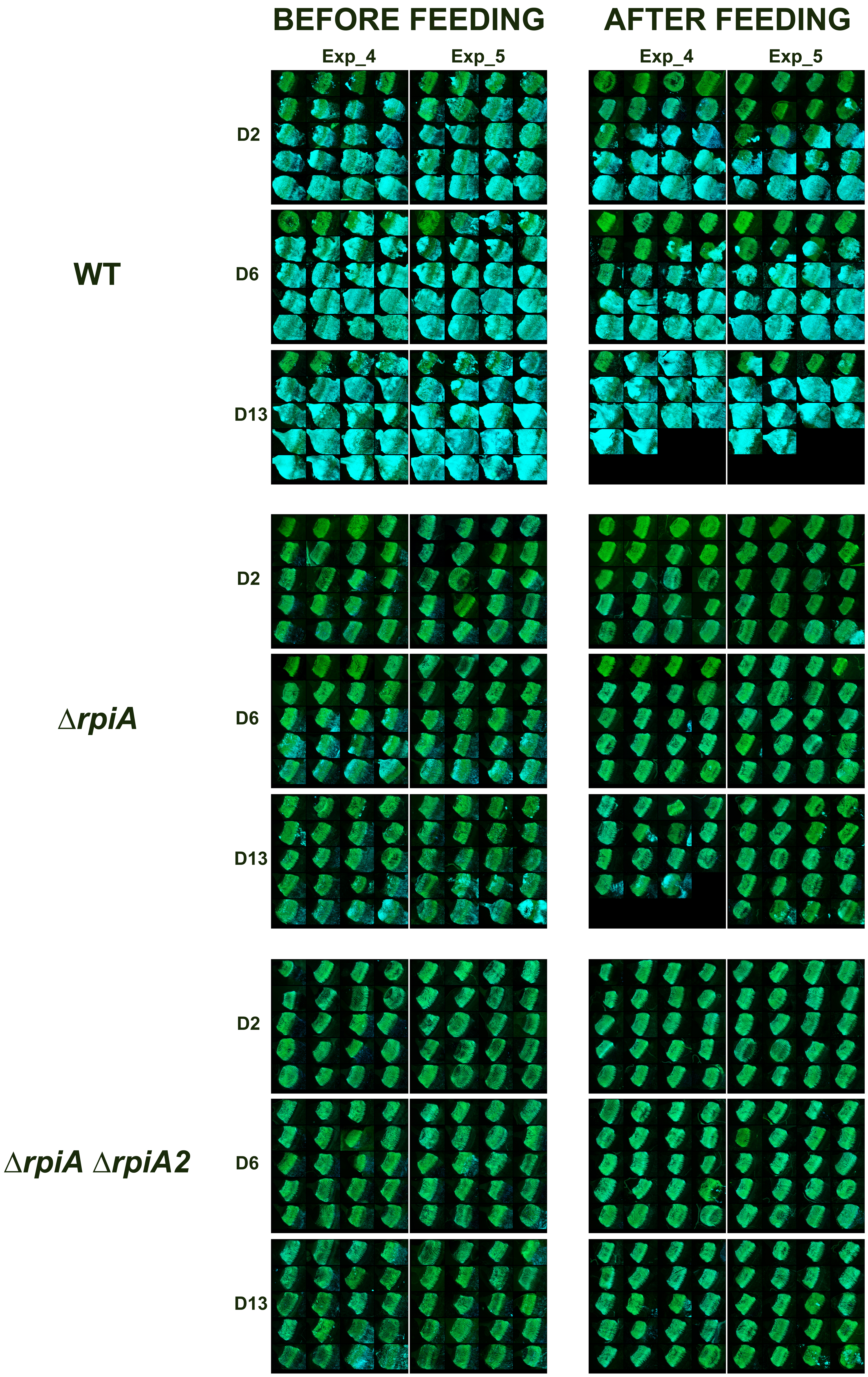

BEFORE FEEDING
AFTER FEEDING
Exp_4
Exp_5
Exp_4
Exp_5
WT
D2
D6
D13
ΔrpiA
D2
D6
D13
ΔrpiA ΔrpiA2
D2
D6
D13
