# Supplementary material for: A refined model of how Yersinia pestis produces a transmissible infection in its flea vector
Source: PLoS Pathog. 2020 Apr 15;16(4):e1008440. doi: 10.1371/journal.ppat.1008440 (PMC7185726; doi:10.1371/journal.ppat.1008440)
Supplement: S8 Fig — A scanning electron micrograph of the biofilms produced by the WT and the ΔrpiA mutant in the flea at days 6 and 13 post-infection. Each photo was taken using the mass collected from a different flea (i.e. two individuals per day/strain). (PDF) [file ppat.1008440.s008.pdf]

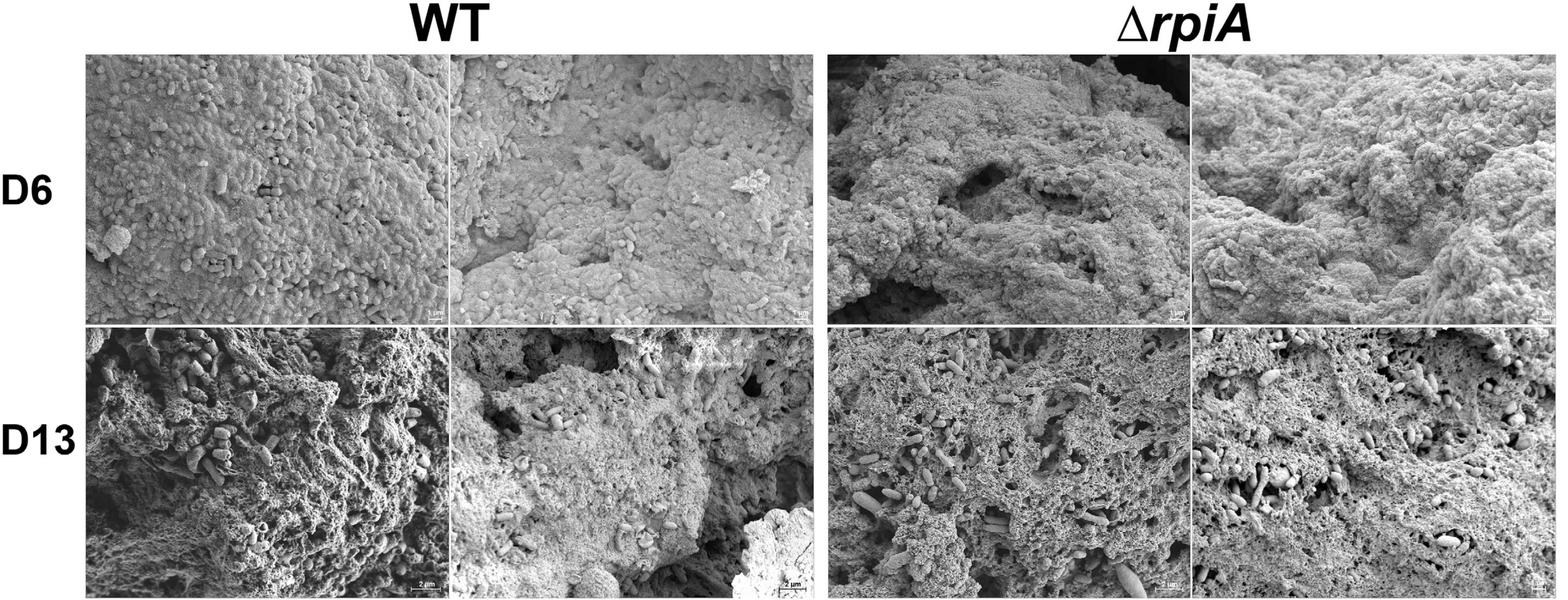

Figure S8. RpiA activity is needed for the production of a thick mass in the flea gut. A scanning electron micrograph of the biofilms produced by the WT and the Δ*rpiA* mutant in the flea at days 6 and 13 post-infection. Each photo was taken using the mass collected from a different flea (i.e. two individuals per day/strain).
