# Supplementary material for: A refined model of how Yersinia pestis produces a transmissible infection in its flea vector
Source: PLoS Pathog. 2020 Apr 15;16(4):e1008440. doi: 10.1371/journal.ppat.1008440 (PMC7185726; doi:10.1371/journal.ppat.1008440)
Supplement: S9 Fig — The figure has been constructed from 8 independent experiments in which the blocking rate was measured for a strain lacking a gene of interest and for the parental strain. The bars represent the mean ± SEM from 8 independent experiments (wild-type control strain), two independent experiments (Δrpe and Δrpe ΔxylAB1 ΔxylB2 ΔaraD1 ΔaraD2) and one experiment for all the other mutants. The symbols represents the value obtained in the different experiments. The blockage rate given for the Δrpe ΔxylAB1 ΔxylB2 ΔaraD1 ΔaraD2 mutant was obtained using two independent mutants; one was generated by deleting successively rpe, araD1, araD2, xylAB1 then xylB2 whereas the other was generated by deleting successively araD1, araD2, xylAB1, xylB2 then rpe. (PDF) [file ppat.1008440.s009.pdf]

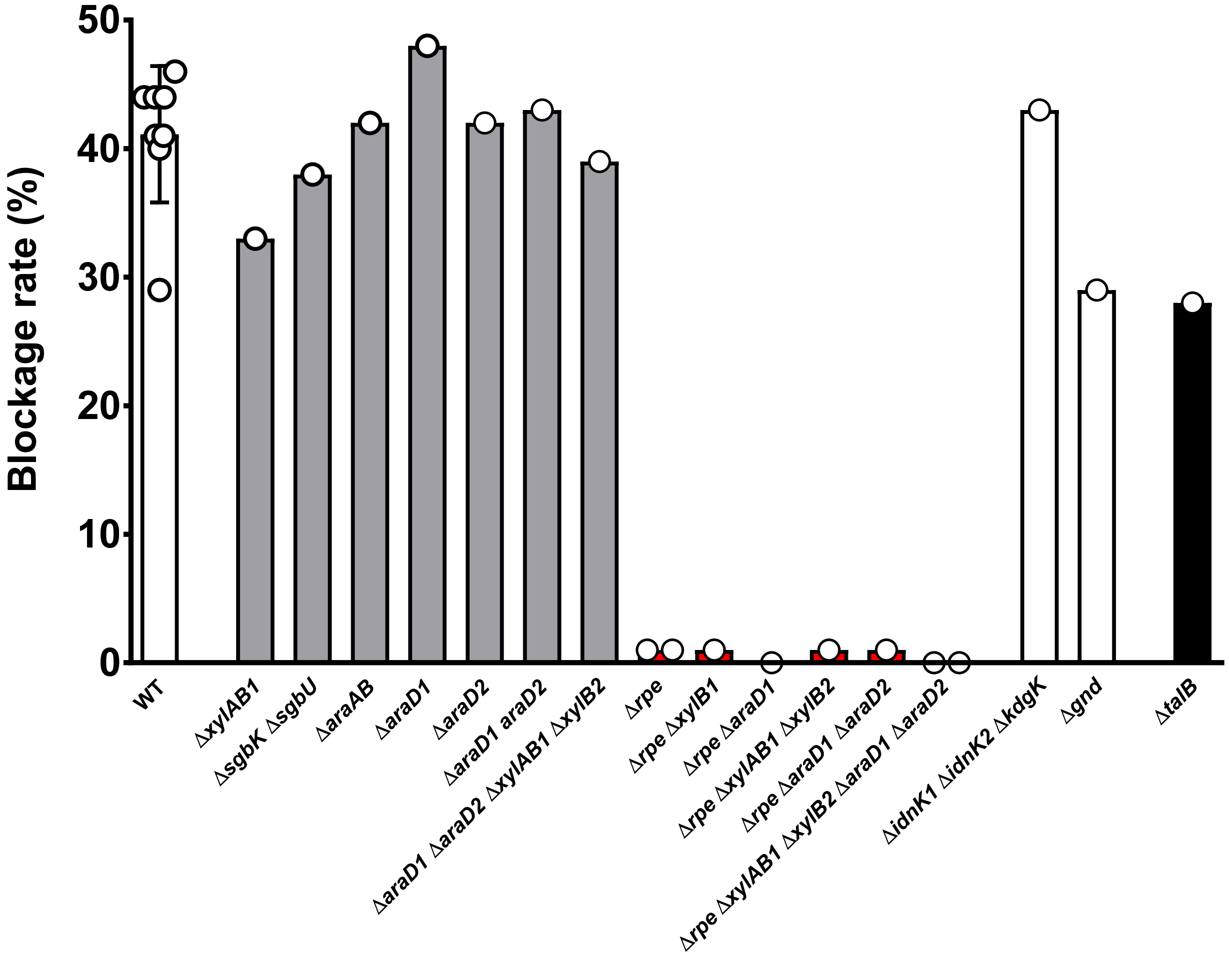

Figure S9. Role of pentose related metabolic pathways in flea blockage. The figure has been constructed from 8 independent experiments in which the blocking rate was measured for a strain lacking a gene of interest and for the parental strain. The bars represent the mean ± SEM from 8 independent experiments (wild-type control strain), two independent experiments (Δ*rpe* and Δ*rpe* Δ*xylAB1* Δ*xylB2* Δ*araD1* Δ*araD2*) and one experiment for all the other mutants. The symbol represent the value obtain in the different experiments. The blockage rate given for the Δ*rpe* Δ*xylAB1* Δ*xylB2* Δ*araD1* Δ*araD2* mutant was obtained using two independent mutants; one was generated by deleting successively *rpe*, *araD1,* araD2*,* xyl*AB1* then *xylB2* whereas the other was generated by deleting successively *araD1*, *araD2*, *xylAB1*, *xylB2, rpe*.
