# Supplementary material for: A refined model of how Yersinia pestis produces a transmissible infection in its flea vector
Source: PLoS Pathog. 2020 Apr 15;16(4):e1008440. doi: 10.1371/journal.ppat.1008440 (PMC7185726; doi:10.1371/journal.ppat.1008440)
Supplement: S1 Table — (PDF) [file ppat.1008440.s010.pdf]

Table S1. Pourcentage of infected fleas.

Week post-infection	Strain	Infected fleas (median [range])	positive fleas/fleas used in total
1	WT<br>Δ*rpiA*	95 .0% [85-100%]<br>94.7% [80-95%]	56/60<br>53/59
4	WT<br>Δ*rpiA*	87.2% [84.2-90%]<br>85.0% [80-90%]	34/39<br>34/40

a, cumulative results of 3 (1 week) and 2 (4 weeks) experiments in which 19 to 20 fleas were collected during each experiment.
