# Supplementary material for: A refined model of how Yersinia pestis produces a transmissible infection in its flea vector
Source: PLoS Pathog. 2020 Apr 15;16(4):e1008440. doi: 10.1371/journal.ppat.1008440 (PMC7185726; doi:10.1371/journal.ppat.1008440)
Supplement: S2 Table — (PDF) [file ppat.1008440.s011.pdf]

Supplementary Table 2. Plasmids used in the study.

Plasmid	Relevant characteristics[a]	Reference or origin[b]
pCRII	cloning vector; Ap and Km	Invitrogen
pCR2.1	cloning vector; Ap and Km	Invitrogen
pAcGFP	vector encoding the green fluorescent protein AcGFP1, Ap	Addgene
pETMCN-EATNH	expression vector, Km	1
pKD4	ori6K vector, source of Km^R cassette, Km flanked by FRT sites	2
pEP1013	vector encoding the Red recombinase, Ap	3
pEP1436	vector derived from pEP1013 and encoding the endonuclease I-*Sce* I, Ap	This work
pEP1042	derived from pKD4, source of Zeo^R cassette flanked by FRT sites	3
pEP1087	derived from pKD4, source of Tp^R cassette flanked by FRT sites	3
pEP1237	derived from pKD4, source of Er^R cassette flanked by FRT sites	This work
pEP1326	derived from pKD4, source of Sm^R cassette by FRT sites	This work
pEP864	derived from pKD4, source of Er^R cassette (no FRT)	This work
pEP865	derived from pKD4, source of Sm^R cassette (no FRT)	This work
pEP866	derived from pKD4, source of Zeo^R cassette (no FRT)	This work
pEP1446	derived from pKD4, source of Km^R cassette with I-*Sce* I site (no FRT)	This work
pEP1454	derived from pKD4, source of Tp^R cassette (no FRT)	This work
pAil	pBluescript KS(−) expressing *ail*	4
pCH16	pACYC177 constitutively expressing *ymt*	5
pAD1	pCRII topo containing *rpiA* (y3302) under the control of its putative promoter	This work
pAD2	pCR2.1 containing *rpiA2* (y2892) under plac promoter	This work
pAD3	pCRII topo containing *rpiA* (y3302) open reading frame	This work
pAD4	pCRII topo containing *rpiA2* (y2892) open reading frame	This work
pAD5	pETMCN-EATNHΩ, NdeI / XbaI insert from pAD3	This work
pAD6	pETMCN-EATNHΩ, NdeI / XbaI insert from pAD4	This work
pAD7	pCRII containing *rep* under the control of its putative promoter	This work
pAD8	pCRII containing *hdfR* under the control of its putative promoter	This work
pAD9	pCRII containing *glpD* under the control of its putative promoter	This work

a, Ap^R, Km^R, Tp^R, Zeo^R, Er^R, Sm^R resistance to ampicilin, kanamycin, trimethoprim, zeocin, erythromycin and streptomycin respectively

b, references are provided in supplementary text
