# Supplementary material for: A refined model of how Yersinia pestis produces a transmissible infection in its flea vector
Source: PLoS Pathog. 2020 Apr 15;16(4):e1008440. doi: 10.1371/journal.ppat.1008440 (PMC7185726; doi:10.1371/journal.ppat.1008440)
Supplement: S3 Table — (PDF) [file ppat.1008440.s012.pdf]

Supplementary Table 3. Primer sets used in the study

ORF(s)	PRIMER SET (5' -> 3') USED TO		
	GENERATE THE MUTATION[a]	VERIFY THE MUTATION	COMPLEMENTATION
y0046	ATGAGCCAAACCGCTAGTTCTACCTTAAAAGGCCAATGTATTGCGGAGTTGTGTAGGCTGGAGCTGCTTC	ATGTAGCCAATCCCGCCCTG	-
	TTATGCTTTACGTTCTGTGGTTATCGTTGTGGATTCTTCATCCTCAAGGGCATATGAATATCCTCCTTAG	GCTGCCTACATGCGACTCCA	-
y0063-y0065	TTAAGTGTGGACACTTTGTGGACGCGTTTCGGCAGCTAATCCACCCTTTAGTGTAGGCTGGAGCTGCTTC	GCCATGTCGAAATGGCCAAA	-
	TTATTGAGGCGACTTAGGGGCAGCATGGGTACGCATTAACGCCACACGGTCATATGAATATCCTCCTTAG	TAGAGCACGTCCGAGTAACA	-
y0077-78	ATGTCGAGCACGAAAAAACCACTGGTCCTGACGATTCTTGATGGTTACGGGTGTAGGCTGGAGCTGCTTC	AAACTGCGAACAATCTCGGG	-
	CTATCTTCCTAACCAGGGCTGTGGGTTAACGGCTTGTCCCTGACGGCGGACATATGAATATCCTCCTTAG	CATCGGCAAGTGAATCAGGA	-
y0090	GTGCCTGAATTACCAGAAGTTGAAACCAGCCGACGCGGGATCGAACCTTAGTGTAGGCTGGAGCTGCTTC	TTAATCCCATACCAGGCCAG	-
	TCAATGCTGGCAGTGGCGGCAAAAGAACGTGCTGCGCTGCCCGTGTTTCGCATATGAATATCCTCCTTAG	TGAACCGCCACTTAATGCCCA	-
y0095	ATGGCAGAAAAAGAAAATACGAAAAGGAACAGGCGCGAGGAAATATTGCAGTGTAGGCTGGAGCTGCTTC	GCGTATCGCTCAGATGGTAT	-
	TTATTGCAACTGGGCGACAATCAATGGCCAGCGGGAATCAAACTCTTGTGCATATGAATATCCTCCTTAG	GTCATTGCTATCGTGACGCT	-
y0177-y0179	ATGAGTTTGCTTCCGGTGATGGTGATTTTTGGCCTCTCTTTTCCACCGATGTGTAGGCTGGAGCTGCTTC	GCTAATATACTGTTGTCGGG	-
	TTAAATCAGCGCACTCTGATATTTACGCAACATCTCAGTCAACCGCTTAACATATGAATATCCTCCTTAG	GGCATAGTGTCAACCTCCTG	-
y0186-y0192	ATGCACGTCCAGGTACCCATCGATGATTTTTTTATAACCTGGGTGATCATGTGTAGGCTGGAGCTGCTTC	CCACTCGGGCGAGAAATAAA	-
	TTAGTGCCAACAAAGCATCGGTATATTAGTTAACCCTATTTTTGCCAATGCATATGAATATCCTCCTTAG	AATCACTCACGCGGTGAAAG	-
y0236	GTGTCAGAAGATATGTATCGCTATCGAGTTATGTCCTTATGCGTTACACAGTGTAGGCTGGAGCTGCTTC	ATGAGTGTCAGGGCCATATG	-
	TTAATACAGCGAAGGTTCGCCAATGGGTCGGGTTTTAAAGCGGCGGTGTACATATGAATATCCTCCTTAG	AATCGATCAGGGCTGGTTTG	-
y0260-y0263	TTGCATCGTATTACGATATCTAATAGCATCGCTATACGATGTGTATTCGTGTGTAGGCTGGAGCTGCTTC	AGAGTTCGTCTGCTGCGTTA	-
	CCTTTGAAGAATTTATCAATGGATTGGTCGAAGAAGACGATTTAGAGTAACATATGAATATCCTCCTTAG	CTTGCATGCGCTGTCTGAAA	-
y0329-y0332	ATGGAAATATTGTTAGAAGTCCGTGGCTTATCTGTTGAGTTTCCTGGTGTGTGTAGGCTGGAGCTGCTTC	TGCTCAAGAAAGACGGTACC	-
	GTGGATACCGAATTACTGAAAACCTTTTTAGAGGTCAGTAGAACTCGTCACATATGAATATCCTCCTTAG	ATTGAAAGCGTAGCCATGGC	-
y0350-y0352	ATGAAAAAAATTACTTTAGCTATCGCGTTATTCTCGGCATCTACAACGGTGTGTAGGCTGGAGCTGCTTC	CTACACGACTCGGTTACCTT	-
	TTAGCGGCGGCAACTCGCGGTTATATGAATGATATCTTGCTCGGTATCCTCATATGAATATCCTCCTTAG	CGCATTGGTGCTGAGATCAA	-
y0356-y0359	ATGAGCAAAACACACTTGACTGAACAGAAGTTTTCCGACTTCGCCCTGCAGTGTAGGCTGGAGCTGCTTC	AATCATCTTGCACGGACCAC	-
	GTGGCTTTCTGGTACAATTCGCTTCCCCGTAAACCCATTGAGCAACCGAGCATATGAATATCCTCCTTAG	TAGCCCCGTTATTCAACTGG	-
y0404-406	ATGACGAACAGTTCTCCTTATCTAGAAACGGATGTCATCATTATCGGTGGGTGTAGGCTGGAGCTGCTTC	AATCGGCGGCAATTTTGGG	-
	TCAAGCCAATGCCCGCGCCAGTAAAGTAATCGGGTGCTCACATTTCTTACCATATGAATATCCTCCTTAG	CGGTTCACTCGCGCTCTGTT	-
y0404-y0406	ATGACGAACAGTTCTCCTTATCTAGAAACGGATGTCATCATTATCGGTGGGTGTAGGCTGGAGCTGCTTC	GGGGGATGGCGAAATTGTTA	-
	TCAAGCCAATGCCCGCGCCAGTAAAGTAATCGGGTGCTCACATTTCTTACCATATGAATATCCTCCTTAG	GACATTGCAGATTACGCTCC	-
y0421	ATGAACCTCGGCGCTATATCCACTTTATTACTATGGCTTCTCGCGATGAAGTGTAGGCTGGAGCTGCTTC	GAAACAAGCGGTGTAAACAG	-
	TTACCGCCGCCAGCCTTGTGCTTCATGGCTAAATCCACACGCGAGCATAACATATGAATATCCTCCTTAG	TCCGGCACGATAAAGAAGGG	-
y0434-y0439	ATGTTTAATAATTCCATTCATAAAGTGTCGATCTGTATCGCGCTAACACTGTGTAGGCTGGAGCTGCTTC	AGTATTAATTGTTAGCGGTC	-
	TCAGTGGCGTGGTGACGGCGGCCAGTACCGTTGAATCACCAGGCTGGCGGCATATGAATATCCTCCTTAG	GAGTGCGAATTATTCAG	-
y0440-y0441	ATGATCGAACTGAAACACTTACGCACCCTGCAAGCTTTGCGTAATACTGGGTGTAGGCTGGAGCTGCTTC	CTGGCACTAAATCAACACCCGC	-
	ACGCTTCAGTGACTGGTTGGCGCTGTTCTCCATCACGGACGGCAGCGTATCATATGAATATCCTCCTTAG	CTGCTTCCGCTACAATTGCATT	-
y0508-y0510	ATGACGATGAGCCAAATACATAAACATCCTATTCCAGCTGCAATTGCAGAGTGTAGGCTGGAGCTGCTTC	TATCAACCTCCAAAATGCGG	-
	TCAGTGAGCGCCGCCTTTAGATGCCCCCAAGCCGGTTTGTGAACGGACAACATATGAATATCCTCCTTAG	CTATTCCCTGCGCAAGCAGT	-
y0511	ATGAAAAGTATAAAAATAAGTCTGGCATGGCAAATACTCATTGCACTGGTGTGTAGGCTGGAGCTGCTTC	CCTGGCGAAGAGACTATTTG	-
	TTAGCTTTGATTAGCTGGCGTCTGCTCTACCGCAAACAACGTTTTTCATCATATGAATATCCTCCTTAG	GTCGAAACACATCGGCTGAA	-
y0534-y0535	ATGAGCTGGCCCGAGTTTAAATCTCAATATCTGGTGCGCTTTTGGGCACCGTGTAGGCTGGAGCTGCTTC	GATGATCCACCGCGCTAGCT	-
	TCAGCGCTGAATCAAATAGCGAATAGTTGGCCCATCCTGCTGAATATCCACATATGAATATCCTCCTTAG	TTTCCGCGCCAGACAAAGC	-
y0539-y0547	ATGAATATTGATCTGATGCCGTATTATGCCCAACCAGGGCCACTGCCATTGTGTAGGCTGGAGCTGCTTC	TCACTCTGATGGGTGGGGCT	-
	TTATTGCCGCAGATAAATCTGTGGCAGGGCGCTTTCTGGGTGACGCGAAACATATGAATATCCTCCTTAG	TGCTTCAGTTTTGCGGGTGA	-
y0555-0557	GAGTTGAATGATGATGGCTTCATGATATGATTGGTGTGATGCGGTTAATGGTGTAGGCTGGAGCTGCTTC	GATAATCCCCCCATGAACCA	-
	TTACAGTTGAAAATCAGCAGGGACAAAACCTAACGTCACGCAAATTTCACCATATGAATATCCTCCTTAG	AAACAAACTGGCTGCCTCAG	-
y0575	ATGTTAGAAATTTTCTTTGTGATCGGTTTTTTTATGGTGCTTATGGTCACGTGTAGGCTGGAGCTGCTTC	ATTTTTACGGCCGCATTGGC	-
	TCAGTAACGCCGTATCACCGGTTTTTGCATTGACCGCCAAATCCAAGCCACATATGAATATCCTCCTTAG	TGCATATGTAGGGGTACAGC	-
y0602-y0605	ATGATAGCCGTAGAATTAGTCATCGTTCTGCTGGCCATTTTTCTTGGGGCGTGTAGGCTGGAGCTGCTTC	AAAATCTGTGCGGAAACCGG	-
	TTATTTTGTAACCGCGGGGGTGTTTTGTAAATGCTGTAAGAATTGCGCAGCATATGAATATCCTCCTTAG	GATCAAACTGCGGCAGAGCA	-
y0700	ATGGACGATTCTATGATTGATAACACCGGTTCTGTAGTCGATAAAAATACGTGTAGGCTGGAGCTGCTTC	TGCTGCCAGAGTAATAGCCT	-
	TTACCGGCGCGGGGTATGCATCAGATCTTGCTGTAAGCTGGCAACGGTTTCATATGAATATCCTCCTTAG	TAAAAACCAGCCTTCGGGCT	-
y0705-y0706	ATGGAGCTGCTGTGTCCTGCGGGTAACTTACCTGCATTAAAGGCCGCAATGTGTAGGCTGGAGCTGCTTC	AGTTTTCCCAACACAGGCGG	-
	TTACGACACCAGTTCTAACCCGGCAACACGATGCCAGTAGCCGTTACAATCATATGAATATCCTCCTTAG	GGGGTATCTGACGTCGCCAA	-
y0732-y0733	GTGAAAACAGTAGTGATTAAACGGGACGGCTGCCAGGTACCTTTTGATGAGTGTAGGCTGGAGCTGCTTC	TGATGTGCTGCGGGTTGCCT	-
	TTAACGTAAACAGTGAATAACCTGATTGCTGCTGCCGCGCCAGATAAGTGCATATGAATATCCTCCTTAG	GGGTTTGGGCTAACGTCGTG	-
y0773	ATGAACCGCCTCCCAACCCTGTATGGCATTATTGCGATTTTACTGTGGAGGTGTAGGCTGGAGCTGCTTC	GTCCCTAACACATCAGGTCA	-
	TCAAGTACGAAGGGGATTTCGGCTGGCTGACCAGCATAGCAACGAACCTGCATATGAATATCCTCCTTAG	TGAAGAAGTGATGGTGCGTG	-
y0857-y0859	ATGACTCATATTTCTGATGTTGATAGAGACGTGCCGCTTATCACGTTAAAGTGTAGGCTGGAGCTGCTTC	AATCAGCGAGGGAGATAAGC	-
	TCACTTGGCGTGACATTTTTTTAGATAATTCAAACGGTTCTGCCTCATGGCATATGAATATCCTCCTTAG	AGCTTTTCGACCCAACTGGT	-
y0864-0867	ATGAAAGAGACCAACGAATCCCGGCTGTTAACGACAGGTGTCACCCGCCGGTGTAGGCTGGAGCTGCTTC	ACTCCCCCCTCCTCGAAAGA	-
	TTAGCGATATAACTCAACCCCAGCCGGTGTCACTTGCAATTCATGTTGCCCATATGAATATCCTCCTTAG	CGCCTTGGCGTTTCTGAAGA	-
y0915	ATGCGGCTGCATATCTATCTTCGCCGATTGCTTATCGCCCCATTGCTCTTGTGTAGGCTGGAGCTGCTTC	ATGGCGCTTGGATTTGGAGA	-
	TCAATTCGCTGCCCGATAAAGACTATAACCAGTAATAGCGCCAGCGATATCATATGAATATCCTCCTTAG	GTATTGATAACCCCATGCCC	-
y0932-y0935	ATGAACATAAAAAAGCGGATGTTGTGGCTGTGGGCAGTACTGGTATTGGTGTGTAGGCTGGAGCTGCTTC	CACCTTTCAGAGCACGTCAT	-
	TCAGGCAGGGCCAGATAAACCGCGGACTGACGATATAAACTGTTTCAATTCATATGAATATCCTCCTTAG	CGATCAACATTGTCCAGCGA	-
y0948-y0949	ATGTGCGAATTGCTTGGGATGAGCGCAAATGTGCCCACGGATATTTGCTTGTGTAGGCTGGAGCTGCTTC	CGGTTATGCCGATCGTATTC	-
	GTGGCAGTATCCAAAGAGCTAAAACAGCAACTACTGGGGTCATCGGTTTACATATGAATATCCTCCTTAG	CTGACTGATAATCTGCGCCT	-
y0982	ATGGAATTACCAGTTAAAAATAAGCTTAAACGTGGCCTGACGACACGTCAGTGTAGGCTGGAGCTGCTTC	TCTTGATGCCGTGAAAGCGT	-
	TTAATCAGCATTCTTGATCAGCGTAGCCTGTTTTTTCTGATACTTGACCTCATATGAATATCCTCCTTAG	GAGTAACGAACATAGCCAGC	-
y1036	ATGTACCGTTTAGCCGCTTTTGATATGGATGGAACGTTGCTGATGCGTGAGTGTAGGCTGGAGCTGCTTC	TAGTTGCTGAATCAGGGTGC	-
	TCAGAATTCGGGGGAATAGGTGAGGTGTGGTGAACTTAGCCAATGGTGCACATATGAATATCCTCCTTAG	GGTCAGCAATAACATTGGGG	-
y1043	ATGAACTCAACCATCAGGGGCGACAACATGAGTCAGGCATTAGAAAAACTGTGTAGGCTGGAGCTGCTTC	TAATGCCTTTGGCTGCCAAG	-
	TCAACGATGTAAACGAATCACACCCTCCTGCACAGTAGAAGCTACCAGCACATATGAATATCCTCCTTAG	GTTAGTTGTTAGCCTGTCGG	-
y1067-y1068	ATGTCATTAAATGTTAAGCTGCATCCATCAGGTATTATTTTTACTTCCGAGTGTAGGCTGGAGCTGCTTC	AGGCCCTGTTATGGTTGTAG	-
	TTATTCCCATACCTTCCAAGGTGCCCTGCCTTCTTCCCATAGTTCATGCACATATGAATATCCTCCTTAG	TCTGGTTGGAATTCGCGGAT	-
y1076-y1077	GTGATATTAAGAAAGTTTTTTATTATTTGTGATACAGTCTATCCCGATGAGTGTAGGCTGGAGCTGCTTC	TTGCTTGGAGAAAAGGTGGG	-
	TTATCTTATGGCAACTGATTTAATTTTCACCCTAGCTATCCCAAATAAAACATATGAATATCCTCCTTAG	GACAGTTACCCCTCCCATAT	-
y1113-y1114	TTGCGAAAATATGATCGAACAGGTGCAGAAAAAACTTCGCTCATTGCGTAGTGTAGGCTGGAGCTGCTTC	GCTGGTAGCATTGAATGCAG	-
	TTATTTCGATGAACGTAAAATCAATGTGTTGTCTCTTACTGCAACGACTCCATATGAATATCCTCCTTAG	TAACATGCCGTCGATGGCAT	-
y1115-y1120	GTGACATTGTATGACATTAAACCTCAGTTTCAGAATCTGCTGCGACCGCTGTGTAGGCTGGAGCTGCTTC	GACACCGACCTGTTGACGCA	-
	TTATAGCTCCAGGATCTCTTGAATCCAATCATCCCAGCACTCGCTGCCAACATATGAATATCCTCCTTAG	TGGCGCCAATCCGTATTGAT	-
y1140	ATGTTTTTCAGAGCATGGGATAATTACCCGAACACATCAGATGGAGGGGTGTGTAGGCTGGAGCTGCTTC	TCCATCGCAGCCGAAATTAG	-
	TCAGCCTGGATTAACTGACTCTGCGGGTGATTTTTTAGGTTTTAGATAACCATATGAATATCCTCCTTAG	CGCGCTGATAGTAGGCTAAT	-
y1147	ATGACCATGGCACAGGCGCTGGTCAGGTTTCTTAATCAGCAATATGTGGCGTGTAGGCTGGAGCTGCTTC	GAATATGGTAATGGCACGGC	-
	TTAGTACTGACGGGCACGTTTTACCTTCATCTGGGTTCGCTCTGCAGCCTCATATGAATATCCTCCTTAG	GATATCTCGGTGGTCTGGTA	-
y1150-y1155	ATGTCGCAACCTTTATTGAAAATCACCGATATGGCGAAAAGCTTCTCTGGGTGTAGGCTGGAGCTGCTTC	ATGGTGACAGGTTTCGATGC	-
	TTATGTAATAGGATAATTATGATTTATCCAAACATGATCGTTTTCCCAGCCATATGAATATCCTCCTTAG	CAGCGACAACAGGCCAATAT	-
y1160	ATGTCAACATTAGAGAAGCTGGTTTCTGCATATTGCCATACCAGTTTGGAGTGTAGGCTGGAGCTGCTTC	TCATACTCGCCGCTGTGGTG	-
	CTACAACGGCCTGTAAATATCCCTGTAGTACGTTAATCTTTTACGAAAAACATATGAATATCCTCCTTAG	AGTACCGTTCGTGAAGCCCG	-
y1161	GTTGTTTAAAACATAAAGGTGTCGCCATGAAAACCAGCCATCGGGCAATCGTGTAGGCTGGAGCTGCTTC	GTGGTTAATCATACTCGCCG	-
	CTACACTGATATTAAATAAGCCACCTGCGTGATGTGAACCTCTATCGTGGCATATGAATATCCTCCTTAG	CGTATTTGCCGCCTCAGTTT	-
y1169-y1170	ATGCGCCAAAATGCTAAACAGACTCGTGAACTCATCATCGTTCACTTACGGTGTAGGCTGGAGCTGCTTC	AGCCTTGTTAGGGCGGTTAT	-
	TCAGTCTTTGCGCTCAACCGAAGCTGCGGCTTGGGAGTCAGATGCCGCTTCATATGAATATCCTCCTTAG	AAGCAGAAGCCATGGCTATG	-
y1172-y1173	ATGATGCCTCGCTTGCAACAACACAAGATCATTTTACGTCAGCTAGGGTTGTGTAGGCTGGAGCTGCTTC	GGTCAAGATGGTGCTCTAGT	-
	TTACAACACGCTTGATGATGAATGGCTTGATAATAAATAGTCTGATAATGCATATGAATATCCTCCTTAG	CGCCATTTTGTCTGCATCAC	-
y1251-y1252	GTGTCTTTTGAACATCAAAAGGTGTTGGGTATGGAAAAGAAAAAAATTTAGTGTAGGCTGGAGCTGCTTC	GCCATTTTGCGATAACCATC	-
	TTAGAATTTCAGGCTGTTAGCAATATCGTCTTCGCTTTCGACAGTACGGTCATATGAATATCCTCCTTAG	TGGCCCGCGTTGATGATAAG	-
y1265-y1267	TCAGTAAAAGTCACGACCTGCCTTTTCTGATTGGGTCAGCCAAACCGGTTGTGTAGGCTGGAGCTGCTTC	TAGTTGCCAAGAATCTGCGG	-
	TCAATGTGCTAACCAGACTTGTAGATGTTCAGCGACCGGCCCCAGGGCCACATATGAATATCCTCCTTAG	GCCCACTTAATATGTCCAGC	-
y1282	TTGGTTTTACCAAATAACCAAGATAATTATCTTCCCTTGGAATTTATAGCGTGTAGGCTGGAGCTGCTTC	ACACTCTCCTTGGTTTCTGC	-
	TTACAAACTTTCAGTGAAAGTCCGGGTAATCACATCGCGTTGCTGTTCTGCATATGAATATCCTCCTTAG	GGCGTCTTCTTTCTTTGCCT	-
y1288	TTGGTGACTAATGTGGGCGAACAATTAAAAAAACAACCGGTATTACGTGGGTGTAGGCTGGAGCTGCTTC	GGTTACAGTCATGGTCTTCC	-
	TTAATAGTTTAGATAGAAATCACTGATGAGCTGGCAAAACTCAGGTGAGTCATATGAATATCCTCCTTAG	CCATAAGATTGAGGGCGTCT	-
y1323-y1330	ATGCCTCCCCCCGCGAAGAGCCGGCACTGCAACATTATTTTGACATTTACGTGTAGGCTGGAGCTGCTTC	TATTGGTAGCCCAGCGATCA	-
	TCAGCGACCAAATGGACGAACAGCCCCAACCATCTCCGGCTGACTGGACACATATGAATATCCTCCTTAG	GCTATTGGTTCGTGAGTCTG	-
y1359-y1361	GTGGATGCATTATGTCCGGTTTGCCAGAAGGCGATGACTGAGGTGAGTGGGTGTAGGCTGGAGCTGCTTC	TGGCTTACTGATGGGGTTTC	-

[illegible]	ATGTCGCAATCTTCCGCCTCCTCAATTTTTACTGTCAGCCGCCTGAATCACATATGAATATCCTCCTTAG	TGCCAAGTTCTGCCGTGTTA	-
y1390-y1393	ATGGCATACCAGCAGGAGCGAATCATGAGTCACCGTGAATTGGAACAACGGTGTAGGCTGGAGCTGCTTC	ATAGATAAACGCAGCGCACG	-
	TTATGGGGACACTGGATGATGGCGTAAACGCTCACTACGCCGCCGGAGCACATATGAATATCCTCCTTAG	CCTCTCAGTGAGTTATCTCG	-
y1442-y1447	ATGACTGATTTATCGCGGCGTAAATTGCTGACGGGTTTTTGGCAAACCGGGTGTAGGCTGGAGCTGCTTC	GATTTAACTGGGCAAAACCC	-
	CTAAAAACCAATAGGTACATCTTTCATATCCGGCAACTTATGGGCAATCCCATATGAATATCCTCCTTAG	GTACCGAGGTTCCCTCATCT	-
y1453-y1454	TTGAACACCAATAGTAAAAAAACCTACCTTAACATTGCTATTTCATTCATGTGTAGGCTGGAGCTGCTTC	AATCCGCATGCGTTCCCATA	-
	ATGAACTTAAAAAGTAAAATCTTTATTTTCTGGGGGATGATGGATCTCTACATATGAATATCCTCCTTAG	TTTGCCACACTCTTGCCACA	-
y1474	TTGGCCGTTTTACAAATTTTTGACAACTTTATACAGCAAACAGACAACTTGTGTAGGCTGGAGCTGCTTC	CAACCTCACCATCCTTTTCC	-
	TTACTTCTGCTGCTGTTTGACGTTGTAGTTCTGTTCTTTCAGCTCTTTACCATATGAATATCCTCCTTAG	GAAACAGGCTTTTTGACGGC	-
y1524-y1526	ATGAAAATACCTATACCCTCTATTGGCCATGTTGCTTTACTGGCGTCTGCGTGTAGGCTGGAGCTGCTTC	GGAAAATGCCGTTAAAGCCG	-
	CTACTCGGCAAAAATATGTGCCTGGCCCATGATATCCAGGCCAACAGTTACATATGAATATCCTCCTTAG	TCCAAGAACTGAGGCTCCGC	-
y1554-y1556	ATGTCAAGGACAATAAATTTGAGCAGCCCGGCTATGCCTTATCTATTGGGGTGTAGGCTGGAGCTGCTTC	TATTTACCCGCTTCGCGACT	-
	ATGCTCTACGTGTGTTTCCCACGCAGAACATGCTTACCTTTTATCTATAACATATGAATATCCTCCTTAG	CGTCTTATCACTACAACGCG	-
y1561-y1562	ATGGAAAAGAAAAAACAGTTTTTACCGGCGTTGTTAGAGCGTTTACTCGAGTGTAGGCTGGAGCTGCTTC	AATGGCATCAACCGACTGAC	-
	TCAGCGCGCTCCGGACATAACATCGATAATAACGATTATAGAAAGTAGGCCATATGAATATCCTCCTTAG	ATCTGCTGGCTGATGGTGAT	-
y1567-y1576	ATGCTGGAAGCAAAAAATCTGACCTGTATCCGCGATGATCGCTGTTTATTGTGTAGGCTGGAGCTGCTTC	TCGGTTTTCCTGAACTGGCC	-
	TCAATCGGCGGCATCTATGCTATCCAGATCGCCCTGAATAGCCTGAGCATCATATGAATATCCTCCTTAG	CGGCCCCATCTAACACCACA	-
y1595	ATGAATGTTTTATTTCCATTGTTTGCGGTGATTATTTGGTCAGTCAATGCGTGTAGGCTGGAGCTGCTTC	GGATCCTTGAGGTGACTACA	-
	CTATCTACTGTTTTTGTTGTTTGGGATATGTCCAGCAAGATCTAAATTGCCATATGAATATCCTCCTTAG	AGGAAATAAGGGAAACCCGG	-
y1608-y1610	ATGCTGGATGGATATTCCAAACTGATATTTGAAGGTGCGCTGGTGACACTGTGTAGGCTGGAGCTGCTTC	ATTCTTCGGTGTAGGGACTG	-
	TTATTTCAGTGAGCCAGAAAGAAACTGCTGTAAACGTGGGCTTTTAGGGTCATATGAATATCCTCCTTAG	GGGTGAATCCGCAATATTGG	-
y1626 (Sab)	TTGAACAGCGAACTACTGTGGGTGTTGACGCTACTGCTGATAGCTATTGTGTGTAGGCTGGAGCTGCTTC	GCCACTTCTCATGGTGCGGT	-
	TTAGCATTTATTTCTGGTAATATTTTCTCTCAGCACGTTTATAGGATCCACATATGAATATCCTCCTTAG	CGCCAAAGCCCAGAGATGGA	-
y1629	ATGACAAATGCAAATCGTCCGATAATTAATCTCGACCTCGATCTGCTTAGGTGTAGGCTGGAGCTGCTTC	CCATCAAACGGTAGGGCTAA	-
	TTAATCTTCATCACCTGTCAAATAATCGGAATCCAATATTAATGAAGATTCATATGAATATCCTCCTTAG	GATCCAGGACACGTTTTGAG	-
y1651	ATGAAACACAGCAAACAAGCCGTATTGATAATCGCTCCGGTAATGGACTAGTGTAGGCTGGAGCTGCTTC	AACTGGTCACGTTACCGGTA	-
	TTAATAGGTAATTGCTGTAGGTGCCGTTTCGCCAGAGAAATGGGCCAAAACATATGAATATCCTCCTTAG	TGACGCGCTATTACTCATGG	-
y1659	GTGGTCGTGAAACAACTTTCTGGTTTGCTGGGTGAATTACGACAGAAATTGTGTAGGCTGGAGCTGCTTC	AATGCCGCTTGCCAGATGAT	-
	TTAGGCTTTAACGGGCTGCGCTTCGTGCTCCAACAAGCTTTGTAAACCTGCATATGAATATCCTCCTTAG	GCCCAACGACGGTTAAAAAC	-
y1660-y1664	ATGTCGACAAGTGTTTTTAACCGTCGTTGGGCGGCACTGCTACTGGAGGCGTGTAGGCTGGAGCTGCTTC	AATGCCGCAATTAACCGCAC	-
	CTATCTTTCGCCAATCTTCTCCCCTTCATACTTGCCGCGCAGCCAGTCGGCATATGAATATCCTCCTTAG	GCCTGGTAAAAGCCCAGAAT	-
y1666-y1667	ATGAAAAACATCAAAAATTTCGTTGCAGTTATCGCATTGTCTACTCTTTCGTGTAGGCTGGAGCTGCTTC	TTGACACCACCAGCCCCTTT	-
	ATGGGCTTTTGTATTTCCAAGGTGTTGGCAGCTATTTATTGCATCACTGACATATGAATATCCTCCTTAG	ACCACCAGAAAAAGCCCATC	-
y1673-y1675	ATGAAGTCACTTGTCAAAGTTTCATTGGCCGCACTGGCGCTGGCCTTTGCGTGTAGGCTGGAGCTGCTTC	GCAAGCTAAGTGATTGGGGT	-
	TCAGGAAACGTGTTGCAGGAATTCCCGTAAACGTTCGCTGGGCGGATTGGCATATGAATATCCTCCTTAG	ACGAGCGGTGGCTATTTCTA	-
y1696-y1699	TTGCGTTATTGTTATTTTATATTACACACGGCTATATTATTTCCACTGTTGTGTAGGCTGGAGCTGCTTC	GTAATCCGAAAGGGAGAATG	-
	ATGGACGTATTCGGTACTGCAACAGGTGGCGGAACCGGTATTGGCACCGCCATATGAATATCCTCCTTAG	CGCTGATGTGATCCGATCAT	-
y1700	ATGTGGGTGGTGCTGGATCGGGTAACTCCTGGCGGTCATGGGCCGGAAGAGTGTAGGCTGGAGCTGCTTC	AGTAAGATCCCGACACCGAT	-
	CTAAGCCTGATTGCCCTTCAGAGGCTCGAATGTTCCTCCTGGCCTACTGGCATATGAATATCCTCCTTAG	GCCCAACCGTATAAGATAGC	-
y1737	TTGCACAATACCCTATCTAACAAGGCGGGTGGCAGGGATAGATTGAGCTGGTGTAGGCTGGAGCTGCTTC	TTGGCGCATTCCTCCTTGTT	-
	TTACTTGCAACGATAATCGTGATCAGTAAAACCAGAGGTAATGAGGCCGGCATATGAATATCCTCCTTAG	TGATTGCCCAAAAGAACGGG	-
y1783-y1784	ATGGGCTGCACAGTTAAGCGAGTGGCATTAGCATTAACGCGGGTTTCTCAGTGTAGGCTGGAGCTGCTTC	TACCACAACCCAACAGCGTT	-
	ATGTCCCAACAATATCGTTATTCATTGCCTGAACGACGTGGTGATACCCGCATATGAATATCCTCCTTAG	CGATAAATAGGGCGACAGGT	-
y1791	ATGGACAAATTACTCGACCGATTTTTCAACTATGTTTCTTTTGATACACAGTGTAGGCTGGAGCTGCTTC	GTCAATCAACACGTCCCGGC	-
	TTATGATTCTTTAGCCCGCTTGGCGGTCAGCTCAGAAATTCGCATAATGACATATGAATATCCTCCTTAG	CTCAAACGCCCCTTTTCGG	-
y1806-y1807	TCATACTGACCGCTGCTGCCGTTGTTCCATAATCCGATGCAAGTTTGTCTGTGTAGGCTGGAGCTGCTTC	ATTGGCGCGCTGCTGAAAAT	-
	TTAACGTGTCGCCAGTGTTGCTGTTATTACCTTGGCATAAGGGGTGGTTGCATATGAATATCCTCCTTAG	TCTGCACCTGTCTACCATGA	-
y1820	ATGGAGAGAATCATGTCATTAACTCCTTTTGTTATGAATTTATTACTGGCGTGTAGGCTGGAGCTGCTTC	CAGGCACTAACACCGGTTAT	-
	TCAGGCCGGTAATTCAGATGCAATGCGCCAACGTATGGCACTGACACTACCATATGAATATCCTCCTTAG	CTTGGGGATTTACTCGGTTG	-
y1823-y1827	GTGTTAGTTATTTTGGGTTATCTCGTGGTCTTAGGTGCGGTTTTCGGTGGGTGTAGGCTGGAGCTGCTTC	TCCGCAGTCTGCCGATATAA	-
	CTATGCGGTAACCACCTCATCAGCCGCGACGCGTTTTTCACGGTTTAAGGCATATGAATATCCTCCTTAG	GCCTCGGATCTCTTGTACTT	-
y1835-y1836	ATGGAGTATTGGCTGTTAATTTTTTTACTTATCTTCTTGTTTGTCATCTAGTGTAGGCTGGAGCTGCTTC	AACTTTTCTACACTGCCGCC	-
	CTAATTCAATGAGACCACACCATAGAAAGCGGCCACGGCAGCAGCACTCACATATGAATATCCTCCTTAG	AGCAGGCTGGTAACCACTTT	-
y1846-y1847	ATGAAAAGTATCCAAAAGTGTGACTGTAATTATCTGGTCAGATTTGCAGTGTGTAGGCTGGAGCTGCTTC	TGGTTGTACTGCTGCCTCTT	-
	TTACCATTGATAACCGACGCCGACACCAACCCCCATATCGCCTTGTGTATCATATGAATATCCTCCTTAG	ACCCTGTGCGGCTCTATTTT	-
y1849	ATGCACGTTCGTTTTCGTCATTTATTCTTATTACCATTAATAACTTTGGTGTGTAGGCTGGAGCTGCTTC	AACTTAACGCCATGTAGCGC	-
	TTAATCTACCAGAATACGCTTATTGGTTGCGCCCCACAGAATTGACATTTCATATGAATATCCTCCTTAG	GGTGTAAGCCTCTTAACCTC	-
y1860-y1862	ATGAGGAAAACCTTATTTACTTTAAGTGTAATAGCCATATTACAGACAGCGTGTAGGCTGGAGCTGCTTC	ATTGCGTCTTGTTGGCGT	-
	TCAGTAAGTGACAGTAACCAATAACGTATCTAGATAAACTCCCGCAGTAGCATATGAATATCCTCCTTAG	ATGGCAGTCTGAGCGTTGTA	-
y1880	ATGAAATTAAAATTAATTACTCTGGCAGTGGCAGCAGCAGCAGTATCCGTGTGTAGGCTGGAGCTGCTTC	CTGCCATTTATTTGGTGGGG	-
	TTAGAAACTGTATTGGATACCTACGCGGTAACGAGTTTGGCGTTCGTCAGCATATGAATATCCTCCTTAG	CGGTGGCTGCAATATCACAT	-
y1898	GTGAATTCAAAAGTCGGTTGCCTAATGGCAATTTTATTGTTGGCGGGGTGGTGTAGGCTGGAGCTGCTTC	TTGTAAACAAGGCTGCAGCC	-
	TTACTCTGACATCGCCCGGTAAGCGGTATCCACCTTCCACAAGTAACGTGCATATGAATATCCTCCTTAG	ACTCAGCTCAGGAGCGATTA	-
y1901	GTGGTGATGAGTCACTCAGGTCAAGCCATTGATAAAAAATCATGTCTACCGTGTAGGCTGGAGCTGCTTC	TGCGATAGATCATGCCTCTG	-
	TCATTTAGCCAGAAAATAAAACGCCCCGATTGAATAAACAATCAGGGCGACATATGAATATCCTCCTTAG	AGCTTGGGTGTTGGATATGC	-
y1959	ATGAACAAAATACAGCATACACATGTAGACACCCGAGAACATCTACTCGCGTGTAGGCTGGAGCTGCTTC	CAAAAATGCCGGAGTCTGCA	-
	TTAGGCTGGTTGTAATATGAGTTCAATGCTTCCCAGTGCACAAGTCAGTGCATATGAATATCCTCCTTAG	TGGCCCGTTGGCGATAATAT	-
y1980	ATGGGTATTTTTTCTCGCTTTGCCGACATCGTGAACGCGAACATCAATACGTGTAGGCTGGAGCTGCTTC	CGAGCAGATTATCCAGTTGC	-
	CTATTCAACTGATTTCATTTTCGCTTTTAATGCCGCTAATTGGCTACTGACATATGAATATCCTCCTTAG	AACGCCATTTTGCTGACGAC	-
y2044	ATGAAAAAAATCACTTTGGCACTGCTCGCCGTGGCCTCGTTAGTACCTACGTGTAGGCTGGAGCTGCTTC	TTCTAATCGACCGGTTGCCC	-
	TTAAAAACGATAACCTGCGCCAAACATAAATACCCATGGGGCCAAACGGGCATATGAATATCCTCCTTAG	CGCTCGGTACCGGGTGTAAA	-
y2068	ATGATGAGTGAGATATCTACCTTAACCATTAAAATTCCTCTGGAATTAAAGTGTAGGCTGGAGCTGCTTC	AAGACAATAAGCCGTTGCCA	-
	TTATTTTTTCTTTTGAGCGGTTTTCGCACTGTCTTTCAGCAGTTGACGCACATATGAATATCCTCCTTAG	GGGACATGGTTGGTGAGCAA	-
y2085	ATGTTTAAGCTGTTTACTGCCCGCCAACATGACAAGATCTGGGATTTCGAGTGTAGGCTGGAGCTGCTTC	AATGTATTCAGGCCTGTCCG	-
	TTAGTCAGGATTTATCGCTTGAGCGGCTTTCTTTGCTTTGACTCTGGCGACATATGAATATCCTCCTTAG	ACGTGATGAAGTTGGTCGTG	-
y2097-y2098	GTGCAACCCAAACTTTATAAGGAAGCCATCATGTCAGCACCCCATTCTGCGTGTAGGCTGGAGCTGCTTC	ATCAACGGAGTTGATGCCGT	-
	TTATATCGTCCGTTTAGCTAACTGTTTGTAACGGTCGAAGATAACCGCCGCATATGAATATCCTCCTTAG	TGGCAGCAAAGGATTGGGTT	-
y2111	ATGTTAACGCGTTTATTTGTGACAGGTACCGACACTGCTGTTGGTAAAACGTGTAGGCTGGAGCTGCTTC	GCGCTAACGCAAGCTGTCTG	-
	TTATCCTGAAATAGCTGTAAGATCTAAATATTTTGCTAATGGTTTTTCTTCATATGAATATCCTCCTTAG	GGTTTGTGAAATGACCGGCG	-
y2112	TTGATGTTGTCTTGCATGAAACGGCCAATCCATCTCTATTACTACCGTGCGTGTAGGCTGGAGCTGCTTC	GATACCGATACAGGCCGTAT	-
	TTAGTGAGACCGATATACCGATATTGGGCGCAACCAGCGGGCTATTGTTGCATATGAATATCCTCCTTAG	TAATCCTGGCTTGGCCCATT	-
y2121-y2123	GTGGCAAAATCATTTTTACGCAGTGGAAGTTTGGACGATATTCTGGCGTTGTGTAGGCTGGAGCTGCTTC	TCAATGGCCTCAACAACTGC	-
	TTAATCATTTGCGGGTTTTATCACCGATAATATTGCCGCCAGTTGTTGCCCATATGAATATCCTCCTTAG	ATGCGTCTTCGCTGAAAGTG	-
y2129	GTGGCGAAGAGAAAATCTACCGGTACCGGCTGGGTTATCCTACTGATAGTGTGTAGGCTGGAGCTGCTTC	ATGTATTGAGCGCCGCTACT	-
	TCAGGCCAATAATGCAGCCGAGACATCACGATTAATTGAAATAACCGCATCATATGAATATCCTCCTTAG	ATAATCGACCCACAGTGGCT	-
y2180-y2227	TCACTGTGATATGGCGCGGGGCATATACGGGGCATCACCCGTAAATCTTGGTGTAGGCTGGAGCTGCTTC	TTGCATCAAGTTAACGCGGG	-
	CTAGGCCTGGGTGTGCGTACAGGTATACTGGCTAGCCTGCACCTCATCAACATATGAATATCCTCCTTAG	TGCCCTGAGTGTGCCTTGGT	-
y2272	ATGGCTTTGGTATCGCAAGCTCGAAGCTTGGGTAAGTATTTTTTATTGTTGTGTAGGCTGGAGCTGCTTC	CTGTTATAGGGACATCGCGT	-
	TTAGCTGCTGCTCAACATGGCAGATTCAATCCGCCGTCGATTGAATTGCCCATATGAATATCCTCCTTAG	GGATCTACTTAAGCCACCCT	-
y2275	TTGGCACTGCTGATGAGAAGAATTCCTGCTATCATTAGCGGACTATATTTGTGTAGGCTGGAGCTGCTTC	CGCTTACGCGTATACACACT	-
	TTACAGCTCTGCTAGCTCGGGGATCGCTGGGCGACCACGCTGCTCCAGATCATATGAATATCCTCCTTAG	AGGTGAAACACTGCACTCTG	-
y2289-y2290	ATGAAGTCTTCTATTGTTGCCAAACTGGAAGCGTTACAAGAGCGCCATGAGTGTAGGCTGGAGCTGCTTC	AACGATCCGTGACTATCGCT	-
	TTACACGGCCCATTGCCCCAATGTCACCCGGTCATTATTGCCATAATCTTCATATGAATATCCTCCTTAG	ATGTTCCCTGCGGACTGAAT	-
y2296	ATGAAACGTAAGAGCGCAACAGTACTGGGTAATATGCTGATGGGGTTGGGGTGTAGGCTGGAGCTGCTTC	ACTGGAATACAGGAGAAGGG	-
	CTATGAGTGACGTTGATTCTTGGTACAACGCTTATCAAAGTGCTTAACCCCATATGAATATCCTCCTTAG	CCGTTGTCCGCTGAAATCAA	-
y2313-y2316	ATGTCGAAAGTTACTAGTCCTGATTCTATCAATAGAATAAAACATACGGAGTGTAGGCTGGAGCTGCTTC	GTTCTCGCTCAAACTCCGAT	-
	TTAGCGGGGTTTTGGACTCATGCATGGCATGCTTCCAATACTGTGAGAAACATATGAATATCCTCCTTAG	CCGGCTTGAGTCACTAAGTT	-
y2320-y2321	ATGCAAGGACGCGATTACATTCCCTGCGATATCGCAGAGTACCATCTGCTGTGTAGGCTGGAGCTGCTTC	AATAGATGACGTCGCCTTGC	-
	ATGACTGCGCTGAACATAGCATTTTGTGAACATCATTTCAATTTTAATGACATATGAATATCCTCCTTAG	CACAACCGGTCACCACAAAA	-
y2331-y2335	ATGTCGACATTATTAATTTCAGCACCTCATCTGGCGTTCAAAATTATCGCGTGTAGGCTGGAGCTGCTTC	ACGTCAGGATAGTGGCGGTA	-
	TTACGGTTGGCAAAGTGCAGGCGTCGGATACGAAAATAGTGCCAGAGCGTCATATGAATATCCTCCTTAG	TAACCCAACGCTGAACTTCC	-
y2340 – y2341	ATGATTCGGGTGACAAATCTGCCGAGTGCAGCTAACACCGCAGCAACGTCGTGTAGGCTGGAGCTGCTTC	TTGCTTGTTGATCCGCCGTA	-
	TCAACGGTAAGTTTGCTCACCCCACGTCAGCATGGTTGTGATGAACTGCTCATATGAATATCCTCCTTAG	TTCTGTGTCGGAAACAGGTG	-
y2350	TTGAATTTTATCTATAACAGATGGCTTATGACACGATATGAACAGCTAGCGTGTAGGCTGGAGCTGCTTC	CGCCGTTACCTTACCGTTTT	-
	CTATCGACGTTCGTTCGTCAATTGGCCGATTAACTTCGCCAAAATACGAACATATGAATATCCTCCTTAG	GAGAATGCCTCAATCAAGTG	-
y2362-y2370	ATGCGCATTTGGCAAAAATTCTTGCTGCTCTTCTGCTGTTGGGTGTTATCGTGTAGGCTGGAGCTGCTTC	TCTATCTTGAAGGGCGACGGG	-
	TTAGGGAGTTTTAGGTTTCGCGGTTTTCAGATGCTGAATGTGGTCAGCCACATATGAATATCCTCCTTAG	CAGAGGTGTTTTCCATCGTGA	-
y2412	ATGCAGAGCGTGGAGCTCAAACCAGATTTATGCAACCCGCAACTATTGGTGTGTAGGCTGGAGCTGCTTC	CGTGATGGGCTGGTCAATGA	-
	TCATTTTTTATCTTTACAATCTGCTAGCCTCAACTGCACGCTGTTACGTCCATATGAATATCCTCCTTAG	AAGCCGTTTTCAACCGCG	-

y2437	ATGGTGCAATACCGTTTGAAAAAATCTCGTTTATATATGAGTCAATATCCGTGTAGGCTGGAGCTGCTTC	CACGCCATAACAAGAGAGCA	-
	TTACTTGAATGCATGGGCGCGAAAATTGATTGGTTTAATAAACTCTGTCACATATGAATATCCTCCTTAG	CTATGCTGTTTTTCGCGCTC	-
y2455	ACAACGTATTGACCGCACACCACACTGGCTCATTAAACAGGCTATTTTCAGTGTAGGCTGGAGCTGCTTC	AACCCTGAATTCGCCTTACCAA	-
	TTGCACTTCAGTAGGCAATTGAGGCAACAGTGCTTTCTGTACGTCATTCTCATATGAATATCCTCCTTAG	GGGGGCTGATAAGTGAATCGGT	-
y2463	ATGTCAGGACTCCAGTTAAAAAAACGGCCGCTGAGCCGTTATTTAAAAGAGTGTAGGCTGGAGCTGCTTC	AGCGATAAAACGCTTACGCG	-
	TCAATAGTTCTTTTGCCATGCCAAATACTGATCATATTTACGCAAAGCAACATATGAATATCCTCCTTAG	CCCCCCAAGGAATTGATGAA	-
y2466-y2468	ATGGCAAGTATCAGTGCATTAGGTACCGGTTCAGGACTGGATTTAAATACGTGTAGGCTGGAGCTGCTTC	ATGATTTGGGCTTACTGCCC	-
	TTATTGTGTTTCACCCAGCAGCAGTGCCTGATCGGGGAACTGACCATAAGCATATGAATATCCTCCTTAG	CTTTGAATAGCAGGCTGGCA	-
y2475-y2490	CTACTCCAGCACCCCAATCACATTCAAATTCATATCATCTGGGATTTCGTGTGTAGGCTGGAGCTGCTTC	AAAGGGGAGGTTTGGCAAGC	-
	CTATTTTTCAGCTTTCTCAGCCGCAGGTTTCACGACATTCTCGCCGTGATCATATGAATATCCTCCTTAG	GGCAGCCAGCGAAAGAGAAA	-
y2538-y2546	ATGAAGATTGTTAACCATTGGATTGATGGGAAAAATATTACCAGCAATGAGTGTAGGCTGGAGCTGCTTC	TGGTTATTCGCACTTGGGTC	-
	TTACACCATCACCGGGTGAAAATGGCGTTTAAAGTAAATCAGACCATGGCCATATGAATATCCTCCTTAG	TGAAACAACCCAAAGGGGAG	-
y2556	ATGAATCAGTCTCTCTCCATAAGTACGGAGCCGAAACGTTCGGCTCCTCGGTGTAGGCTGGAGCTGCTTC	TGTCCTCCCTGCTTATCGCC	-
	TCAGAAGTCCATGGAGACGGATAATTTCAGCGTACGTGGATCGCCCTGATCATATGAATATCCTCCTTAG	CGCCGGGGGATTTCAGTAAA	-
y2566	ATGATTAAATGGTATGAAGAAAGTGATAGTGAAGTGAACCGAAGCATTGCGTGTAGGCTGGAGCTGCTTC	ACTACCAACAGGCAGTGTCA	-
	CTACACTTCCTGGCTGAGTAGATAACAGATCATCGCCGCGCGTAGCGGGCCATATGAATATCCTCCTTAG	GGTGACAACTTCAACGGCAA	-
y2571	GTGGAGACGAGTTTGTTTGATGATAATAAAGTCATCGCGTTGATTGGTTTGTGTAGGCTGGAGCTGCTTC	CATTCCAGGCTGTCGTTGAT	-
	TCAGTCCTCTTTATAACAGTTCTGCACATCAATAAGTTGTGCATCATTACCATATGAATATCCTCCTTAG	CAGCTAAGGAGCAGTGATAG	-
y2586-y2587	ATGGCTCGTTATTATCTCTCTGCGATGGATGTTTTATACCGCCTATCAATGTGTAGGCTGGAGCTGCTTC	CGGTGTGCAGTATCATGAGA	-
	TTAACCGTTATATATTCCAAACATCCGTGGCAACCACAGAGAGATTTCCGCATATGAATATCCTCCTTAG	TATTGGGGGAGAGAAACAGC	-
y2591-y2592	ATGGCTAAAGAAATATTCTGTGCGTTTGGCGTTGATGTTGACGCGGTGGCGTGTAGGCTGGAGCTGCTTC	AACTAGCGGGTTGTATCACC	-
	TTACTTCACACTCTGCGGTGCCACGTGGTGGGCAAACAGCATAATGACGCCATATGAATATCCTCCTTAG	CGCTAAGGATGCCAGTTACA	-
y2598-2600	ATGAATCTGAAAGGGAAGAAAGCCTTGGTCACTGCTGCCGGGCAGGGGATGTGTAGGCTGGAGCTGCTTC	AGGGTATCACGCGCTTTGAA	-
	TTAAACGCGAAAACGGTACTTCTCTATCGACGATTGATGCATTTCGATGGCATATGAATATCCTCCTTAG	TTCAAGCTGCTGGTGTGTTG	-
y2601-y2602	TTGACCCAGATTTCATTTTTTAATGCCCTTATTAGCCTGACCTTTAGTCCGTGTAGGCTGGAGCTGCTTC	TTAACGTAGCCAACGCACAG	-
	GTGACCGTAATAAAATACAGTGACTACAATTTTCGAACATCGTTAAACGCCATATGAATATCCTCCTTAG	TGATCAGCGCAGCCGTTAAA	-
y2616	ATGTATTTACGGCGTCTTTACGATCTGCCTGTTCTCTCCGTCGCGGGCACGTGTAGGCTGGAGCTGCTTC	TAAGGTGCAGCGCACAAATC	-
	TTAAATATGACGGGTATACTTATCTTTAATAGCACTGAGCTCACTACTTTCATATGAATATCCTCCTTAG	ATTTGCCGTTGAGGAGCAAG	-
y2632-y2642	GTGTCAAATACAGCACAGTTAATTACACCTTCCCCTATAGGGCTAATTGCGTGTAGGCTGGAGCTGCTTC	GCACAACAAGTCATTAGC	-
	ATGGTTTCACAACGTCAATTCAATGAAAATAATATCCAACATCTTAATGGCATATGAATATCCTCCTTAG	GGTAATGGCCTTTACTAT	-
y2660-y2661	ATGGCCGATATTAATACAGCACAACCTCGTGACTGGTTACTGGAAATGAGGTGTAGGCTGGAGCTGCTTC	ATTATTGGCGTGCAGGAG	-
	CTATTTCTTCTTCGCATACTTCAGTGAATCCAACGCAACCGCAAAGATGACATATGAATATCCTCCTTAG	CCCCCAGGGAAGCTATTTTT	-
y2770-y2771	TTACTGATTCTCTTCCTCCTTATTGCCTCCTTTCTTGGTATCTTGTGGATGTGTAGGCTGGAGCTGCTTC	TGGGCACCCAGTCAGCTTGA	-
	CTAATCCAACCAAGGGAGAAGCTGCCGAGCTGCATTATCAAATAAAGGGCCATATGAATATCCTCCTTAG	ACAGCCAACGGCAGGACAA	-
y2785-y2786	ATGTTTACAAAAAGTAATTTCAAAAAATCGGTTGTTATCATCACGGCAATGTGTAGGCTGGAGCTGCTTC	CTTTTTTGCGAAGGCTACGG	-
	TCATATGCCGATAGATAAACCCGTTAATAAACTAACACCACGTTGAGTATCATATGAATATCCTCCTTAG	ATAATTCGGGTTGCCGCAAG	-
y2787	ATGAAATATATAAAGCTAACGGTACTCGCCGGAATATTTGTAGGGATCAGGTGTAGGCTGGAGCTGCTTC	CTGATGCCAGAGCCGCTTTT	-
	TCAATATGTGTGGAATAGTTTTTGAATTTCTTGTGGTTTTTGAGTTTGCGCATATGAATATCCTCCTTAG	GGGGCGTCCATTCCATTACAA	-
y2809-y2812	CCCGTCTCCATACTTCTACGTCCCTCGCAAGGCTATTCTAGTTTGAGATGGTGTAGGCTGGAGCTGCTTC	CTTTGGGGCCTTCATGAACA	-
	TGATTACCACGCCAATGTCGTTGGCTGCCGCTTTCTAGCCCGTTGACTTACATATGAATATCCTCCTTAG	CGACCGAGTGTAAAAAGCAG	-
y2826-y2833	ATGAAAAAGAAAAATATTGCCCTCATCCTCCCTCTCGCTCTTTTACTTAGGTGTAGGCTGGAGCTGCTTC	GTTAACACACTATGCGGTGG	-
	TTATACCCCAAATAATTCGAGTGGCAGGAAGGCAGCCAACGCATAGGCAGCATATGAATATCCTCCTTAG	TCGGCATTCTCATCGCCATT	-
y2857	GTGAATATAAACGTCGCAAATTTGTTAAACGGCAACTATATCTTGCTGTTGTGTAGGCTGGAGCTGCTTC	CTTACTACTGGCTGTCTTGC	-
	CTAACCTAATATCCCTGGCCAGAGGATGACAATCAACGAGCCAGCCAAGGCATATGAATATCCTCCTTAG	ACCTGCCATACTGCAAACTG	-
y2862-y2863	GTGCACGCCATTTACCGCAATATCAATGAAAAAAAGAAGAAAAATAACAGGTGTAGGCTGGAGCTGCTTC	CCGTAGAAATCGATAGACCG	-
	GTGTTCACAATGGAGATTTTCATGGACACTACGCAAACAAGCACTCTTGCCATATGAATATCCTCCTTAG	CGCCATCAAAAACCAAGCTG	-
y2878	ATGGCGACTTCTCAGACAAAAGAACTCCCCCGCCCTCAGCTGTTTACTGTGTGTAGGCTGGAGCTGCTTC	ATGCTCCCTCGCCTATCGCT	-
	TTACACAAGTGCAATATGTTGCACAAATAAGTTGATACCGGTGCCACCGACATATGAATATCCTCCTTAG	CGGATACGGCCCTTAAACGG	-
y2880-y2882	ATGGAGACTGTTCTCATGAAAATGAAATGTTTTGCGAAAAATGCGCTGGCGTGTAGGCTGGAGCTGCTTC	TTAGTGGTGATCGGGAGTTG	-
	TTAATGGCATACTCCGCTGAGCATATACAGCCCGGTATCACCTTTTTCCGCATATGAATATCCTCCTTAG	ATTTAACAGCCGGGTTGACC	-
y2889	ATGGATAAGGCAACCGAGACCCGCCGCGAGGGAGCACCTACCGCGAGCAAGTGTAGGCTGGAGCTGCTTC	TCTGCTGATGGTTGATGAGC	-
	CTATCCGAAGTAACGGATCCGGAAAGCATCAATGGCGACAGCGATAAAAACATATGAATATCCTCCTTAG	CGAAAAGTGTCAGCAATGGC	-
y2892-y2894	ATGAGTGAGTATCATGAAAAAAATAATGCACTCTTCAAGTCGATTACCGCGTGTAGGCTGGAGCTGCTTC	AGGGGTCTATGTTTAGACCC	-
	TTATCGCAGTTGAACGTAGGAGGAACCGTCAAAATAGCCCACCACCATACCATATGAATATCCTCCTTAG	TCGAGTAAGGGATCGAAAGG	-
y2895-y2897	ATGCTGCCCACTTCACTCACCAAGGCCAAACTTGTTGGCCGAGTCTGGCGGTGTAGGCTGGAGCTGCTTC	ACCCCTGCTGGTTCGAACGA	-
	TCAGATGCTGGTATATCCCGCATCGGCAGTAATGATTGAACCTGTCAGCACATATGAATATCCTCCTTAG	CCGTGAAGAGGCGTTGGTGT	-
y2909	TTGTCATTACTGTATCGCAGCAGTGAAAATAAATTAACAACACAAAGTGGGTGTAGGCTGGAGCTGCTTC	AAAGCGGTAGCGCTATGCAA	-
	TTAAGCCAGTAAGTTTTTGTTACTCATCTTTTCATGACAGAATTTAGCTACATATGAATATCCTCCTTAG	CCAATCGCCGACAGAGTAAT	-
y2928-y2929	ATGAGCTTCAACCTGCCCGGAATGTATTTGTATTACCTTTCAAGAGATAAGTGTAGGCTGGAGCTGCTTC	AAGCAGCCAGCCAGATGAAT	-
	CTACTGGACATTAGCTTCACGGATCTCTGCCAATATAGCGGCAAGAAAATCATATGAATATCCTCCTTAG	TACTATCGGTTAACGTGCGC	-
y2949-y2950	ATGATGATGATCCCTGAACAGCGTCGTGACTTTATCTACCGCTACGTGCAGTGTAGGCTGGAGCTGCTTC	TACTATGTGATGCGCATCGG	-
	CTATGAAAACGCCTTGATATTTTTATTTTTTCCCAACCATCCCAACCCTGCATATGAATATCCTCCTTAG	CGCTGGGTGAGTCTCATTAA	-
y2958	ATGTTTTCCTTTCTATTAGGACACTCTATGCTTGATTCCATTCGCTCACGGTGTAGGCTGGAGCTGCTTC	CACAGTACATCTGCACACAG	-
	TTAGATCTTAAATTGATTAACGGTATCAGTTAAATCAGCGGCCTGAGCTTCATATGAATATCCTCCTTAG	AATTACACCAACCGGTGACG	-
y2961-y2963	GTGGCGGTAAACATGAGTAAACAGATTTCAACAAGGCATACGTTGGCTTAGTGTAGGCTGGAGCTGCTTC	CATTATGGTGGTAAGAGGGC	-
	ATGAATAATTTACATTACAAGGACGTATTGACTGGAAGAATGCCAAATGCCATATGAATATCCTCCTTAG	CATTCCTACAGCTCCACTGT	-
y2999-3000	ATGGTCACGCTGATCGCACTGATTGTTCTGCCTTTTTTGGCGCTGTTACCGTGTAGGCTGGAGCTGCTTC	TCATCGACTCCTTCTTGGTG	-
	TCAACGCCCTAAATACACCTCAATCACCTGATCATTCTCCTGTACCTCTGCATATGAATATCCTCCTTAG	CAAGTGATGCTGCCGTTCTT	-
y3035-y3037	GTGGAGAACAACATGAAGAATCAATACGGTAAAGTGAGTTACTGGGTAGCGTGTAGGCTGGAGCTGCTTC	CCGAAAGAACGGAATACCCT	-
	TCAATCTGGTACAGGGCCATTCTGGCGGTTAAATGACACACTTTTTATCTCATATGAATATCCTCCTTAG	TGAATCTCAGGGCCTGAGTA	-
y3043-3046	ATGTACGTTCTGGTAACAGGTGGTAGCGGTTACATTGGTAGCCATACCTGGTGTAGGCTGGAGCTGCTTC	CTTGCAACAACGCCTCGGTC	-
	TTATAAAGGCAACGCAATAAAGTGATAAACCGTCGTTGAGCGATAGGTCTCATATGAATATCCTCCTTAG	GCGTAGTCGGCAGCAGTGAA	-
y3154	ATGTCAAAACGATTACCACCCCTGAATGCCTTACGGGCTTTTGATGCCGCGTGTAGGCTGGAGCTGCTTC	TGTCTGGCATACCAGACCAT	-
	TCAGTTTTCATAACGAAAACGTAATTTCTCCTGCTCGCTGGCAGCTCTTGCATATGAATATCCTCCTTAG	CCCCAGAGAGGTGCTTAATA	-
y3183	TTGCGGGTAATCTTATTTTCCGCAAAACTGAGTGATGTAAGCGGTTACCCGTGTAGGCTGGAGCTGCTTC	AATTGCAGGCATTCTCGCAC	-
	TTAATCAGTGTCATCCCGTAGGCTTGGCGGGCTGGCCACAGAGTGGCGGCCATATGAATATCCTCCTTAG	AAGTTGGGGGTAACGGATGA	-
y3222-y3223	ATGATTGGGATTGTTGTTTCTGGCCACATTAATTTTGCTTCTGGTATGGCGTGTAGGCTGGAGCTGCTTC	AACAGTGGAGCTACAAACCG	-
	TTAGGCTGAGTGGCGATAAGCCCATTCACCTTTAATCAGGGTTGCGGCAACATATGAATATCCTCCTTAG	CCCGTATTACATCCGGTGAT	-
y3226-y3228	ATGGCACAGCCACATACTCAACAAGCCTATTTTCACATGATGGCCAAGCCGTGTAGGCTGGAGCTGCTTC	TGTTCTGTTTGCCCGTTCGC	-
	TTAAAAGAGTGACGTCATTTTTGAGCTGTAGCTGGTTACGCCTTTGCGGACATATGAATATCCTCCTTAG	AGCCTCCGATCTGTGCCTGA	-
y3249-y3250	ATGTTTGGTCATTCAAAACACGATCATCGTCATGGTGGTCATCGCCATGAGTGTAGGCTGGAGCTGCTTC	ACTGATATTGTCACTCGGCC	-
	CTATTCTGCATGATCACTACGGTCACGCGGTGAACCTTTCTTCAGTAGGACATATGAATATCCTCCTTAG	CCTGAATGATCCGGTCTCTT	-
y3289	GTGGAGAGTGGCATGGAAAACATCATAAAATATGATATTAATTATTTTTTGTGTAGGCTGGAGCTGCTTC	CTTGAAGTATGGCGGGTGTA	-
	TCACATAAAGCTGAGAAGTTGCGGTGCCAGGGATAAATAAATCATCACTACATATGAATATCCTCCTTAG	GTATTACCCGTGATGTACGC	-
y3302	ATGACTCAGGATGAACTTAAAAAAGCGGTGGGCTGGGCAGCATTAGATTAGTGTAGGCTGGAGCTGCTTC	GGCAATGCTGAGGTATTCAC	-
	TTAGCCAATCACTTTAACGCCATTGGCCGTACCAATCAGGGCCACATCAGCATATGAATATCCTCCTTAG	TCGAATGAATCCGGTCAGCA	-
y3389-y3390	ATGGCTTTGCTGGTTGGGATGACGATCATTATTGCTGCTGTGGGTATTATGTGTAGGCTGGAGCTGCTTC	CCTCCCTGTAACTCGACTTA	-
	TCACACCGATAAATCCTCAGAAAAAACCAAAGACAACGCCTCTACCGGCACATATGAATATCCTCCTTAG	GTTCAGTGCACGCTTGTGAC	-
y3418-y3423	TTGTCTCAGCGAGGTTATTGGGTGATCACCGCTGGGAGGCGGGAACATCCGTGTAGGCTGGAGCTGCTTC	CAAACTACTTTGCCGGGGAT	-
	CTATTCACCCAGTCTCCCTGGTTGCCAGGCTTGTGCTTGCTGTTGTAATGCATATGAATATCCTCCTTAG	CAACGTTGAGCTATGGGTAG	-
y3435	ATGGCTGACGCACCTTTATCGACTGCTGCACCGCCCTCAATCTCCAGGCGGTGTAGGCTGGAGCTGCTTC	AATTGGGCACGGCTCCTAAT	-
	TTAATACCAGAGATACCAGGCTAACAGCGAGACAACGATGATGCAGATGACATATGAATATCCTCCTTAG	ACTGAGTGATGATGCCGTGA	-
y3474-y3475	TCAGCTCTTGCAAATCGCCAGTTTATCGCCGGAGAGGGTATTGATGTTCAGTGTAGGCTGGAGCTGCTTC	GGCGAATAAGCGTGCTTCTA	-
	TCAGGAGACTTGTTCGGTTGTCAGCATCTCACCACTCACCAAAACGCTATCATATGAATATCCTCCTTAG	TAATTTCCCGGTCGATCCGA	-
y3509	ATGAGTAACGTACTAATTATTAATGCAATGAAAGAGTTTGCCCATTCTAAGTGTAGGCTGGAGCTGCTTC	ATCGGCACCGCAAGTTCATT	-
	TTAGCTGTTAACGTTTTCGGCTAAGTGCTGACGATAGCGGGCAATATCCCCATATGAATATCCTCCTTAG	AAGGGATTGCCGAACTCTCT	-
y3519	ATGAACACCTTTCGTATGCCTGCACTGTTTCTCGGCCACGGTAGCCCGATGTGTAGGCTGGAGCTGCTTC	GTCAACACCGGGTTGTTGTT	-
	TTAGCCGATTTGCACCGACAACATACTTAACGACCCCATCTCAACCCCATCATATGAATATCCTCCTTAG	GACGGGTATACCCAAAGTCA	-
y3525	ATGCGAAGGGGATACAAGGAATCTGATATGTTGCCACTCCCTTCGGAATTGTGTAGGCTGGAGCTGCTTC	CCGTATATCGATGCCTGGTT	-
	TTAGCTCACATTAGCGTCGTTTGCCGCCAACCATTTATGCCAACTGGCCGCATATGAATATCCTCCTTAG	TCGACTTTCACTACAGGCAC	-
y3569	GTGATGACAGGAGTGATGTTACATAACGTCAATAAAATATATCCAAACGGGTGTAGGCTGGAGCTGCTTC	AAACAACGTGAGTCAACCGC	-
	TTAGATATAGAGAATATTTTTTTCGCTTTCTTTATCGAAAAGATGGCACTCATATGAATATCCTCCTTAG	ACTGGCGCAGGAAGATATTG	-
y3576-y3577	ATGAGCATGGTGGTGGTCGGCAGCGGCGGCTCTGCGAATCTGAGCATGAGGTGTAGGCTGGAGCTGCTTC	TGTGGAGAATAAACAGCGGG	-
	TTATGTATAAAGACTATCGTCGTAAGGCGATTCAGCCAGAATTTTTTGAACATATGAATATCCTCCTTAG	GTTGAACTCGACGGCGATAT	-
y3641	ATGATTCATAAATTACATCAATATCACCTGGTACGTAGGTTGCGTCAGCAGTGTAGGCTGGAGCTGCTTC	GATCGCTCGTTCAGTTCAGT	-
	TTAATCGCTATACATCAAGTCAATTTCATTCTGATAGCGTTGAAGAATGACATATGAATATCCTCCTTAG	TGGTCACGCGCTGAAATTAC	-
y3654	ATGGAAGCCTATTTATTACATTTATTGACGCAATCCCTGGCTTTCACGCTGTGTAGGCTGGAGCTGCTTC	AGAATTCGACTTCGGTGCCT	-
	TTACCCAGCCAAGACTTTCCACAACAGATGGCGATAGATTGGCATCAGTGCATATGAATATCCTCCTTAG	ATCAGGTGTGCATAAACCGC	-
y3707	ATGAACACCATCAAGTTGGCGAATCCTGGCCCATTGGGCCTTATGGGCTTGTGTAGGCTGGAGCTGCTTC	TTGCTGACGTATGTATCCGC	-

[illegible]	TTACATTACAGCTTGGGTCTCTAAGGTTTGGCTCGGTTCACCAATAGGCACATATGAATATCCTCCTTAG	ATCACTATCGAGCCGATGAG	-
y3755	GTGATATATGATATTGGTTATGATTATCATTATCATTTGGGTGGGCATCAGTGTAGGCTGGAGCTGCTTC	GATCGTAGGAGTGGCTATTC	-
	TTATCCCCGATGATCGGATACCCCCATCATGACACACCACCGTGCATTAGCATATGAATATCCTCCTTAG	AAGTCTCTAGAATTCGCCCC	-
y3776	TTGACATTTTTATCTCATAGCCGCGAGGGGATAGCTGCTTTTATTCTGGTGTGTAGGCTGGAGCTGCTTC	TTCATCCGTGTGCGATGAGT	-
	TCACAGTTCGAGCTCGTGTTTCAGCAGCATTTCTACTTGCTGGGCCGTCCCATATGAATATCCTCCTTAG	ATGGCACCACGGCGATTAAA	-
y3777-y3782	ATGATGAAAGAGCTGATAAATATACTTAAAAATACGCGCCAACATCTGATGTGTAGGCTGGAGCTGCTTC	ATTGCCGGGAAGTTGAGATG	-
	ATGCCGATTCCTGAGCGTTTAACACCAGGCAAAGCCACTAAAAATCGGACCATATGAATATCCTCCTTAG	ATCGCAGAGGAAGTACATCG	-
y3891	TTGGCTTATTTGTTGGTTTTTGATTACAATCGTGAGCGAAAACGAACATTGTGTAGGCTGGAGCTGCTTC	CCCTCTAGATTCAGGGTTTC	-
	TTAAGAAACCAGCGGCAGCGCCTGTTGTTTTTCAGTGTGCGCATCAGCCACATATGAATATCCTCCTTAG	TTTCTCTTCAGACCGGACGA	-
y3927- y3931	ATGCTGATCCAAGGTATTGGCTTGGCTCTCTATCTGGTGGTGGTGCTAATGTGTAGGCTGGAGCTGCTTC	GAGTCGTTGCCGGAAAACAC	-
	GTGACTGGCCCCCCGCTGCGGGTACGCGGTAATGAGGCATTACTGGATGACATATGAATATCCTCCTTAG	TCAGGTTAGCCAGTGCTTCT	-
y3950	ATGAGCACGTTTAAAATAACCATTATTGGCGGTGGGAGTAGCTATACCCCGTGTAGGCTGGAGCTGCTTC	CGGTAGCCATTTTTGCGAGA	-
	TCACAAACCGGAGAACTGAGGCAGGTAAGGTTTATTCACCTGTAGTACCTCATATGAATATCCTCCTTAG	GACGTCTTCCAGTGTAACCA	-
y4034-y4038	TTGAAGGAAATTGCCGTGACGACTCAAAACAGATTCCGAGATAATGAGATGTGTAGGCTGGAGCTGCTTC	CCAGCGCGCAATTTTGAGTT	-
	TTAAATTGCGGGTGTAAGATGGCGATCAGGGGCCTGGCACCAGTTATTTTCATATGAATATCCTCCTTAG	CTGCTTGGCTGAGCTAAACT	-
y4043-y4046	ATGCGATTACGTTTAGCTTTATTTTCGTCATTACTGGCGGCAACCTTTGCGTGTAGGCTGGAGCTGCTTC	TAATCCTGCTCTGGCGCTGC	-
	TTAGCGATAATAAACCGCGATATGCTGGCCTTGTACTTTTTCTATGCTTACATATGAATATCCTCCTTAG	CCGAATATGGGGCTGACCAG	-
y4045-y4047	ATGCATTACAGCTTATTGGTAATACTTGCTGTGGGTTATCGGGTTCGTTCGTGTAGGCTGGAGCTGCTTC	GCATGGGAGCAACATAGAAG	-
	TTGCCAGCAAAGCGGTTATTGCTACTGAGCATCATCGCTCTCGGTGCGATCATATGAATATCCTCCTTAG	TATGGCGATGGGCGATAATC	-
y4050	TTGGCTATTTCCACCGTACTGATGCTCAAACGCGTCTCTTGCCTCGTTCTGTGTAGGCTGGAGCTGCTTC	CCGCAGAAGAAAACCAGAGA	-
	TTAACTGGCGCGCGTTCGCGGCGGGATCAGTGACTTGATTTTCTTGCGCCCATATGAATATCCTCCTTAG	GGATCATCGCTATAGTCTCC	-
y4063	TTGTACTCATTATTAATAATTCTATTATGTGATTGGTATTTCAGAGATATGTGTAGGCTGGAGCTGCTTC	GCCTGAATTCTGCCCATCAA	-
	TTATTTATAATTCAGAGCATAGGTTGCCTGAGAGACAACAGTACCGGTAGCATATGAATATCCTCCTTAG	GAGGGGTGTAATCATCAACC	-
y4067	ATGCAAACTTCATTTTCACCCGCGACACGCTTAGGTCGACGGGCGCTTTTGTGTAGGCTGGAGCTGCTTC	GCTTTTGACATCAACAGCCC	-
	TTACTCGGTAGCAACCGCCTCGGGTTGGCGGCGGATGAACACCATCACCACATATGAATATCCTCCTTAG	CAGCCATTTTGTCACCTTGC	-
y4100-y4101	GTGGCTACCTATTATCTCGCTTCAAAGAAGGAGTTAACGTATATGCGTAAGTGTAGGCTGGAGCTGCTTC	CCCGAAAGTTTGAAACCACG	-
	CTACAACGTCCAGAACAATACCGCCAATATCTGTGGTGACATGATTCGTACATATGAATATCCTCCTTAG	CTATGACTGGCCACATACTC	-
y4057-4058 (xylAB2)	ATGCAATCTTATTTTAATGAATTAGAACAAGTGCGCTACGAAGGTAGCCAGTGTAGGCTGGAGCTGCTTC	GCCTAAACAATTCGAGTGGC	-
	CTACTCACACAATGGCAGCAATTGTTGATAAAGTTTTTTAAAGGTTACCCCATATGAATATCCTCCTTAG	AAAGCTGGGCCAGTTTGACG	-
y2893 (xylB1)	ATGCAGGCGCTTTTGGGCATTGATATCGGCACCTCGGGGTGTAAGGCGTTGTGTAGGCTGGAGCTGCTTC	ATAACGTCGATGCCAGCATG	-
	CTACATAAAGGTGGGGTCGCCAAGTTCTGCGAAGGTTTCTCTCAGCGCCGCATATGAATATCCTCCTTAG	AAAGAAATGCGAGGTGGTC	-
y0856-55 (sgbU-lyK)	ATGCGCATTCACCCACTAGGAATATACGAAAAAGCCCTACCTGCGGGCACGTGTAGGCTGGAGCTGCTTC	CCCCAAAGTCATTGGCGTTC	-
	TTACATTACCGTGCTGAGCGCTTCAGCGACCTGTTTGAATTTGTCAAAACCATATGAATATCCTCCTTAG	CTTATCGAGGGTTCAGCCAG	-
y2095-94 (araAB)	ATGACAGGCAACGTGATATCGGCAGACGGTGCTATAGCTCTCGGGTTGGAGTGTAGGCTGGAGCTGCTTC	GTGCATACTTCCAGCTATTG	-
	TTAGCGATTGAGCTGATAGTACACCTCGTTCCAACGGATTTCGTTTTTGACATATGAATATCCTCCTTAG	GCATTGTCATCACCCTCAGG	-
y2072 (araD2)	GTGCAGGATTTCCAGCCGATTTGTACGCAGTTTTTAGCTATTTACCCCATGTGTAGGCTGGAGCTGCTTC	GAATAATCCTAATTTGGCGC	-
	TTATTGGCCGTAGTACGCATCCTTGCCATGTTTACGTAAGTAATGCTTATCATATGAATATCCTCCTTAG	TCAATCGACGCTATTGCACAG	-
y0863 (araD1)	ATGACATCATTCGAATACCGAATGATGTTTTTCATTATCGCCTGCCCATGTGTAGGCTGGAGCTGCTTC	GAGTATCTGCCAATAATCGC	-
	TTATTGGCCGTAATACGCCGCAGCCCCATGCTTACGCAAATAATGCTTATCATATGAATATCCTCCTTAG	AGAACGCATCCCGATGAGTC	-
y1646 (idnK2)	ATGAAGAATCTATTTTCGTTGGAAAATCGCAAAGTATTAATTACTGGCTCGTGTAGGCTGGAGCTGCTTC	CAAGAAGCGGAGCAACTCCC	-
	TCATAAAAGATGATGATTAGCCACCTTCACCACTAGCTTACGGATGGGGGCCGCATATGAATATCCTCCTTAG	CGTCTGAAGGGAGCATGGCT	-
y3876 (gntV/indk)	CTAAGCCAACGCCCCCACGCATCGCCGGACAACCTCATCAATATCAGTATGTGTAGGCTGGAGCTGCTTC	CGTTCATACTCTTCGGGGCG	-
	ATGAAGAAAAAAAGGCCAGTGCTACAAGATGTCGCCGATATGGTGGGAGTCATATGAATATCCTCCTTAG	AAGGGTATGCACATCGCGCT	-
y3838 (kdgK)	ATGACCAGCAAAAATATTGCCGTTATCGGCGAATGCATGATTGAACTGTCGTGTAGGCTGGAGCTGCTTC	CGATTAACGCGAATGGAGCT	-
	TCAAGCAGTTGGCATGGCGGCCAGAGGGATAATGGCACCACGGTACTGGACATATGAATATCCTCCTTAG	TGCAGGAGATCTATAACGGC	-
y2629 (gnd)	TTGGCCTTAAGGCTGGTAACAGGTGTCATCCTTCAGACAGGAGTTTTTTTGTGTAGGCTGGAGCTGCTTC	GCCGAGAATCCAGGTAAGAA	-
	TTACGCCATCCACTCAGTGTGGAATACACCTTCTTTATCTGTCCGCTTATCATATGAATATCCTCCTTAG	GCACGAATAATACAGCCAGC	-
y3938 (rpe)	TTGGAGAAACGGATGAAAAAGTATTTAATTGCCCCCTCTATTCTGTCAGGTGTAGGCTGGAGCTGCTTC	TGCAAGGCTCTGCGTGATTA	-
	TTAACCATTAGCCGCCATCGCCAGTTCATTGCGCATGGCATCGATCACCGCATATGAATATCCTCCTTAG	GGATGTCATAACCGCTGTCA	-
y3712 (talB)	ATGACCGATAAACTTACTTCCCTACGTCAAATCACCACTGTAGTCGCCGAGTGTAGGCTGGAGCTGCTTC	GGGATATTGCGGCGATGAAA	-
	TTACAACAAGTCGGAGATCATTTTCTCAAGTTTGCCTTGGTCGATAGCAACATATGAATATCCTCCTTAG	GATGCCGTCTGCCAGTTTAT	-
y3310 (tktA)	ATGTCCTCTCGTAAAGAGCTTGCCAACGCTATCCGCGCACTAAGCATGGAGTGTAGGCTGGAGCTGCTTC	GGTTTTGCTATCGCAGAACG	-
	TTACTTCAGCAGCGCCTGAGCTTTCGCCACCACGTTTTCCACGGTGAAACCATATGAATATCCTCCTTAG	CGAACACGCTACGGATTTTC	-
y0357-rhlB	ATGAGCAAAACACACTTGACTGAACAGAAGTTTTCCGACTTCGCCCTGCAGTGTAGGCTGGAGCTGCTTC	TATCGCTCATGTTCTACTCC	-
	CTAAGCTCTTCCAATAGCAGTGGCCATTGAACATAACTTTGCCAGTGACTCATATGAATATCCTCCTTAG	TAGTGAAGATAATAACGGGC	-
y0329-y0331	ATGGAAATATTGTTAGAAGTCCGTGGCTTATCTGTTGAGTTTCCTGGTGTGTGTAGGCTGGAGCTGCTTC	TGCTCAAGAAAGACGGTACC	-
	TTACACGGGAGTAAGTGGCGATGACTGAGGGCGTGATCTGCGGTTGAGATCATATGAATATCCTCCTTAG	ATTGAAAGCGTAGCCATGGC	-
y1324 (ail)	ATGGTTTTTATGAATAAGACATTACTGGTCTCTTCTTTAATTGCATGTTTGTGTAGGCTGGAGCTGCTTC	TTTGTCACGGCAATGGTCGC	-
	TTAGAACCGGTAACCCGCGCCAAGCATCCAAGTACCCACTTTCACATCATCATATGAATATCCTCCTTAG	CGCGGTGAAGGACGGATTCT	-
y1325 (hcaT)	ATGGTATTGCAATCAACACGTTGGCTATCGCTCAGCTATTTCACCTATTTGTGTAGGCTGGAGCTGCTTC	TGCTCAAGAAAGACGGTACC	-
	CTGGCTAATTCATTTGGGTTCGCGGCCACTCGTGGCCGGATAAACAGCGCCATATGAATATCCTCCTTAG	GCCATGGCTACGCTTTCAAT	-
y1326-28	ATGCGCCTAAACACGCCCTTTAAGCCTCGGTTATCTGATGATCAACCGCTGTGTAGGCTGGAGCTGCTTC	GCGCATTATTCAACCCTTGG	-
	GGCAGTTCCAATATGCGGCATAACTGAATACTTTGCGGCTCAGTTAACGGCATATGAATATCCTCCTTAG	GTATTGCGGTGATGACAGTG	-
y1329-30	ATGCGCTATGTGTTACATTATAACGGTTTTATTACAGTCAGCCGGCTGATGTGTAGGCTGGAGCTGCTTC	CTTAAAGGGCGTGTTTAGGC	-
	TCAGCGACCAAATGGACGAACAGCCCCAACCATCTCCGGCTGACTGGACACATATGAATATCCTCCTTAG	GCTATTGGTTCGTGAGTCTG	-
y0356 (rep)	GTGGCTTTCTGGTACAATTCGCTTCCCCGTAAACCCATTGAGCAACCGAGCATATGAATATCCTCCTTAG	AAACGTCACGGTTGATGGTC	GGCCAGAATGTCATTTGCCT
	CTACGGTTTCTTCGCATTCGCTAACTGAGCACGAAGATTGGCCAGATGACGTGTAGGCTGGAGCTGCTTC	TTGCCATAGTAACAGTCACC	TTCACTACTGTAACTGCTGC
y0332 (hdfR)	GTGGATACCGAATTACTGAAAACCTTTTTAGAGGTCAGTAGAACTCGTCAGTGTAGGCTGGAGCTGCTTC	GCTCATTGAAAGCGTAGCCA	CATGGCGCTCTAGTAATTGC
	TTACTCCCGTGTAACGCTCTGGGTCGCGTTATTCATGGGGGTTTTTAAGACATATGAATATCCTCCTTAG	TTTCGACGGTACACTCAGCT	TTTCGACGGTACACTCAGCT
y3891 (glpD)	TTGGCTTATTTGTTGGTTTTTGATTACAATCGTGAGCGAAAACGAACATTGTGTAGGCTGGAGCTGCTTC	CCCTCTAGATTCAGGGTTTC	CAAAGCTTAGATTCAGGGTTTCGTTCCAGA
	TTAAGAAACCAGCGGCAGCGCCTGTTGTTTTTCAGTGTGCGCATCAGCCACATATGAATATCCTCCTTAG	TTTCTCTTCAGACCGGACGA	CAGGATCCTGAGGTGCTTGAACAACGCTAT
y3302 (rpiA)	ATGACTCAGGATGAACTTAAAAAAGCGGTGGGCTGGGCAGCATTAGATTAGTGTAGGCTGGAGCTGCTTC	GGCAATGCTGAGGTATTCAC	GGCAATGCTGAGGTATTCAC
	TTAGCCAATCACTTTAACGCCATTGGCCGTACCAATCAGGGCCACATCAGCATATGAATATCCTCCTTAG	TCGAATGAATCCGGTCAGCA	GGGCGACAGGTTAATCAAAG
y2892 (rpiA2)	ATGAGCAATCAACAAAATGACGCCAAGAGGGCTGCCGCCCGTCGGGTGATGTGTAGGCTGGAGCTGCTTC	TTTCTTTGTGCGCGAACTGG	GAAACCTTCGCAGAACTTGG
	TTATCGCAGTTGAACGTAGGAGGAACCGTCAAAATAGCCCACCACCATACCATATGAATATCCTCCTTAG	CCGCAATGGCAATCCAGAAT	CTGCATGCTGTGATACTCTC
ymt	ATGCTTCAAATAGATAATGTCATTAATAATATTGGAAACTACTTTCATGTGTAGGCTGGAGCTGCTTC	ACACCACCAATGGCGATACT	
	TCAATTTGGGCTTAATTTTGGAAATGCATGTGGGCTAGAATATTTCCACATATGAATATCCTCCTTAG	GAGGGCGCTGGGTAATTGAA	
