# Supplementary material for: A refined model of how Yersinia pestis produces a transmissible infection in its flea vector
Source: PLoS Pathog. 2020 Apr 15;16(4):e1008440. doi: 10.1371/journal.ppat.1008440 (PMC7185726; doi:10.1371/journal.ppat.1008440)
Supplement: S1 Text — (PDF) [file ppat.1008440.s013.pdf]

DETERMINING THE PRESENCE OF A FREE-FLOATING PROVENTRICULAR CAST IN THE MIDGUT.

The proventriculus is composed of three regions: a small anterior region, a rounded region containing many spines, and a stomodaeum valve (Fig. S2A). Hence, a free proventricular cast located in the midgut will have taken on the shape (in full or in part) of the proventriculus. Although bright-field microscopy shows some obvious free-floating proventricular casts, fluorescence microscopy highlights the presence of bacteria in the free-floating mass and thus reveals the shape of the proventriculus - even when the mass appears to be shapeless under the bright-field microscope. The criteria used to determine whether a free-floating mass is a proventricular cast or not are described below, and Figure S2B shows several practical examples.

The first criterion is the presence of a comet/mushroom-like structure, where the head and the tail resemble the spined and stomodaeum regions, respectively. Unsurprisingly, the exact shape, breadth and length of the head and tail vary from one cast to another (Fig. S2A and the second and third columns of Fig. S2B). The second criterion is the presence of between one and three sharp edges, respectively representing the boundaries between the anterior and spined regions, the spined and stomodaeum regions, and the stomodaeum region and the midgut (red arrowheads and red lines in Fig. S2A and S2B). When one edge is present, we assume that it represents the boundary between the anterior and spined part of the organ. The third criterion is the presence of bacteria within the mass. The mass's colonization pattern should match the pattern observed in the proventriculus. Hence, the shape of a mass located in the midgut can be superimposed on the shape of the proventriculus. The fourth criterion relates to the fact that the shape of the bacterial colonization within the mass can be superimposed on or complements the shape of the proventriculus (Fig. S2B, the top-most two rows, with pink arrowheads). Lastly, it should be noted that the mass sometimes appears to be have been dislocated and needs to be put together again, like a jigsaw puzzle (Fig. S2C).

The Δ*tktA* mutant is highly deficient for *in vitro* growth. Five milliliters of LB in a 15 mL Falcon tube and an LB agar plate were inoculated using stock that had been frozen at -80°C. Bacterial growth was determined after overnight growth in LB incubated at 21°C or 28°C with shaking, using optical density (OD) measurements on a spectrophotometer. Bacterial growth on the LB agar plate was determined by eye after 2 or more days of incubation at 28°C. After incubation at 28°C and 21°C, the OD values were respectively 0.31 and 1.17 for growth medium inoculated with the WT strain and 0.03 and 0.04 for medium inoculated with the Δ*tktA* mutant. On the LB agar plate, WT and Δ*tktA* colonies could be detected at 2 and 4 days post-inoculation, respectively.

REFERENCES FOR SUPPLEMENTARY DATA

1. Romier C, Ben Jelloul M, Albeck S, Buchwald G, Busso D, Celie PH, et al. Co-expression of protein complexes in prokaryotic and eukaryotic hosts: experimental procedures, database tracking and case studies. Acta Crystallogr D Biol Crystallogr. 2006;62(Pt 10):1232-42. Epub 2006/09/27. doi: 10.1107/S0907444906031003. PubMed PMID: 17001100.

2. Datsenko KA, Wanner BL. One-step inactivation of chromosomal genes in *Escherichia coli* K-12 using PCR products. Proceedings of the National Academy of Sciences of the United States of America. 2000;97(12):6640-5. Epub 2000/06/01. doi: 10.1073/pnas.120163297. PubMed PMID: 10829079; PubMed Central PMCID: PMCPMC18686.

3. Pradel E, Lemaitre N, Merchez M, Ricard I, Reboul A, Dewitte A, et al. New insights into how *Yersinia pestis* adapts to its mammalian host during bubonic plague. PLoS pathogens. 2014;10(3):e1004029. Epub 2014/03/29. doi: 10.1371/journal.ppat.1004029. PubMed PMID: 24675805; PubMed Central PMCID: PMCPMC3968184.

4. Bartra SS, Styer KL, O'Bryant DM, Nilles ML, Hinnebusch BJ, Aballay A, et al. Resistance of *Yersinia pestis* to complement-dependent killing is mediated by the Ail outer membrane protein. Infect Immun. 2008;76(2):612-22. PubMed PMID: 18025094.

5. Hinnebusch BJ, Cherepanov P, Du Y, Rudolph A, Dixon JD, Schwan T, et al. Murine toxin of *Yersinia pestis* shows phospholipase D activity but is not required for virulence in mice. International journal of medical microbiology. 2000;290(4-5):483-7.
